# TNP and its analogs: Modulation of IP6K and CYP3A4 inhibition

**DOI:** 10.1080/14756366.2021.2000404

**Published:** 2021-12-11

**Authors:** Seulgi Lee, Bernie Byeonghoon Park, Hongmok Kwon, Vitchan Kim, Jang Su Jeon, Rowoon Lee, Milan Subedi, Taehyeong Lim, Hyunsoo Ha, Dongju An, Jaehoon Kim, Donghak Kim, Sang Kyum Kim, Seyun Kim, Youngjoo Byun

**Affiliations:** a Department of Biological Sciences, KAIST, Daejeon, South Korea; b College of Pharmacy, Korea University, Sejong, South Korea; c Department of Biological Sciences, Konkuk University, Seoul, South Korea; d College of Pharmacy, Chungnam National University, Daejeon, South Korea; e KAIST Institute for the BioCentury, KAIST, Daejeon, South Korea; f Biomedical Research Center, Korea University Guro Hospital, Seoul, South Korea

**Keywords:** Inositol hexakisphosphate kinase, cytochrome P450 3A4, structure-activity relationship

## Abstract

Inositol hexakisphosphate kinase (IP6K) is an important mammalian enzyme involved in various biological processes such as insulin signalling and blood clotting. Recent analyses on drug metabolism and pharmacokinetic properties on TNP (*N*^2^-(*m*-trifluorobenzyl), *N*^6^-(*p*-nitrobenzyl)purine), a pan-IP6K inhibitor, have suggested that it may inhibit cytochrome P450 (CYP450) enzymes and induce unwanted drug-drug interactions in the liver. In this study, we confirmed that TNP inhibits CYP3A4 in type I binding mode more selectively than the other CYP450 isoforms. In an effort to find novel purine-based IP6K inhibitors with minimal CYP3A4 inhibition, we designed and synthesised 15 TNP analogs. Structure-activity relationship and biochemical studies, including ADP-Glo kinase assay and quantification of cell-based IP7 production, showed that compound **9** dramatically reduced CYP3A4 inhibition while retaining IP6K-inhibitory activity. Compound **9** can be a tool molecule for structural optimisation of purine-based IP6K inhibitors.

## 1. Introduction

Inositol phosphates (IPs) have been recognised as second messengers that are involved in various biological processes ranging from growth to apoptosis^1^^,^^2^. Of these, inositol pyrophosphates such as 5-diphosphoinositol pentakisphosphate (5PP-IP5, abbreviated 5-IP7) harbour ‘high-energy’ diphosphate groups at 1- or 5-position of inositol hexakisphosphate (IP6, phytic acid). 5-IP7 is known to serve as a key signalling molecule critical for controlling insulin secretion, vesicle trafficking, growth signalling, telomere length regulation, migration, and cellular energy dynamics^3–6^. In mammals including humans, biosynthesis of 5-IP7 is catalysed by a family of three isoforms of IP6 kinases (IP6K1, IP6K2, and IP6K3). IP6Ks utilise ATP and IP6 to form a phosphoester bond, thereby 5-IP7 is produced^7^.

Genetic deletion of IP6Ks in cell lines and mouse models has revealed physiological roles of 5-IP7. For example, IP6K1 knockout (KO) mice exhibit hypersensitive insulin signalling, lengthened blood clotting, altered presynaptic vesicle cycling, and resistance to high-fat diet-induced obesity^8–12^. IP6K2 was found to promote cancer cell migration and tumour metastasis throughout inhibition of tumour suppressor liver kinase B1 (LKB1)^13^. While IP6K1 and IP6K2 are ubiquitously expressed, IP6K3 is detected in the cerebellum and skeletal muscle^14^. When IP6K3 is deleted, KO mice demonstrated metabolic alterations such as lower blood glucose and decreased fat mass, with extended lifespan^15^. These findings thus suggest the therapeutic benefit of IP6K inhibition in managing obesity, diabetes, as well as longevity.

**Figure 1.**
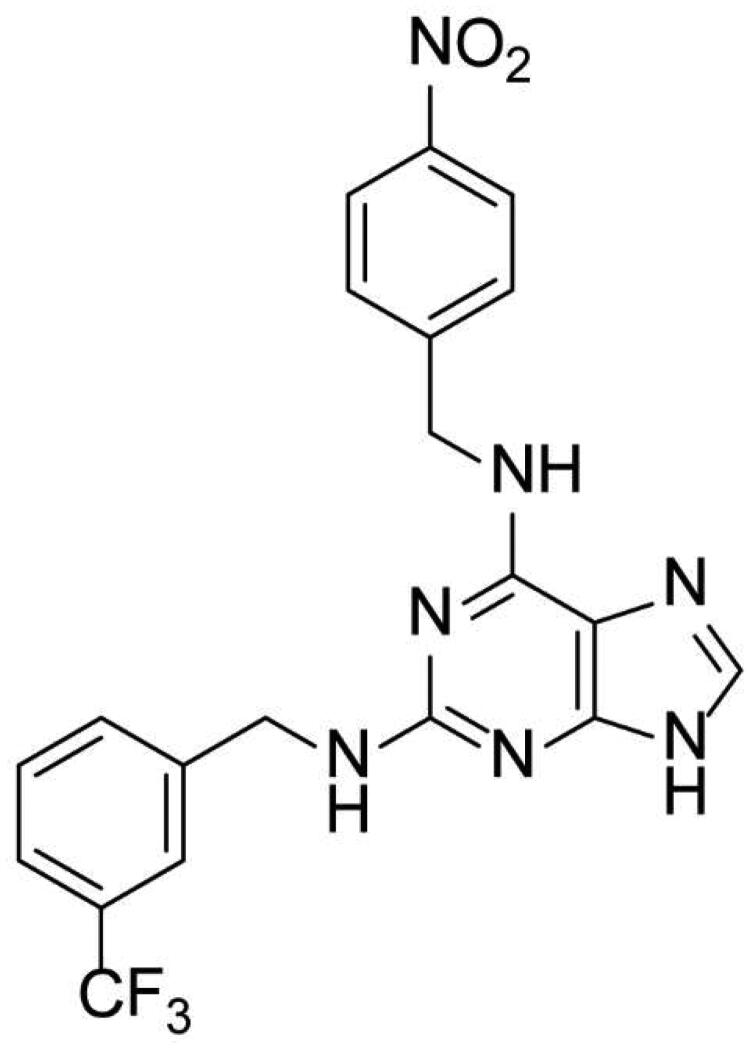
Chemical structure of TNP.

Padmanabhan et al. first discovered *N*^6^-[(4-nitrophenyl)methyl]-*N*^2^-[[3-(trifluoromethyl)phenyl]methyl]-9H-purine-2,6-diamine (TNP, Figure 1) as a pan-IP6K inhibitor acting in an ATP competitive manner^16^. TNP has been widely used in a number of studies to examine the role of 5-IP7 in the control of various biological events *in vitro* and in vivo levels. Importantly, TNP treatment led to the protection of mice from high-fat diet-induced weight gain and accompanied metabolic dysregulations. These metabolic actions of TNP appear to be mediated by depleting 5-IP7 which suppressed Akt-dependent insulin signalling in diet-induced obesity^8^^,^^17^. Anti-obesity effect of TNP further supports the role of 5-IP7 metabolism in the control of thermogenic energy expenditure in the adipose tissue^17^^,^^18^.

Accordingly, TNP ameliorates obesity and insulin resistance in vivo. While TNP was considered as a useful tool compound, it can certainly be improved particularly in terms of potency, solubility, and IP6K isoform selectivity to maximise its therapeutic value^16^^,^^17^. Furthermore, recent analyses of TNP on drug metabolism and pharmacokinetic (DMPK) properties revealed that TNP may inhibit human cytochrome P450 enzymes including CYP1A2, CYP2C9, CYP2D6, and CYP3A4^17^, indicating that inhibition of CYP450 by TNP might lead to unfavourable drug-drug interactions in the liver. However, the precise action of TNP on CYP450 metabolism remains unclear.

In this work, we screened for TNP effects of various CYP450 isoform enzymes involved in drug-drug interactions. Based on CYP inhibition studies using human liver microsomes and LC-MS/MS quantification experiments, TNP was found to be a potent inhibitor of CYP3A4. TNP binds to CYP3A4 in type I mode. Therefore, we focussed on modifying the chemical structure of TNP to reduce off-target binding with CYP3A4 while maintaining IP6K-inhibitory activity. Based on structure-activity relationship studies and biochemical assessments including ADP-Glo kinase assays, quantification of IP7 production, and AKT phosphorylation in cell-based assays, we discovered new purine-based IP6K inhibitors with a weaker CYP3A4 inhibition than TNP.

## 2. Results and discussion

### 2.1. CYP3A4 inhibition of TNP

TNP is a purine-based IP6K inhibitor harbouring a 4-nitrobenzyl group at the *N*-6 position and a 3-(trifluoromethyl)benzyl group at the *N*-2 position. TNP was first synthesised and characterised as an ATP-competitive IP3K inhibitor^19^, but TNP was later found to have selective inhibitory activities towards IP6K (IC_50_ = 0.47 μM; [ATP] = 1 mM) rather than IP3K (IC_50_ = 18 µM; [ATP] = 10 μM)^17^. TNP also modulates effectively both kinase and phosphatase activities of IP6Ks producing 5-IP7 and IP5^20^. Since TNP substantially decreases cellular 5-IP7 levels^17^, it has been recognised as a useful pharmacological tool compound for targeting IP6K activity.

**Figure 2.**
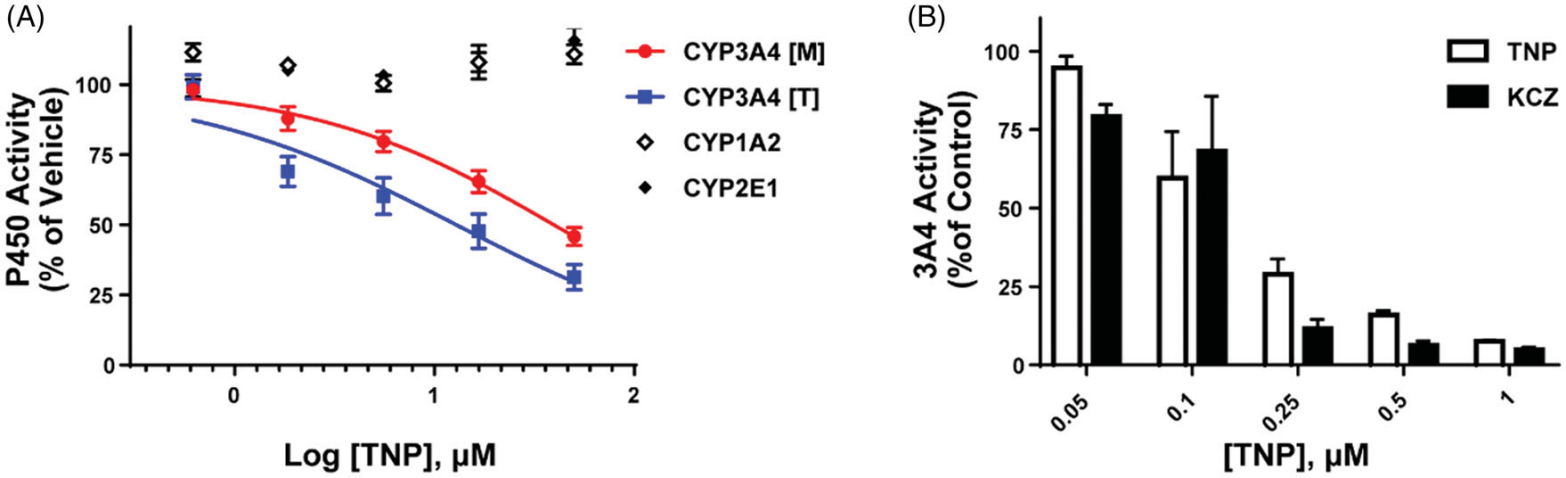
The selective inhibitory effect of TNP on CYP3A4. (A) Screening the activities of human microsomal CYP450 isoforms. The CYP450 metabolic capacities were measured by LC-MS/MS and isoform-specific substrates (phenacetin for CYP1A2, chlorzoxazone for 2E1, midazolam [M] for 3A4, and testosterone [T] for 3A4). (B) In vitro analysis of recombinant microsomal CYP3A4 against TNP or ketoconazole (KCZ) treatment with designated concentrations on graphs. Values in all graphs are presented as mean ± SEM.

The inhibitory potential of TNP against activities of nine CYP isoforms (CYP1A2, 2A6, 2B6, 2C8, 2C9, 2C19, 2D6, 2E1, and 3A4) was evaluated using pooled human liver microsomes (HLMs). Except for CYP3A4, TNP had no inhibitory effect on other CYP isoforms (CYP1A2, 2A6, 2B6, 2C8, 2C9, 2C19, 2D6, and 2E1) up to 50 μM (Figure 2(A), See Supplementary Figure 1). CYP3A4 activity, as determined by the formation of 1-hydroxymidazolam from midazolam or 6β-hydroxytestosterone from testosterone, was dramatically inhibited by TNP treatment (Figure 2(A))^21^^,^^22^. The IC_50_ values of TNP against CYP3A4 were 65.4 μM and 31.4 μM using midazolam and testosterone as substrates, respectively (Figure 2(A)).

To avoid the effects of the other CYP450 isozymes-mediated metabolism, the inhibitory potentials of TNP in reactions using a single recombinant microsomal CYP450 isozyme were also measured. Consistent with the CYP450 inhibition assay with HLMs, TNP has a specific capacity to inhibit CYP3A4 metabolism. The IC_50_ value of TNP against CYP3A4 was 149.3 nM, which was comparable to ketoconazole (IC_50_ = 123.1 nM, Figure 2(B)), a potent CYP3A4 inhibitor. The difference in the IC_50_ values of CYP3A4 between HLMs and recombinant CYP3A4 is due to the different compositions and substrates for the assay. HLMs contain a collection of CYP isozymes and other drug metabolising enzymes (e.g. flavin-containing monooxygenases, esterases, and microsomal epoxide hydrolase) and use different substrates (5 μM midazolam and 50 μM testosterone for HLMs, 10 μM fluorogenic cyanocoumarin for recombinant CYP3A4). IC_50_ values are dependent on the types and concentrations of substrates.

### 2.2. Type I binding of TNP against CYP3A4

**Figure 3.**
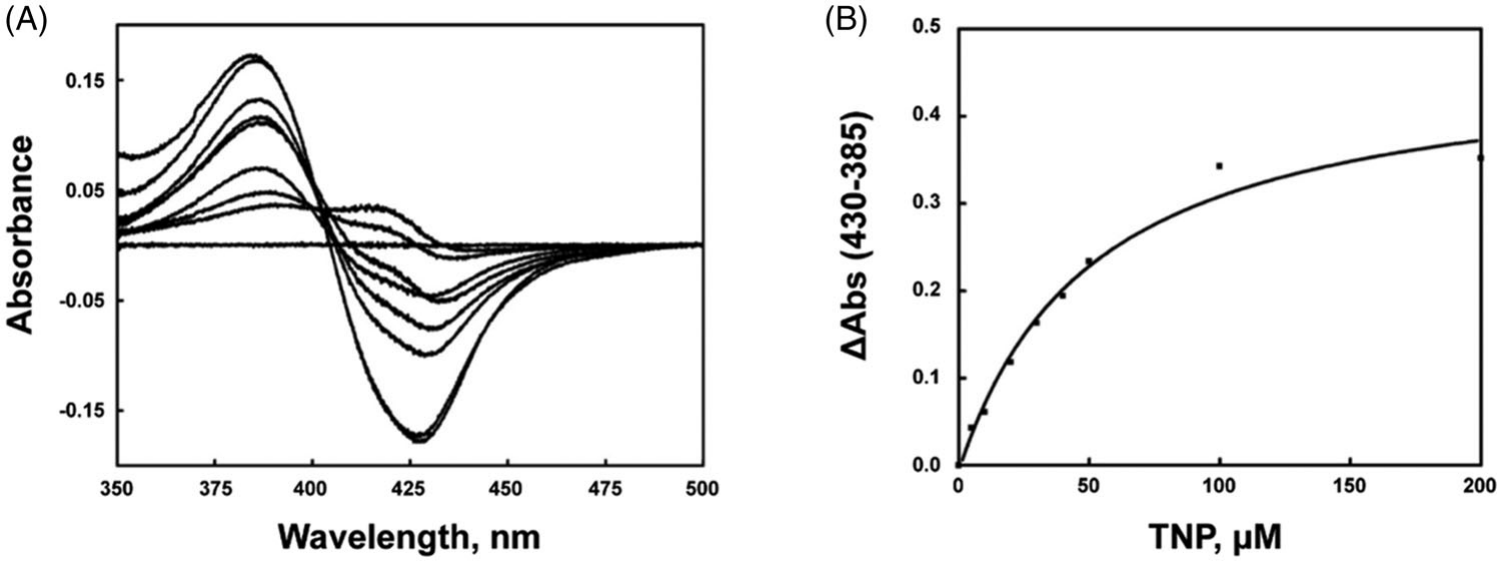
Interaction of CYP3A4 and TNP. (A) Type I spectral shift induced by TNP interaction with CYP3A4. (B) Determination of TNP dissociation constant.

The spectral binding titration of purified CYP3A4 with TNP was performed. TNP displayed a typical Type I spectral change with an increase at 385 nm and a decrease at 430 nm (Figure 3(A)), suggesting the replacement of water molecule with TNP to the CYP450 heme^23^. Although most of the CYP450 inhibitors containing nitrogen atoms display the type II binding titration, the type I binding of TNP could occupy the substrate-binding site of CYP450 by competing against the substrates of CYP3A4, resulting in the inhibitory effect of CYP3A4 catalysis reaction. The calculated *K*_d_ value of TNP was 54.7 ± 8.6 µM (Figure 3(B)).

### 2.3. Synthesis of purine-based analogs

**Scheme 1.**
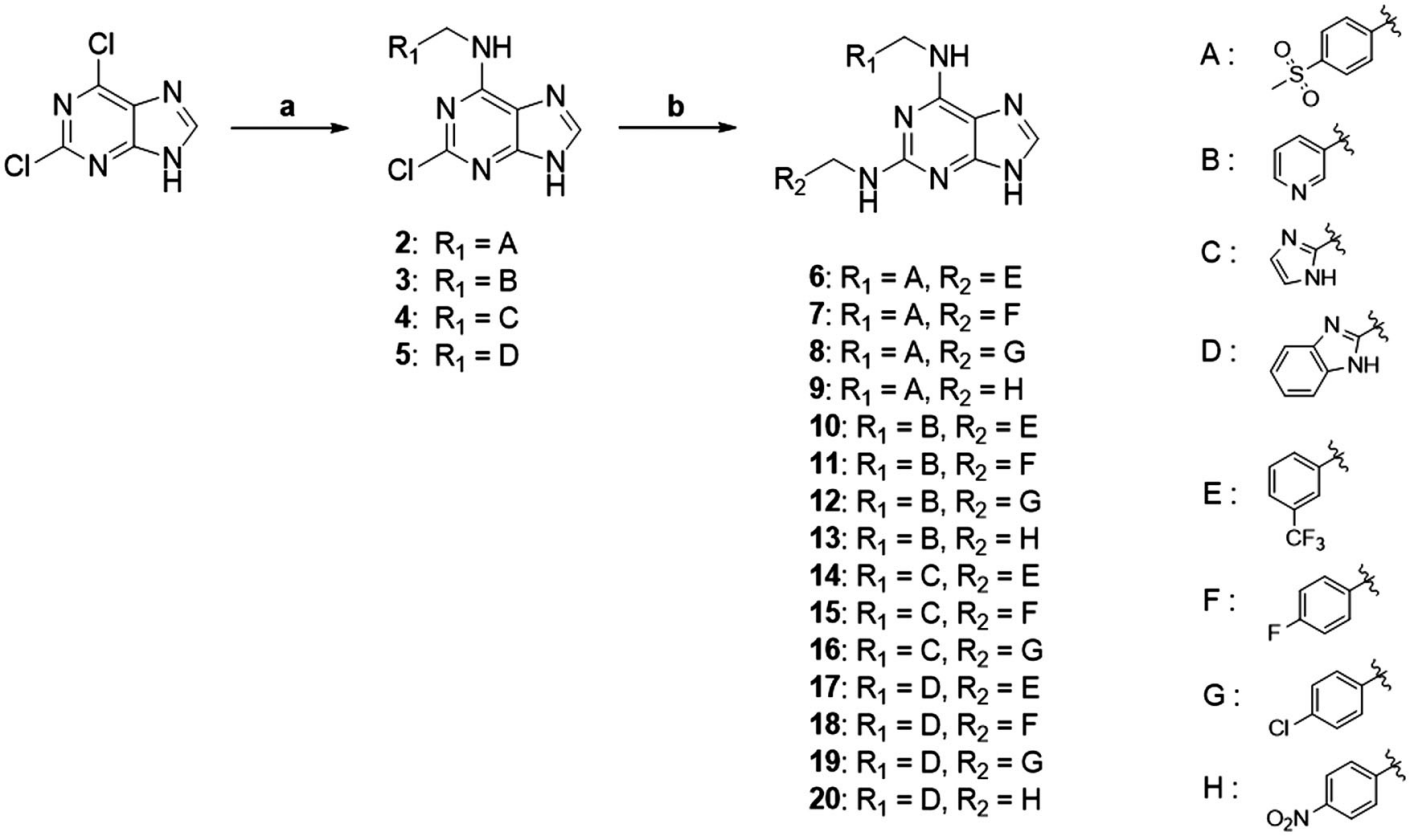
Synthesis of purine-based TNP analogs. Reagents and conditions: (a) appropriate benzylamines (1.1 eq), Et_3_N (1.1 eq), DMF, 100 °C, 8 h; (b) appropriate benzylamines (5.0 eq), n-butanol, NaBF_4_ (1.5 eq), 180 °C.

New purine-based analogs were synthesised from commercial 2,6-dichloropurine in 2 steps as described in Scheme 1. The reaction of 2,6-dichloropurine with substituted benzylamines in DMF in the presence of triethylamine provided the corresponding 6-substitued purine analogs **2–5** in 70–85% yield. Recrystallization from a mixture of ethanol and water afforded compounds **2–5** in high purity (>95%). When aprotic polar solvents such as DMF and DMSO were used, the reaction of compounds **2–5** with mono-substituted benzylamines (4-fluoro, 4-chloro, 4-nitro, and 3-trifluorobenzylamine) was not successful. However, *n*-butanol as a solvent in combination with sodium tetrafluoroborate (NaBF_4_) as a reaction facilitator afforded 2,6-disubsituted purine analogs (**6–19**). When microwave reaction was applied, the reaction time (3 h) was greatly reduced as compared to the conventional reflux condition (30 h).

### 2.4. Spectral binding titration of the synthesised compounds with CYP3A4

Binding titration patterns of TNP analogs with purified CYP3A4 enzyme were analysed (Table 1). Compounds (**6**–**9**) substituted with the sulphonyl group at 4-position of the phenyl ring in R_1_ position showed Type I binding pattern as observed in TNP. Except compound **9**, compounds **6**–**8** have a stronger binding affinity for CYP3A4 than TNP (Table 1). Compound **9** did not show any binding spectral change at 200 μM. In addition, relative CYP3A4 inhibition of **9** at 100 nM was 71% as compared to TNP (Table 1). Compounds (**10**–**13**) with the pyridine ring in the R_1_ position showed a typical Type II binding pattern in which the nitrogen atom in the pyridine ring might be coordinated with the iron atom in the haem of CYP3A4 enzyme. These compounds showed strong relative CYP3A4 inhibition (∼130%) as compared to TNP (Table 1). In the case of imidazole analogs, compounds **14** and **16** showed a Type II binding pattern while compound **15** did not display any binding spectral change up to 200 μM (Table 1). Benzimidazole-substituted compounds (**16**–**19**) exhibited Type I binding spectra, indicating the competitive inhibitory mode of substrate site in CYP3A4. However, compound **20** with a 4-nitrobenzyl group in the *N*-6 position did not show any spectral dissociation constant value for CYP3A4 (Table 1).

**Table 1.** CYP3A4 inhibition and IP6K2 IC_50_ of purine-based TNP analogs.
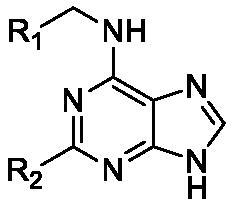

Entry	R_1_	R_2_	CYP3A4 *K*_d_ (μM)^a^	CYP3A4 binding mode	Relative 3A4 inhibition (% of TNP)^b^	IP6K2 IC_50_ (μM)^c^
	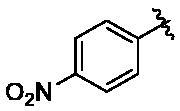	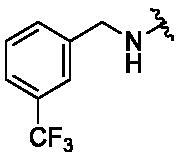	54.7 ± 8.6	I	100	4.5 ± 0.4
6	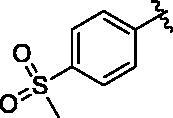	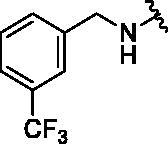	24.8 ± 4.3	I	105 ± 9	4.6 ± 0.2
7	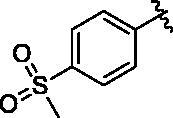	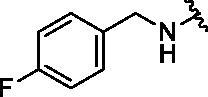	28.8 ± 2.7	I	55 ± 3	7.3 ± 0.5
8	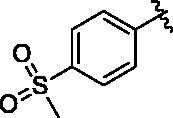	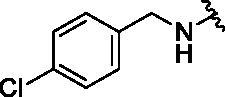	14.4 ± 3.3	I	94 ± 1	ND^e^
9	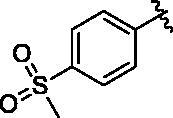	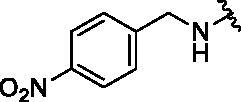	No binding^d^	–	71 ± 3	16.8 ± 0.5
10	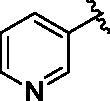	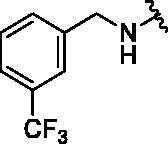	1.4 ± 0.5	II	134 ± 1	5.3 ± 0.3
11	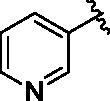	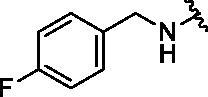	3.0 ± 0.6	II	131 ± 1	5.9 ± 0.2
12	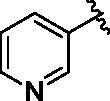	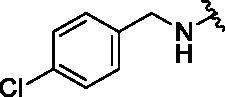	1.8 ± 0.6	II	133 ± 1	ND^e^
13	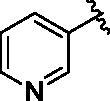	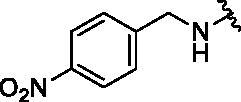	2.0 ± 0.5	II	126 ± 1	3.3 ± 0.8
14	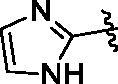	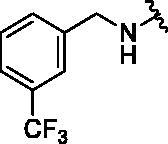	22.2 ± 4.5	II	97 ± 1	34.7 ± 0.7
15	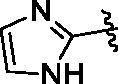	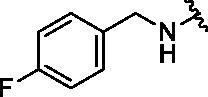	No binding^d^	–	64 ± 4	24.7 ± 0.2
16	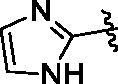	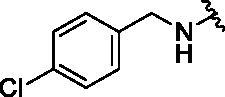	40.5 ± 8.0	II	99 ± 2	19.9 ± 0.1
17	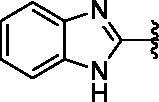	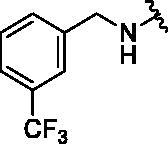	7.9 ± 0.8	I	116 ± 3	12.6 ± 0.4
18	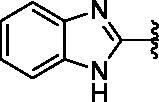	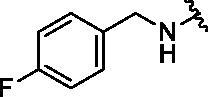	11.1 ± 1.0	I	120 ± 1	13.2 ± 0.4
19	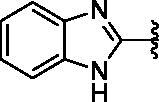	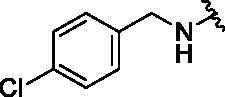	16.9 ± 3.7	I	120 ± 0.66	19.3 ± 0.27
20	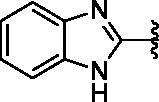	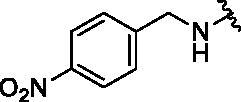	No binding^d^	–	65.7 ± 1.97	21.8 ± 0.30

^a^*K*_d_ values displayed spectral dissociation constant of TNP analogs on CYP3A4.

^b^The inhibitory potency of the analogs against a recombinant CYP3A4 compared to TNP after 100 nM treatment for 10 min. The % inhibition was calculated using the equation: % inhibition = [1–(*R*′_′Test_ –*R*′_TNP_)/(*R*_Neg_–*R*′_TNP_)] × 100. *R* means reactions rates (fluorescence per 10 min). All values are presented as mean ± SEM.

^c^The analogs were testes in an 8-dose IC_50_ mode in triplicate with 2-fold serial dilution. IC_50_ values are depicted as mean ± SEM.

^d^No binding spectral change was observed up to 200 μM of the compounds.

^e^ND: not determined.

### 2.5. IP6K2-inhibitory activities of the synthesised compounds

To determine IP6K2-inhibitory properties of the synthesised TNP analogs, *in vitro* ADP Glo kinase assay with purified human IP6K2 was performed. As shown Table 1, all of the compounds exhibited IP6K2 inhibition with IC_50_ values from 3.3 to 34.7 μM (Table 1). Under the same experimental condition, IC_50_ value of TNP against IP6K2 was 4.5 μM. Surprisingly, compounds (**9**, **15,** and **20**) without CYP3A4-binding affinity showed moderate IP6K2-inhibition with IC_50_ values of 16.8, 24.7, and 21.8 μM, respectively (Table 1). Although IP6K2-inhibitory activities of these compounds were reduced slightly as compared to TNP, they displayed much lower CYP3A4-inhibitory activities than TNP. This result suggests that these compounds can be prototype molecules to optimise new purine-based IP6K2 inhibitors without inhibiting CYP3A4.

### 2.6. Evaluation of new IP6K2 inhibitors in cell-based assay

**Figure 4.**
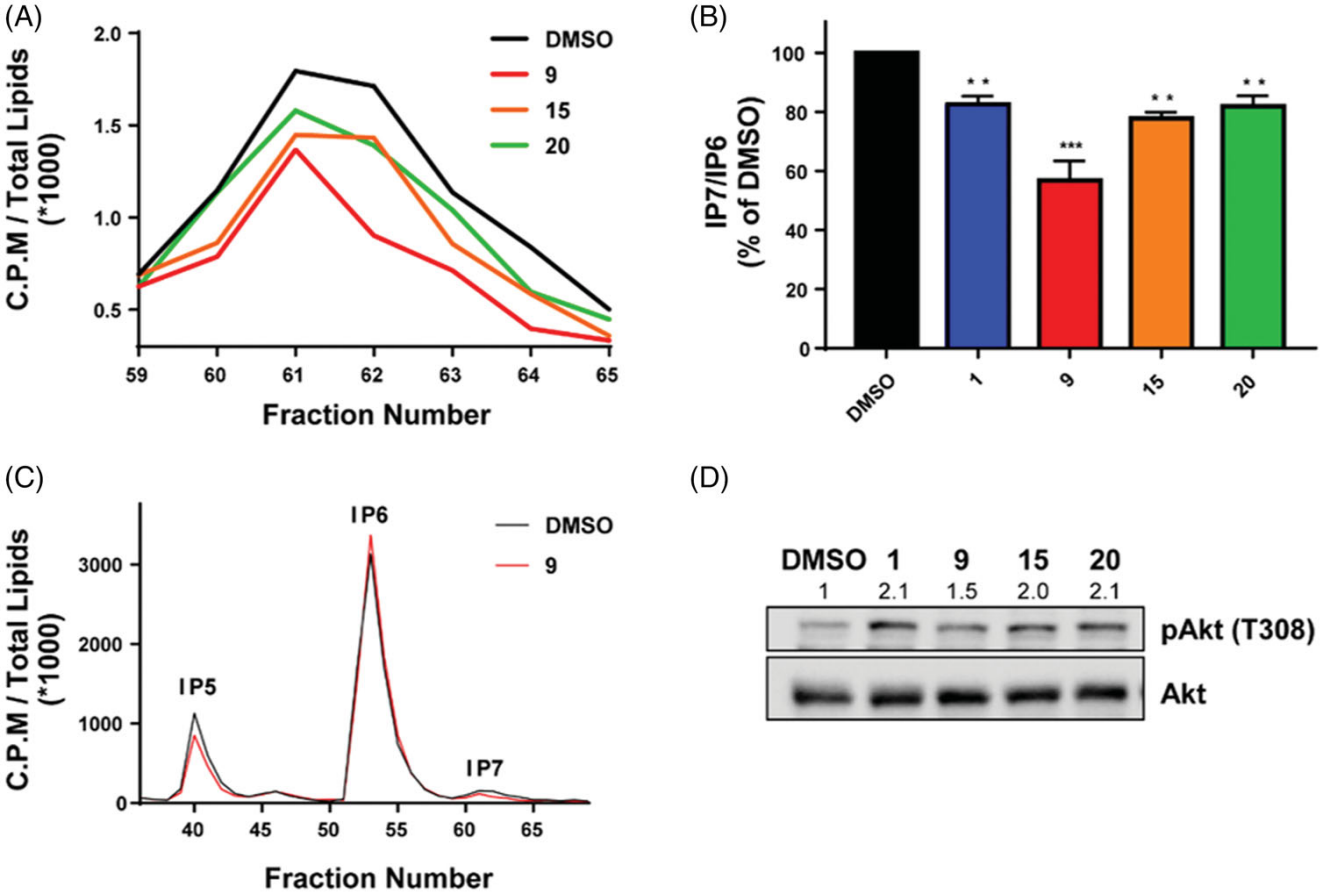
The cellular effects of three compounds (**9**, **15**, and **20**) on IP7 synthesis and Akt activation in the HCT116 cell line. (A–C) HPLC analysis of cellular IPs (IP5, IP6, and IP7) and IP7/IP6 ratio from radiolabeled HCT116. The effects of the tested compounds on (A) IP7 quantification and (B) IP7/IP6 ratio of three compounds and TNP. (C) The inhibitory effect of compound **9** on IP7 synthesis. (D) Akt activation (phosphorylation on Thr308) was analysed by Western blotting with HCT116 cell extracts. Representative immunoblot with the relative levels of pAkt/Akt (an arrowhead) using Image J is shown. All experiments are prepared from HCT116 treated with DMSO, TNP (10 μM), and the selected compounds (50 μM) for 4 h. All values are presented as mean ± SEM. Student's *t* test was used for statistical analysis. *P < 0.05, **P < 0.01, ***P < 0.001.

To examine whether the three candidate compounds (**9**, **15**, and **20**) can inhibit IP7 synthesis in cells, intracellular IP7 levels in human colorectal cell line (HCT116) after-treatment of the compounds (50 μM) for 4 h were measured since IP6K2 has been reported as a major enzyme for cellular IP7 synthesis among three isoforms of IP6Ks in HCT116^24^. We found that the three compounds reduced cellular IP7 synthesis without notable changes in IP4, IP5, and IP6 levels (Figure 4(A–C) and Supplementary Figure 2). In particular, compound **9** showed a more potent inhibitory effect (∼ 44% decrease compared to DMSO) on cellular IP7 synthesis than compounds **15** and **20** (Figure 4(B)). To further validate the impact of the selected compounds in controlling 5-IP7-dependent cellular signalling, we analysed the phosphorylation status of Akt which is known to be inhibited by 5-IP7^8^. Treatment of HCT116 cells with the three compounds led to at least a more than 1.5-fold increase in Akt phosphorylation at Thr308 compared to vehicle treatment (Figure 4(D)). Collectively, these results demonstrate that compounds **9**, **15**, and **20** act as efficacious human IP6K inhibitors, thereby depleting cellular levels of 5-IP7 and accompanying biological outcomes such as enhanced Akt activation.

### 2.7. Docking studies of compound 9

**Figure 5.**
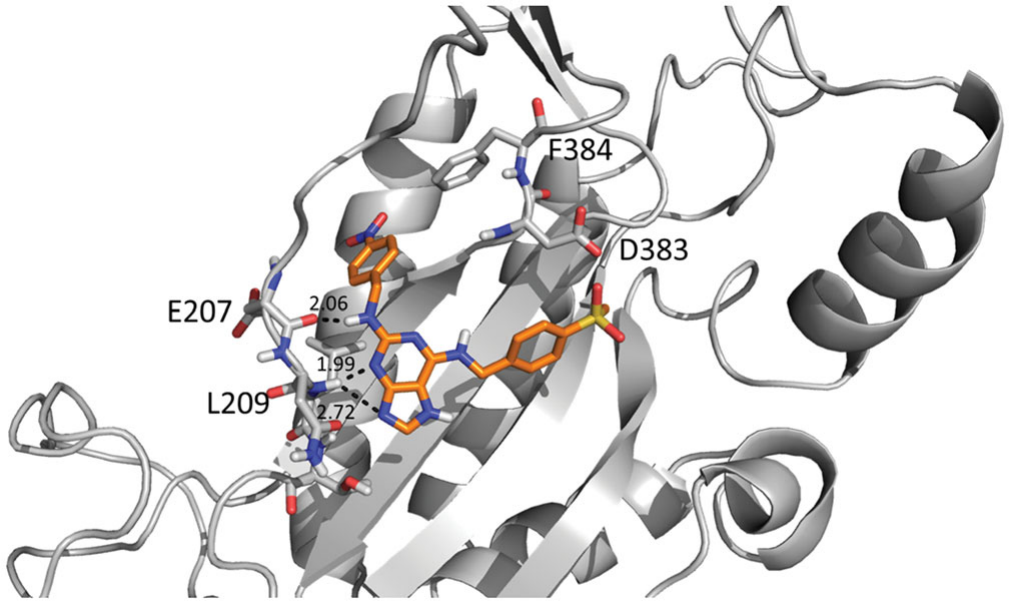
The best-docked pose of compound **9** with an IP6K homology model. The dotted lines represent hydrogen-bonding interactions.

Molecular docking studies of the most potent compound **9** and TNP were performed with an IP6K2 homology model to better understand the binding mode of **9** in the active site of IP6K2. A homology model of IP6K2 was prepared by using the SWISS-MODEL module based on the *Eh*IP6KA X-ray crystal structure (PDB ID: 4O4D) as a template^25^. Docking studies of compound **9** and TNP revealed that their binding poses are similar and are located in the ATP-binding site. Since TNP is an ATP-competitive inhibitor of IP6K, we assumed that compound **9** could also bind to IP6K2 with a mechanism of action similar to TNP. The purine ring of TNP and **9** formed hydrogen bonds with active site amino acid residues such as Glu207 and Leu209 (Figure 5 & Supplementary Figure 4).

## 3. Conclusions

We screened inhibitory effects of TNP, a reported pan-IP6K inhibitor, on a variety of CYP450 isoform enzymes. By employing biochemical and spectrophotometric analyses, we identified that CYP3A4 is a major CYP450 isoform to be inhibited by TNP. TNP binds to CYP3A4 in a type I pattern with a *K*_d_ value of 54 μM. To remove the CYP3A4-inhibitory activity of TNP, we designed and synthesised new purine-based TNP analogs. Our medicinal chemistry efforts and biochemical evaluation identified three compounds (**9**, **15**, and **20**) as moderate IP6K2 inhibitors without binding to CYP3A4. Among them, compound **9** displayed the strongest IP6K inhibition with an IC_50_ value of 16 μM without binding to CYP3A4 up to 200 μM. We observed the cellular effect of compound **9** in HCT116 cell-based assay. Compound **9** enhanced Akt phosphorylation by reducing the production of IP7 in human cells. Our structure-activity relationship studies against CYP3A4 and IP6K2 suggest insights for future development of IP6K inhibitors with minimising drug-drug interaction mediated CYP3A4. Overall, compound **9** can be a tool molecule for further structural optimisation of novel purine-based IP6K2 inhibitors.

## 4. Experimental section

### 4.1. General

All the chemicals and solvents used in the reaction were purchased from Sigma-Aldrich, TCI, or Alfa Aesar, and were used without further purification. Reactions were monitored by TLC on 0.25 mm Merck precoated silica gel plates (60 F_254_). Reaction progress was monitored by TLC analysis using a UV lamp and/or KMnO_4_ staining for detection purposes. Column chromatography was performed on silica gel (230–400 mesh, Merck, Darmstadt, Germany). ^1^H and ^13 ^C NMR spectra were recorded at room temperature (298 K) in CDCl_3_ (7.26/77.16 ppm), CD_3_CN (1.94/118.26 ppm), D_2_O (4.79 ppm), or CD_3_OD (3.31/49.0 ppm) on either Bruker BioSpin Avance 300 MHz NMR or Bruker Ultrashield 600 MHz Plus spectrometer and referenced to an internal solvent. Chemical shifts are reported in parts per million (ppm). Coupling constants (*J*) are given in Hertz. Splitting patterns are indicated as s, singlet; d, doublet; t, triplet; q, quartette; m, multiplet; br, broad for ^1^H NMR data. High-resolution mass spectra (HRMS) were recorded on an Agilent 6530 Accurate mass Q-TOF LC/MS spectrometer. Low-resolution mass spectra (LRMS) analyses were obtained from an API 150EX ESI-MS spectrometer. High-performance liquid chromatography purification was performed on Agilent 1260 Infinity (Agilent). The purification of synthesised compound was performed on a semi-preparative reverse-phase high-performance liquid chromatography (RP-HPLC; Agilent 1260 series HPLC instrument) using a semi-preparative column (Phenomenex Gemini-NX C18, 110 Å, 150 mm × 10 mm, 5 μm) over 30 min at a flow rate of 2 ml/min. A suitably adjusted gradient of 5% B to 95% B was used, where solvent A was 0.1% formic acid in H_2_O and B was 0.1% formic acid in acetonitrile. UV detection was carried out at 220 nm and 254 nm. The structure identification of each HPLC fraction was carried out by an electrospray ionisation PE Biosystems SciexApi 150 EX mass spectrometer single quadruple equipped with a turbo ion spray interface. The purity of all final compounds was measured by analytical reverse-phase HPLC on an Agilent 1260 Infinity (Agilent) with a C18 column (Phenomenex, 150 mm × 4.6 mm, 3 μm, 110 Å). RP-HPLC was performed on two different solvent systems using the following isocratic conditions: for method A, the mobile phase was acetonitrile and water (25:75, v/v, 0.1% formic acid); for method B, the mobile phase was methanol and water (50:50, v/v, 0.1% formic acid). All compounds were eluted with a flow rate of 1.0 ml/min (method A) or 0.7 ml/min (method B) and monitored at UV detector: 254 nm. The purity of the tested compounds was >95%.

### 4.2. Synthesis

*General procedure for the synthesis of 6-substituted purine analogs (2 − 5)*: To a solution of 2,6-dichloropurine (300 mg, 1.58 mmol) in DMF (7 ml) were added appropriate amine (1.74 mmol) and triethylamine (1.66 mmol). The reaction mixture was stirred at 100 °C for 6–8 h. After the completion of the reaction, the excess solvent was removed under reduced pressure. Recrystallization from ethanol/water afforded 6-substituted purine analogs (**2–5**) as solid in 70–85% yield.

#### 4.2.1. 2-Chloro-N-(4-(methylsulfonyl)benzyl)-9H-purin-6-amine (2)

85% yield. mp 271 °C. ^1^H NMR (300 MHz, DMSO-*d*_6_): *δ* 12.96 (brs, 1H), 8.79 (brs, 1H), 8.16 (s, 1H), 7.87 (d, *J* = 8.1 Hz, 2H), 7.58 (d, *J* = 7.8 Hz, 2H), 5.27 (brs, 1H), 4.75 (s, 2H), 3.33 (s, 3H). HRMS (ESI) calcd for C_13_H_13_ClN_5_O_2_S^+^ [M + H]^+^: 338.0473, found: 338.0539.

#### 4.2.2. 2-Chloro-N-(pyridin-3-ylmethyl)-9H-purin-6-amine (3)

71% yield. mp 220 °C. ^1^H NMR (300 MHz, DMSO-*d*_6_): *δ* 13.05 (brs, 1H), 8.72 (brs, 1H), 8.58 (s, 1H), 8.44 (d, *J* = 4.5 Hz, 1H), 8.15 (s, 1H), 7.75 (d, *J* = 7.2 Hz, 2H), 7.37 − 7.30 (m, 2H), 5.18 (brs, 1H), 4.66 (s, 2H). HRMS (ESI) calcd for C_11_H_10_ClN_6_^+^ [M + H]^+^: 261.0650, found: 261.0680.

#### 4.2.3. N-((1H-imidazol-2-yl)methyl)-2-chloro -9H-purin-6-amine (4)

76% yield. mp 178 °C. ^1^H NMR (300 MHz, MeOH-*d*_4_): *δ* 8.34 (s, 1H), 8.10 (s, 1H), 7.18 (s, 2H), 4.95 (s, 2H). HRMS (ESI) calcd for C_9_H_9_ClN_7_^+^ [M + H]^+^: 250.0603, found: 250.0595.

#### 4.2.4. N-((1H-benzo[d]imidazol-2-yl)methyl)-2-chloro-9H-purin-6-amine (5)

83% yield. mp 264 °C. ^1^H NMR (300 MHz, MeOH-*d*_4_) *δ* 8.98 (s, 1H), 8.28 (s, 1H), 7.80 − 7.73 (m, 2H), 7.52 − 7.45 (m, 2H), 5.13 (s, 2H). HRMS (ESI) calcd for C_13_H_11_ClN_7_^+^ [M + H]^+^: 300.0759, found: 300.0771.

*General procedure for the synthesis of 2,6-disubstituted purine analogs*: To a solution of 6-substituted purine analogs (0.15 mmol) in butanol (5 ml) were added appropriate benzylamines (0.75 mmol) and sodium tetrafluoroborate (0.23 mmol). The reaction mixture was stirred at 180 °C using microwave irradiation (75 min) or a sealed tube in an oil bath (3 h). After the completion of the reaction, the excess solvent was removed under reduced pressure. The crude product was purified by reversed-phase HPLC using water/acetonitrile (0.1% formic acid for each) to give the corresponding 2,6-disubstituted purine analogs.

#### 4.2.5. N^6^-(4-(methylsulfonyl)benzyl)-N^2^-(3-(trifluoromethyl)benzyl)-9H-purine-2,6-diamine (6)

This compound was purified by reverse phase HPLC using 0.1% formic acid in acetonitrile (A)/water (B). The gradient consisted of 10% A to 90% A in 20 min at 2 ml/min flow rate. *Rt* = 11.53 min. 38% yield. mp 103 °C. ^1^H NMR (300 MHz, MeOH-*d*_4_): *δ* 8.37 (brs, 1H), 7.83 (d, *J* = 8.4 Hz, 2H), 7.73 (s, 1H), 7.61 (s, 1H), 7.55 − 7.32 (m, 5H), 4.79 (s, 2H), 4.59 (s, 2H), 3.09 (s, 3H). HRMS (ESI) calcd for C_21_H_20_F_3_N_6_O_2_S^+^ [M + H]^+^: 477.1315, found: 477.1319.

#### 4.2.6. N^2^-(4-fluorobenzyl)-N^6^-(4-(methylsulfonyl)benzyl)-9H-purine-2,6-diamine (7)

This compound was purified by reverse phase HPLC using 0.1% formic acid in acetonitrile (A)/water (B). The gradient consisted of 10% A to 90% A in 30 min at 2 ml/min flow rate. *Rt* = 12.69 min. 43% yield. mp 151 °C. ^1^H NMR (300 MHz, MeOH-*d*_4_): *δ* 7.85 (d, *J* = 8.1 Hz, 2H), 7.75 (s, 1H), 7.60 − 7.47 (m, 2H), 7.32 − 7.17 (m, 2H), 7.10 − 6.90 (m, 2H), 4.84 (s, 2H), 4.50 (s, 2H), 3.10 (s, 3H). HRMS (ESI) calcd for C_20_H_20_FN_6_O_2_S^+^ [M + H]^+^: 427.1347, found: 427.1345.

#### 4.2.7. N^2^-(4-chlorobenzyl)-N^6^-(4-(methylsulfonyl)benzyl)-9H-purine-2,6-diamine (8)

This compound was purified by reverse phase HPLC using 0.1% formic acid in acetonitrile (A)/water (B). The gradient consisted of 10% A to 70% A in 35 min at 2 ml/min flow rate. *Rt* = 15.32 min. 41% yield. mp 138 °C. ^1^H NMR (600 MHz, DMSO-*d*_6_): *δ* 12.24 (brs, 1H), 8.13 (t, *J* = 17.4 Hz, 2H), 7.97 (brs, 1H), 7.79 (d, *J* = 11.5 Hz, 2H), 7.68 (s, 1H), 7.59 − 7.43 (m, 4H), 7.14 − 6.95 (m, 1H), 4.74 − 4.60 (m, 2H), 4.39 (d, *J* = 6.4 Hz, 2H), 3.15 (s, 3H). HRMS (ESI) calcd for C_20_H_20_ClN_6_O_2_S^+^ [M + H]^+^: 443.1052, found: 443.1066.

#### 4.2.8. N^6^-(4-(methylsulfonyl)benzyl)-N^2^-(4-nitrobenzyl)-9H-purine-2,6-diamine (9)

This compound was purified by reverse phase HPLC using 0.1% formic acid in acetonitrile (A)/water (B). The gradient consisted of 10% A to 70% A in 35 min at 2 ml/min flow rate. *Rt* = 12.97 min. 55% yield. mp 237 °C. ^1^H NMR (300 MHz, DMSO-*d*_6_): *δ* 12.24 (brs, 1H), 8.19 − 8.07 (m, 2H), 7.97 (brs, 1H), 7.79 (d, *J* = 6.0 Hz, 2H), 7.68 (s, 1H), 7.59 − 7.43 (m, 4H), 7.04 (brs, 1H), 4.65 (brs, 1H), 4.50 (s, 4H), 3.15 (s, 3H). HRMS (ESI) calcd for C_20_H_20_N_7_O_4_S^+^ [M + H]^+^: 454.1219, found: 454.1286.

#### 4.2.9. N^6^-(pyridin-3-ylmethyl)-N^2^-(3-(trifluoromethyl)benzyl)-9H-purine-2,6-diamine (10)

This compound was purified by reverse phase HPLC using 0.1% formic acid in acetonitrile (A)/water (B). The gradient consisted of 10% A to 60% A in 37 min at 1.8 ml/min flow rate. *Rt* = 10.35 min. 60% yield. mp 82 °C. ^1^H NMR (300 MHz, MeOH-*d*_4_): *δ* 8.53 − 8.33 (m, 2H), 7.86 − 763 (m, 3H.), 7.62 − 7.37 (m, 3H), 7.34 − 7.25 (m, 1H), 4.74 (s, 2H), 4.62 (s, 2H). HRMS (ESI) calcd for C_19_H_17_F_3_N_7_^+^ [M + H]^+^: 400.1492, found: 400.1474.

#### 4.2.10. N^2^-(4-fluorobenzyl)-N^6^-(pyridin-3-ylmethyl)-9H-purine-2,6-diamine (11)

This compound was purified by reverse phase HPLC using 0.1% formic acid in acetonitrile (A)/water (B). The gradient consisted of 0% A to 40% A in 25 min at 2 ml/min flow rate. *Rt* = 11.86 min. 42% yield. mp 261 °C. ^1^H NMR (300 MHz, MeOH-*d*_4_): *δ* 8.52 (d, *J* = 1.5 Hz, 1H), 8.39 (dd, *J* = 4.8 and 1.5 Hz, 1H), 7.75 (d, *J* = 7.8 Hz, 1H), 7.71 (s, 1H), 7.36 − 7.23 (m, 2H), 7.01 − 6.01 (m, 2H), 4.75 (s, 2H), 4.51 (s, 2H). HRMS (ESI) calcd for C_18_H_17_FN_7_^+^ [M + H]^+^: 350.1524, found: 350.1506.

#### 4.2.11. N^2^-(4-chlorobenzyl)-N^6^-(pyridin-3-ylmethyl)-9H-purine-2,6-diamine (12)

This compound was purified by reverse phase HPLC using 0.1% formic acid in acetonitrile (A)/water (B). The gradient consisted of 10% A to 90% A in 35 min at 1 ml/min flow rate. *Rt* = 6.93 min. 37% yield. mp 265 °C. ^1^H NMR (300 MHz, MeOH-*d*_4_): *δ* 8.54 − 8.36 (m, 2H), 7.78 − 7.68 (m, 2H), 7.36 − 7.29 (m, 1H), 7.28 − 7.18 (m, 4H), 4.75 (s, 2H), 4.53 (s, 2H). HRMS (ESI) calcd for C_18_H_17_ClN_7_^+^ [M + H]^+^: 366.1229, found: 366.1208.

#### 4.2.12. N^2^-(4-nitrobenzyl)-N^6^-(pyridin-3-ylmethyl)-9H-purine-2,6-diamine (13)

This compound was purified by reverse phase HPLC using 0.1% formic acid in acetonitrile (A)/water (B). The gradient consisted of 1% A to 65% A in 35 min; 65% to 1% A in 45 min at 1 ml/min flow rate. *Rt* = 6.26 min. 49% yield. mp 251 °C. ^1^H NMR (300 MHz, DMSO-*d*_6_): *δ* 8.45 (brs, 1H), 8.35 (s, 1H), 8.08 (d, *J* = 8.1 Hz, 2H), 7.70 − 7.53 (m, 2H), 7.48 (d, *J* = 8.4 Hz, 2H), 7.23 (s, 1H), 4.51 (s, 4H). HRMS (ESI) calcd for C_18_H_17_N_8_O_2_^+^ [M + H]^+^: 377.1469, found: 377.1434.

#### 4.2.13. N^6^-((1H-imidazol-2-yl)methyl)-N^2^-(3-(trifluoromethyl)benzyl)-9H-purine-2,6-diamine (14)

This compound was purified by reverse phase HPLC using 0.1% formic acid in acetonitrile (A)/water (B). The gradient consisted of 0% A to 30% A in 45 min at 2 ml/min flow rate. *Rt* = 18.06 min. 42% yield. mp 236 °C. ^1^H NMR (300 MHz, MeOH-*d*_4_): *δ* 8.37 (s, 1H), 7.76 (s, 1H), 7.62 − 7.42 (m, 4H), 7.09 (s, 2H), 4.84 (s, 2H), 4.61 (s, 2H). HRMS (ESI) calcd for C_17_H_16_F_3_N_8_^+^ [M + H]^+^: 389.1445, found: 389.1446.

#### 4.2.14. N^6^-((1H-imidazol-2-yl)methyl)-N^2^-(4-fluorobenzyl)-9H-purine-2,6-diamine (15)

This compound was purified by reverse phase HPLC using 0.1% formic acid in acetonitrile (A)/water (B). The gradient consisted of 1% A to 35% A in 35 min at 1 ml/min flow rate. *Rt* = 13.18 min. 33% yield. mp 236 °C. ^1^H NMR (300 MHz, MeOH-*d*_4_): *δ* 8.32 (brs, 1H), 7.77 (s, 1H), 7.32 − 7.24 (m, 2H), 7.19 (m, 2H), 7.03 − 6.94 (m, 2H), 4.87 (s, 2H), 4.48 (s, 2H). HRMS (ESI) calcd for C_16_H_16_FN_8_^+^ [M + H]^+^: 339.1477, found: 339.1492.

#### 4.2.15. N^6^-((1H-imidazol-2-yl)methyl)-N^2^-(4-chlorobenzyl)-9H-purine-2,6-diamine (16)

This compound was purified by reverse phase HPLC using 0.1% formic acid in acetonitrile (A)/water (B). The gradient consisted of 0% A to 30% A in 40 min at 2 ml/min flow rate. *Rt* = 15.49 min. 39% yield. mp 155 °C. ^1^H NMR (300 MHz, MeOH-*d*_4_): *δ* 8.31 (brs, 1H), 7.77 (s, 1H), 7.47 (s, 1H), 7.24 (s, 4H), 7.19 (s, 2H), 4.87 (s, 2H), 4.48 (s, 2H). HRMS (ESI) calcd for C_16_H_16_ClN_8_^+^ [M + H]^+^: 355.1181, found: 355.1170.

#### 4.2.16. N^6^-((1H-benzo[d]imidazol-2-yl)methyl)-N^2^-(3-(trifluoromethyl)benzyl)-9H-purine-2,6-diamine (17)

This compound was purified by reverse phase HPLC using 0.1% formic acid in acetonitrile (A)/water (B). The gradient consisted of 10% A to 50% A in 30 min at 2 ml/min flow rate. *Rt* = 11.97 min. 67% yield. mp 289 °C. ^1^H NMR (300 MHz, MeOH-*d*_4_): *δ* 8.21 (s, 1H), 7.77 (s, 1H), 7.56 − 7.41 (m, 3H), 7.35 − 7.21 (m, 4H), 7.09 − 7.15 (m, 1H), 5.02 (s, 2H), 4.52 (s, 2H). HRMS (ESI) calcd for C_21_H_18_F_3_N_8_^+^ [M + H]^+^: 439.1601, found: 439.1634.

#### 4.2.17. N^6^-((1H-benzo[d]imidazol-2-yl)methyl)-N^2^-(4-fluorobenzyl)-9H-purine-2,6-diamine (18)

This compound was purified by reverse phase HPLC using 0.1% formic acid in acetonitrile (A)/water (B). The gradient consisted of 10% A to 50% A in 30 min at 2 ml/min flow rate. *Rt* = 7.31 min. 46% yield. mp 287 °C. ^1^H NMR (300 MHz, MeOH-*d*_4_): *δ* 8.17 (brs, 1H), 7.81 (s, 1H), 7.56 − 7.46 (m, 2H), 7.37 − 7.25 (m, 2H), 7.13 − 7.01 (m, 2H), 6.74 − 6.61 (m, 1H), 5.05 (s, 2H), 4.42 (s, 2H). HRMS (ESI) calcd for C_20_H_17_FN_8_^+^ [M + H]^+^: 389.1633, found: 389.1606.

#### 4.2.18. N^6^-((1H-benzo[d]imidazol-2-yl)methyl)-N^2^-(4-chlorobenzyl)-9H-purine-2,6-diamine (19)

This compound was purified by reverse phase HPLC using 0.1% formic acid in acetonitrile (A)/water (B). The gradient consisted of 10% A to 50% A in 30 min at 2 ml/min flow rate. *Rt* = 12.85 min. 40% yield. mp 160 °C. ^1^H NMR (300 MHz, MeOH-*d*_4_): *δ* 7.84 (s, 1H), 7.57 − 7.46 (m, 2H), 7.40 − 7.31 (m, 2H), 7.07 − 6.86 (m, 4H), 5.07 (s, 2H), 4.42 (s, 2H). HRMS (ESI) calcd for C_20_H_18_ClN_8_^+^ [M + H]^+^: 405.1338, found: 405.1358.

#### 4.2.19. N^6^-((1H-benzo[d]imidazol-2-yl)methyl)-N^2^-(4-nitrobenzyl)-9H-purine-2,6-diamine (20)

This compound was purified by reverse phase HPLC using 0.1% formic acid in acetonitrile (A)/water (B). The gradient consisted of 10% A to 40% A in 30 min at 2 ml/min flow rate. *Rt* = 6.47 min. 55% yield. mp 153 °C. ^1^H NMR (300 MHz, MeOH-*d*_4_): *δ* 8.16 (s, 1H), 7.82 (s, 1H), 7.80 − 7.68 (m, 2H), 7.47 − 7.36 (m, 2H), 7.28 − 7.15 (m, 4H), 4.98 (s, 2H), 4.56 (s, 2H). HRMS (ESI) calcd for C_20_H_17_N_9_O_2_^+^ [M + H]^+^: 416.1578, found: 416.1585.

### 4.3. LC-MS analysis of CYP450s metabolism

#### 4.3.1. Reagents

Phenacetin, coumarin, bupropion, tolbutamide, 4-hydroxytolbutamide, dextromethorphan, chlorzoxazone, 6-hydroxychlorzoxazone, testosterone, carbamazepine, the reduced form of β-nicotinamide adenine dinucleotide phosphate (NADPH), 4-methyl umbelliferone, were purchased from Sigma-Aldrich (St, Louis, MO). Amodiaquine, *S*-mephenytoin and pooled HLMs (BD UltraPool HLM 150) were purchased from Corning (Woburn, MA). The manufacturer-supplied information on the HLMs regarding protein concentration, CYP content and enzyme activity. Midazolam was purchased from Bukwang Pharma Co. (Seoul, Republic of Korea). All other reagents and chemicals were of analytical or high-performance liquid chromatography (HPLC) grade.

### 4.4. Direct CYP inhibition assay

Reactions were incubated in eight-well strips placed in an 8 × 12 racks (1.2 ml; VWR, Emeryville, CA). The incubation mixtures (final volume, 200 μL) contained 0.2 mg/mL HLMs, 0.1 M phosphate buffer (pH 7.4), 1 mM NADPH, and various CYP isoform-specific substrate cocktail sets (A set: phenacetin, coumarin, amodiaquine, *S*-mephenytoin, dextromethorphan, and midazolam; B set: bupropion, tolbutamide, chlorzoxazone, and testosterone). The substrates were used at concentrations approximately equal to their respective K_m_ values: 50 μM for phenacetin, 5 μM for coumarin, 50 μM for bupropion, 2 μM for amodiaquine, 100 μM for tolbutamide, 100 μM for *S*-mephenytoin, 5 μM for dextromethorphan, 50 μM for chlorzoxazone, 5 μM for midazolam, and 50 μM for testosterone^21^^,^^22^. Substrates were dissolved and serially diluted with acetonitrile to the required concentration in all experiments. The final acetonitrile concentration of the cocktail incubations was 0.2% in set A or 0.25% in set B. After a 5-min pre-incubation at 37 °C with 0 to 50 μM TNP, the reactions were initiated by adding 1 mM NADPH. The reactions were incubated at 37 °C in a shaking water bath. The reactions were terminated by placing the incubation tubes on ice, adding 200 μL of cold acetonitrile containing carbamazepine (100 nM) and 4-methylumbelliferone (500 nM) as an internal standard after 10 min, and agitating with a vortex mixer before centrifugation. The samples for each enzyme assay were centrifuged at 3,000 × g for 20 min at 4 °C, and supernatants of the individual reaction samples and pooled cocktail incubation samples (A set: B set, 1:1) were analysed using LC-MS/MS.

### 4.5. Analytical method

Sample injection volume was 10 μL, and separation was performed on an Atlantis™ dC18 column (2.1 × 50 mm i.d., 3 μm; Waters, Milford, MA, USA) and Luna™5u C18 column (2.0 × 30 mm i.d.; Phenomenex, Torrance, CA, USA) with a SecurityGuard™ C18 guard column (2.0 × 4.0 mm i.d.; Phenomenex, Torrance, CA, USA) maintained at 30 °C. The column was pre-equilibrated in 100% v/v solvent A (deionized water containing 0.1% v/v formic acid)/0% v/v solvent B (acetonitrile containing 0.1% v/v formic acid) at a flow rate of 0.4 ml/min. The optimized LC elution conditions were: 0.0–1.0 min, 0% B; 1.0–1.1 min, 0– 45% B; 1.1–4.0 min, 50% B; 4.0–4.1 min, 50–95% B; 4.1–6.0 min, 95–95% B; 6.0–6.01 min, 95–0% B and 6.0–7.0 min, 0% B. The liquid chromatography-electrospray ionisation-tandem mass spectrometry (LC-ESI/MS/MS) system consisted of a Shimadzu 20AD-XR HPLC system (Shimadzu, Kyoto, Japan) and an API 3200 Q-TRAP LC-MS/MS system equipped with a Turbo V™ Ion Spray source (Applied Biosystems, Foster City, CA, USA) operated in the negative or positive ion mode. Quadrupoles Q1 and Q3 were set on unit resolution. The samples were analysed via multiple reaction monitoring (MRM) and enhanced product ion (EPI) scan mode. The turbo ion spray interface was operated in the positive ion mode at 5,500 V. The operating conditions were determined as follows: ion source temperature, 600 °C; nebulising gas flow, 50 L/min; auxiliary gas flow, 4.0 L/min; curtain gas flow, 20 L/min; collision gas (nitrogen) pressure 3.6 × 10^−5 ^Torr. Nitrogen gas was used for CUR, CAD, and NEB. The MRM transitions, collision energies and retention time were determined for each metabolite. The samples were analysed via MRM as described in our previous study^21^. The scan dwell time was set at 0.08 s for every channel. Data acquisition and analysis was performed using the Analyst™ software (version 1.5.2; Applied Biosystems, Foster City, CA, USA).

### 4.6. Spectral binding titrations analysis of CYP3A4

Purified CYP3A4 enzymes were diluted to 1 µM in 100 mM potassium phosphate buffer (pH 7.4) and then divided into two glass cuvettes. The spectra (350–500 nm) were recorded while subsequently adding TNP and its analogs using CARY 100 Varian spectrophotometer (Palo Alto, CA, USA). The concentration of ligands was plotted against the difference in absorbance between the wavelengths maximum (430 nm) and minimum (385 nm). The binding titration was analysed using GraphPad Prism software (GraphPad, Inc., La Jolla, CA, USA).

### 4.7. *In vitro* CYP450 activity assay

The activity of CYP3A4 was measured using the Vivid^®^ CYP450 Blue screening kit (Cat No. P2858 for 3A4, Invitrogen). The reaction solutions contained various concentrations of TNP and TNP derivatives, 5 nM each Vivid^®^ CYP450 BACULOSOMES, 10 μM CYP450 substrate Vivid^®^ BOMCC, 30 μM NADP, 1X Vivid^®^ CYP450 Reaction buffer I, 1X Vivid^®^ Regeneration. The system consisted of 100 μL of the final volume. The CYP450 substrate Vivid BOMCC and positive control inhibitor (ketoconazole), were dissolved in acetonitrile. TNP and derivatives were dissolved in DMSO, and the final concentration of acetonitrile and DMSO in the final reaction solution was 0.1%, respectively. The mixture containing test drugs, Vivid^®^ CYP450 BACULOSOMES reagent, and Vivid^®^ Regeneration system (Total 90 μL) was dispensed into a 96-well black plate and incubated at room temperature for 20 min. After adding 10 μL of Vivid^®^ CYP450 reaction buffer (100 μM NADP, 10 μM Vivid^®^ BOMCC), the mixture was incubated for 10 min at room temperature. The final concentrations of TNP and ketoconazole used in the reaction were 50, 100, 250, 500, 1000 nM, and the comparison of the inhibitory effects of TNP and derivatives was performed at a final concentration of 100 nM. The fluorescence value of the final reactant was measured for 10 min at 1-min intervals at excitation 405 nm/emission 460 nm using the kinetic mode of the Mithras LB940 plate reader from Berthold. The CYP450 3A4 inhibitory efficiency (%) of the test drug was calculated by comparing the reaction rates of the test compounds with the reaction rate of the positive control group (Ketoconazole) and the negative control group (0.1% Acetonitrile, 0.1% DMSO) using the measured fluorescence values.

Inhibitory potential of compound **9** against CYP1A2 and CYP2E1 was also measured using Vivid^®^ CYP450 Blue screening kit (Cat No. P2863 and P3021) obtained from Invitrogen according to the manufacturer's instructions. The reaction solutions contained various concentrations of the test compound, 5–20 nM Vivid^®^ CYP450 BACULOSOMES, 3–10 μM CYP450 substrate Vivid^®^ EOMCC, 30 μM NADP, 1X Vivid^®^ CYP450 Reaction buffer I, 1X Vivid^®^ Regeneration. respectively. the final screening concentration of DMSO in the final reaction solution was 0.1%. The fluorescence value of the final reactant was measured for 10–60 min at 1-min intervals at excitation 405 nm/emission 460 nm using the kinetic mode of the Mithras LB940 plate reader from Berthold. The final activity of CYP1A2 and CYP2E1 was calculated by comparing with the DMSO control.

### 4.8. Recombinant FLAG-IP6K2 protein purification

Full-length human IP6K2 cDNA was subcloned into pFASTBAC1 plasmid (Gibco) with an N-terminal FLAG epitope sequence, and baculovirus was generated according to the manufacturer’s instructions. Sf9 insect cells were infected with baculovirus and incubated for 72 h. The cells were resuspended in lysis buffer [20 mM Tris–HCl (pH 7.9), 500 mM NaCl, 4 mM MgCl_2_, 0.4 mM EDTA, 2 mM DTT, 20% glycerol, 1 mM PMSF, and protease inhibitor cocktail (Roche)] and then disrupted with a Dounce homogeniser (pestle A, 3 series of 10 strokes with 10 min interval). Clarified extracts were adjusted to 300 mM NaCl by adding dilution buffer [20 mM Tris–HCl (pH 7.9) and 10% glycerol], supplemented with final 0.1% NP-40, and then subjected to affinity purification on M2 agarose (Sigma). After extensive washing with wash buffer [20 mM Tris–HCl (pH 7.9), 150 mM NaCl, 2 mM MgCl_2_, 0.2 mM EDTA, 1 mM DTT, 15% glycerol, 1 mM PMSF, and 0.1% NP-40], FLAG-IP6K2 protein was eluted with elution buffer (wash buffer containing 0.25 mg/mL FLAG-peptide and protease inhibitor cocktail) and stored at −80 °C.

### 4.9. IP6K2 Inhibition assay (ADP Glo kinase assay)

IP6K2 activity was measured using ADP Glo assay kit (Promega). All kinase assays were performed in low volume 384-well white plates (784075, Greiner-bioone) with a final volume of 5 μL. The reaction buffer contained 2 ng Flag-hIP6K2, 10 μM IP6, and 10 μM ATP in kinase buffer (50 mM Tris, 10 mM MgCl_2_, 2.5 mM DTT, 0.02% Triton-X-100, pH 6.8)^26^. For dose-dependent inhibition assay, 8 serial 2-fold dilutions of test compounds were prepared in DMSO. The final DMSO concentration was 5% in each reaction. The test compounds (5X, 25% DMSO in 1X kinase buffer) were dispensed to each well, and 2.5 X IP6K2 in kinase buffer were added. After 15 min incubation at room temperature, 2.5 X concentrations of IP6 and ATP were added to the plate. The reaction plate was incubated in a 37 °C incubator for 30 min. Next, the reactions were quenched with 5 μL of ADP Glo reagent for 40 min to deplete the remaining ATP. To convert the remaining ADP to ATP, 10 μl of detection reagent were added and incubated for 30–60 min. The luminescence of the final reactants was measured using the Mithras LB940 plate reader from Berthold. Samples were run in triplicate and the activity (%) was calculated using the following equations; Activity (%) = 100 × (*μ*_experimental_–*μ*_negative_)/(*μ*_positive_–*μ*_negative_). Negative control was DSMO-treated reaction. IC_50_ value was estimated using a logistic regression model with R package.

### 4.10. Extractions and quantification of intracellular inositol phosphates by HPLC

For HPLC analysis, 2 × 10^5^ cells/60 mm dish of HCT116 were treated with 75 μCi [^3^H] myo-inositol (NET1177001MC, PerkinElmer). After 3 days, soluble inositol polyphosphates from HCT116 (human colorectal cancer cell line) were extracted and analysed as previously described^27^. Intracellular inositol phosphates were extracted with acid extraction buffer (1 M HClO_4_, 3 mM EDTA, and 0.1 mg/ml1 IP6), and neutralised with neutralisation buffer (1 M K_2_CO_3_ and 3 mM EDTA). The lysates were centrifuged for 10 min, and the soluble fraction was resolved by HPLC as described earlier^27^. The lipid pellet was lysed with 0.1% Triton X-100 in NaOH overnight. Each fraction was mixed with Ultima-Flo AP liquid scintillation cocktail (6013599, PerkinElmer), and radioactivity was counted in a scintillation counter. Quantity of inositol phosphates was presented as total counts/min (CPM) normalised by total lipid contents. The test compounds including TNP were dissolved in DMSO, and the final concentration of DMSO in cells was 0.5%.

### 4.12. AKT activation in HCT116 cells

For Immunoblotting analysis, HCT116 cells were treated with 0.5% DMSO, TNP and analogs for 4 h, and then cellular proteins were prepared with lysis buffer (25 mM Tris-HCl pH 7.4, 150 mM NaCl, 1 mM EDTA, 1% NP-40, 10 mM NaF, 50 mM Na_4_P_2_O_7_, 1 X Protein inhibitor cocktail (Roche)). Total proteins were quantified by bicinchoninic acid assay (23225, Thermo Fisher Scientific). Total 20 μg proteins were resolved in 8% SDS–PAGE, and then Akt phosphorylation was probed with primary antibodies against, phospho–Akt (Thr308) (4056) and Akt (9272) (Cell Signalling) and HRP-conjugated secondary antibody. The relative band intensities of Akt phosphorylation to total Akt were quantified with Image J software.

### 4.12. Molecular docking studies

*Ligand preparation and optimisation:* All ligands were generated as 2D and 3D structures by *ChemDraw Ultra* (ver. 12.0.2) and *Chem3D Pro* (ver. 11.0.1), respectively. Ligand preparation and optimisation were followed by *‘Sanitize’* preparation protocol in *SYBYL-X 2.1.1* (Tripos Inc., St Louis) to clean up the structures involving filling valences, standardising, removing duplicates, and producing only one molecule per input structure. The group of ligands was saved as *.sdf* file.

*Protein preparation:* IP6K2A homology model was built based on the crystal structure of *Eh*IP6KA in complex with ATP and Ins(1,4,5,)P_3_ (PDB ID: 4O4D; sequence identity: 31.02). The protein sequence of IP6K2A was obtained from NCBI protein database (http://ncbi.nlm.nih.gov/protein) as FASTA format. The homology model of IP6K2A was prepared using SWISS-MODEL (http://swissmodel.expasy.org). *SYBYL-X 2.1.1* program was employed for protein preparation including conflicted side chains of amino acid residues fixation. Hydrogen atoms were added under the application of *TRIPOS* Force Field setting for both proteins. The minimisation process was performed by *POWELL* method, and *the* initial optimisation option was set to *Simplex.* Termination gradient and max iteration were set 0.05 *kcal/(mol* *Å)* and 100 times, respectively.

*Docking and scoring function studies:* The docking studies of all prepared ligands were performed by *Surflex-Dock GeomX* module in *SYBYL-X 2.1.1*. *Surflex-Dock protomol* set to ‘*Residues*’ method with selected amino acids (Leu206, Glu207, Asn208, Leu209, Thr210, Val 218, Leu219, Asp220, Leu221, Lys222; radius setting: 2.2; Those amino acids were selected based on the active site of *Eh*IP6KA.) was used to guide docking site for IP6K2A homology model. Two factors related to a generation of Protomol are *Bloat(Å)* and *Threshold* were set to 0.5 and 0, respectively. Other parameters were applied with its default settings in all runs.

## Supplementary Material

Supplemental Material
