# Supplementary material for: TNP and its analogs: Modulation of IP6K and CYP3A4 inhibition
Source: J Enzyme Inhib Med Chem. 2021 Dec 11;37(1):269–79. doi: 10.1080/14756366.2021.2000404 (PMC8667942; doi:10.1080/14756366.2021.2000404)

Supporting Information for

TNP and its analogs: Modulation of IP6K and CYP3A4 inhibition

Seulgi Lee,[†,#] Bernie Byeonghoon Park,[‡,#] Hongmok Kwon,[‡] Vitchan Kim,[⊥] Jang Su Jeon,[§] Rowoon Lee,[⊥] Milan Subedi,[‡] Taehyeong Lim,[‡] Hyunsoo Ha,[‡] Dongju An,[†] Jaehoon Kim,[†] Donghak Kim,[⊥] Sang Kyum Kim,[§,*] Seyun Kim,[†,¶,*] Youngjoo Byun[†,◊,*]

[†] Department of Biological Sciences, KAIST, Daejeon 34141, South Korea

[‡] College of Pharmacy, Korea University, 2511 Sejong-ro, Sejong 30019, South Korea

[⊥] Department of Biological Sciences, Konkuk University, Seoul 05029, South Korea

[§] College of Pharmacy, Chungnam National University, 99 Daehak-ro, Yuseong-gu, Daejeon 34134

[¶] KAIST Institute for the BioCentury, KAIST, Daejeon 34141, Korea

[◊] Biomedical Research Center, Korea University Guro Hospital, 148 Gurodong-ro, Guro-gu, Seoul 08308, Republic of Korea

Table of contents

**Content**	**page**
Figure 1. The effects of TNP on activities of CYP isoforms	S5
Figure 2. IP levels after treatment of TNP and three compounds	S6
Figure 3. The effects of compound 9 on CYP1A2 and CYP2E1 activities	S6
Figure 4. The docked pose of TNP in IP6K homology model	S7
Figure 5. The superimposed poses of TNP and compound 9	S7
Figure 6. The best-docked pose of compound 9 with CYP3A4	S8
Figure 7. The best-docked pose of TNP with CYP3A4	S8
^{1}H NMR spectra of compound **2**	**S9**
HRMS spectra of compound **2**	**S10**
^{1}H NMR spectra of compound **3**	**S11**
HRMS spectra of compound **3**	**S**12
^{1}H NMR spectra of compound **4**	**S**13
HRMS spectra of compound **4**	**S**14
^{1}H NMR spectra of compound **5**	**S**15
HRMS spectra of compound **5**	**S**16
^{1}H NMR spectra of compound **6**	**S**17
HRMS spectra of compound **6**	**S**18

Content **page**

Binding spectra of compound **6** in CYP3A4 S19

^{1}H NMR spectra of compound **7** S20

HRMS spectra of compound **7** S21

Binding spectra of compound **7** in CYP3A4 S22

^{1}H NMR spectra of compound **8** S23

HRMS spectra of compound **8** S24

Binding spectra of compound **8** in CYP3A4 S25

^{1}H NMR spectra of compound **9** S26

HRMS spectra of compound **9** S27

^{1}H NMR spectra of compound **10** S28

Binding spectra of compound **10** in CYP3A4 S29

HRMS spectra of compound **10** S30

^{1}H NMR spectra of compound **11** S31

HRMS spectra of compound **11** S32

Binding spectra of compound **11** in CYP3A4 S33

^{1}H NMR spectra of compound **12** S34

HRMS spectra of compound **12** S35

Binding spectra of compound **12** in CYP3A4 S36

**Content**	**page**
^{1}H NMR spectra of compound **13**	**S**37
HRMS spectra of compound **13**	**S**38
Binding spectra of compound **13** in CYP3A4	S39
^{1}H NMR spectra of compound **14**	**S**40
HRMS spectra of compound **14**	**S**41
Binding spectra of compound **14** in CYP3A4	S42
^{1}H NMR spectra of compound **15**	**S**43
HRMS spectra of compound **15**	**S**44
Binding spectra of compound **15** in CYP3A4	S45
^{1}H NMR spectra of compound **16**	**S**46
HRMS spectra of compound **16**	**S**47
Binding spectra of compound **16** in CYP3A4	S48
^{1}H NMR spectra of compound **17**	**S**49
HRMS spectra of compound **17**	**S**50
Binding spectra of compound 1**7** in CYP3A4	S51
^{1}H NMR spectra of compound **18**	**S**52
HRMS spectra of compound **18**	**S**53
Binding spectra of compound **18** in CYP3A4	S54

Content **page**

^{1}H NMR spectra of compound **19** **S**55

HRMS spectra of compound **19** **S**56

Binding spectra of compound **19** in CYP3A4 S57

^{1}H NMR spectra of compound **20** **S**58

HRMS spectra of compound **20** **S**59

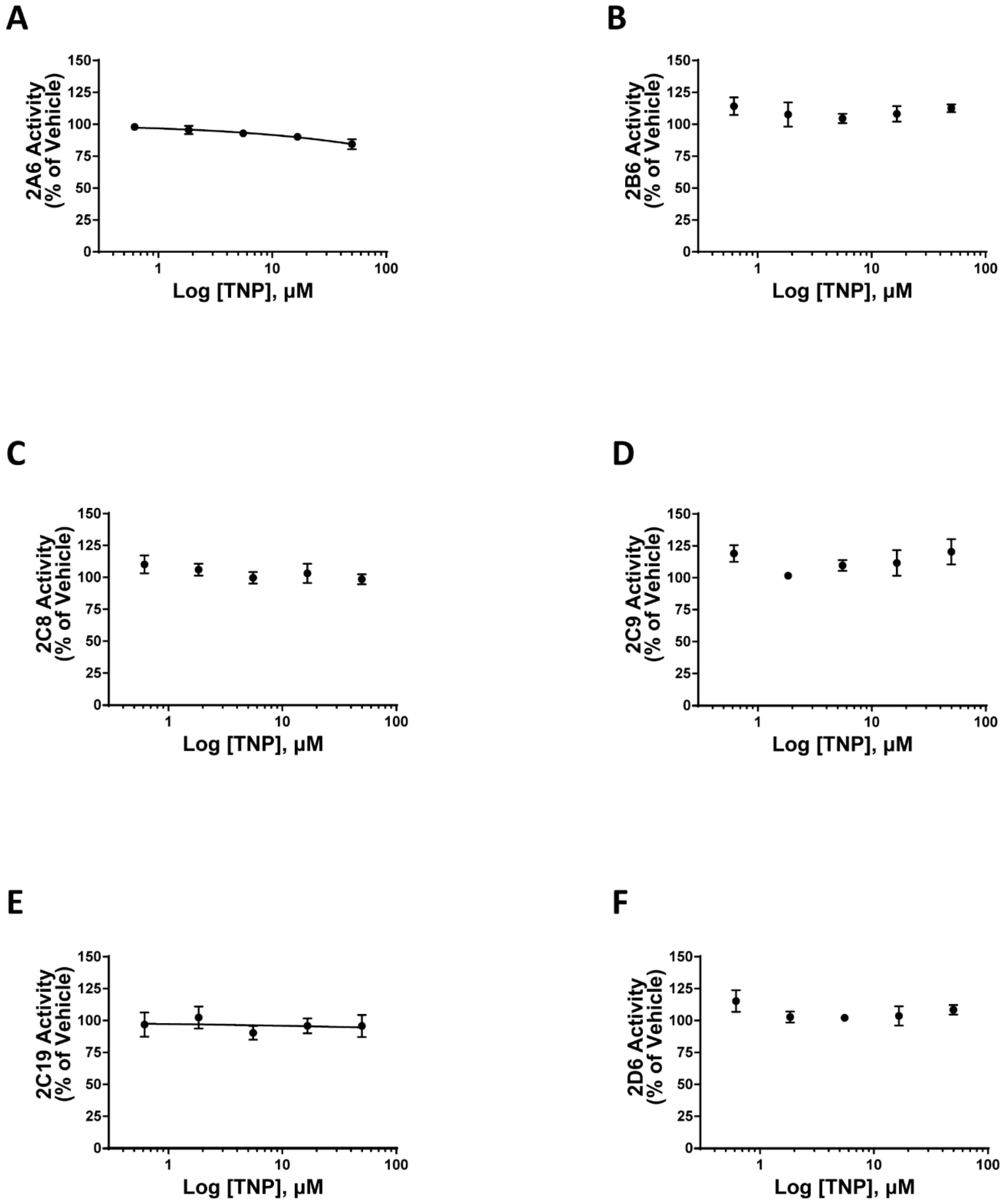

Supplementary Figure 1. The effects of TNP on activities of CYP isoforms in human liver microsome. (A-F) Screening the activities of 6 types of human liver microsomal CYP450s. The CYP450 metabolic capacities were measured by LC-MS/MS against 6 isoform-specific substrates (coumarin for 2A6, bupropion for 2B6, amodiaquine for 2C8, tolbutamide for 2C9, S-mephenytoin for 2C19, and dextromethorphan for 2D6). Values in all graphs are represented as mean ± SEM.

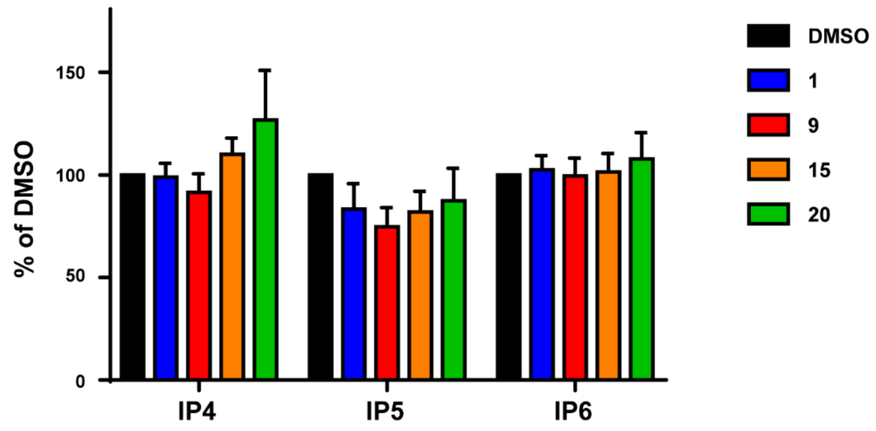

Supplementary Figure 2. IP levels after treatment of TNP and three compounds in HCT116 cells. Cellular IP4, IP5, and IP6 levels in HCT116 were analyzed by HPLC after 4 h treatment with DMSO, TNP (1) 10 μM, and the selected compounds (**9**, **15**, and **20**) 50 μM. Values in a graph are presented as mean ± SEM.

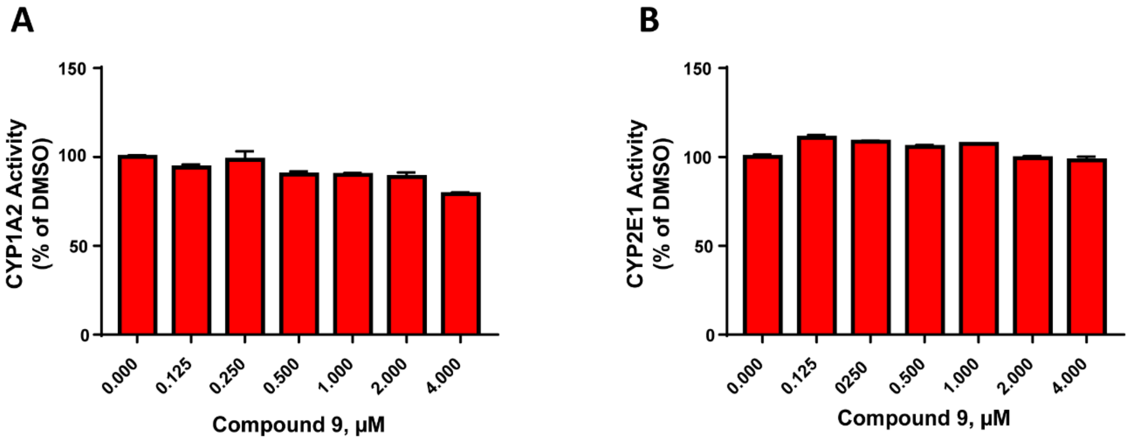

Supplementary Figure 3. The effects of compound 9 on CYP1A2 and CYP2E1 activities. In vitro analysis of recombinant microsomal (A) CYP1A2 and (B) CYP2E1 against compound **9** treatment with designated concentrations on graphs. Values in all graphs are presented as mean ± SEM.

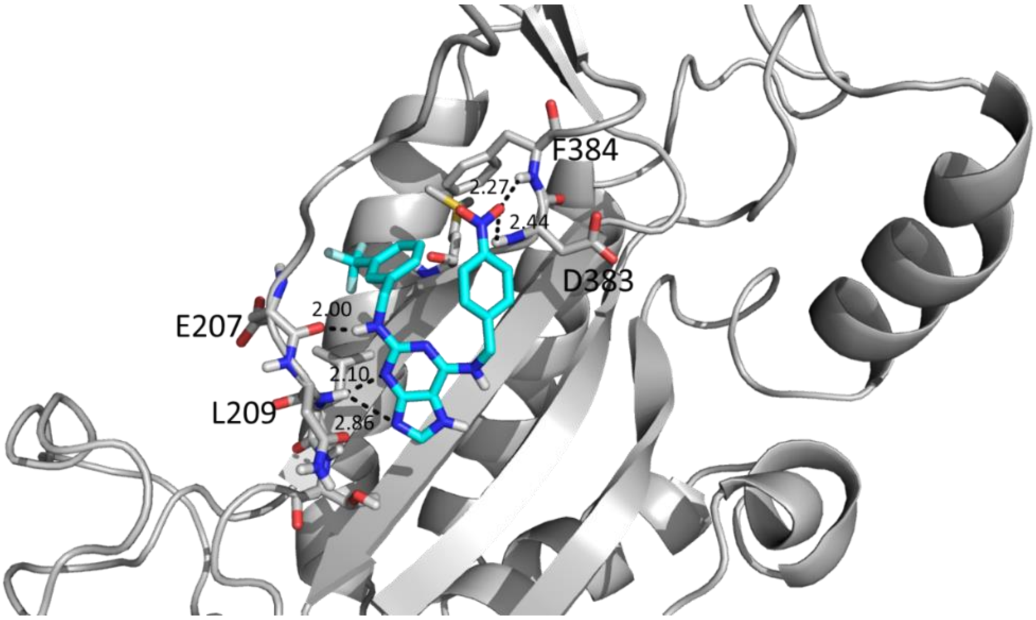

Supplementary Figure 4. The docked pose of TNP in IP6K homology model

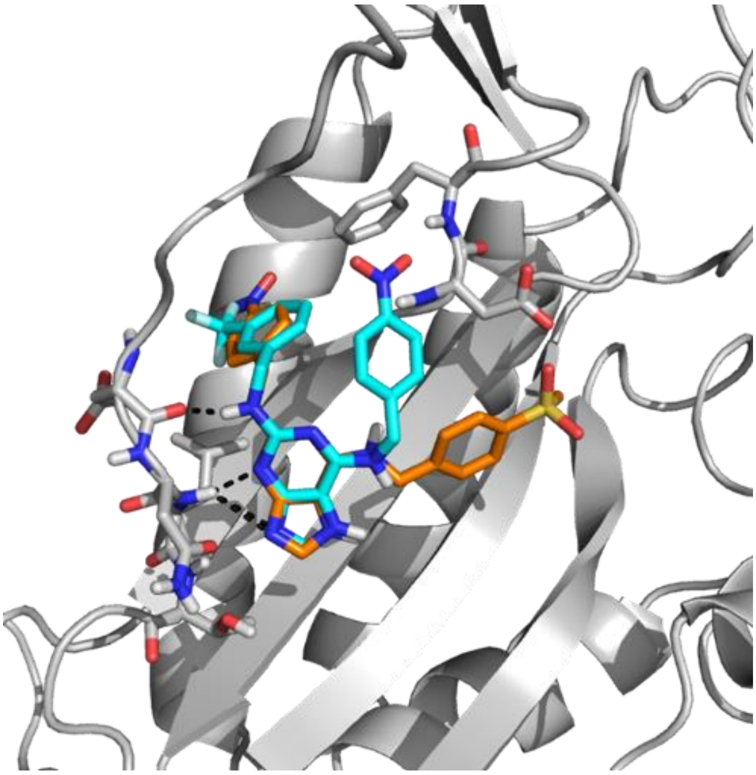

Supplementary Figure 5. The superimposed poses of TNP and compound 9 in the IP6K homology model. TNP and compound **9** were shown in light blue and yellow orange, respectively.

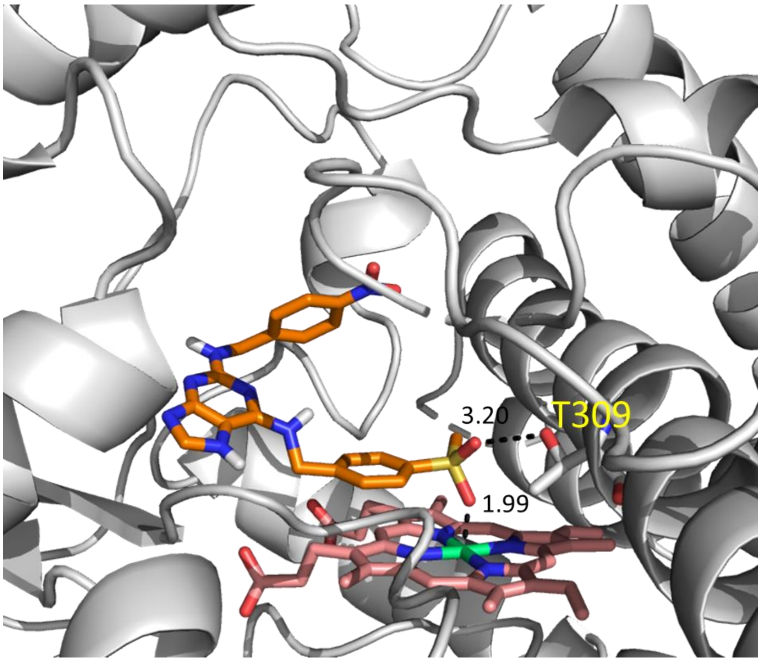

Supplementary Figure 6. The best-docked pose of compound 9 with CYP3A4 crystal structure (PDB ID: 3NXU)

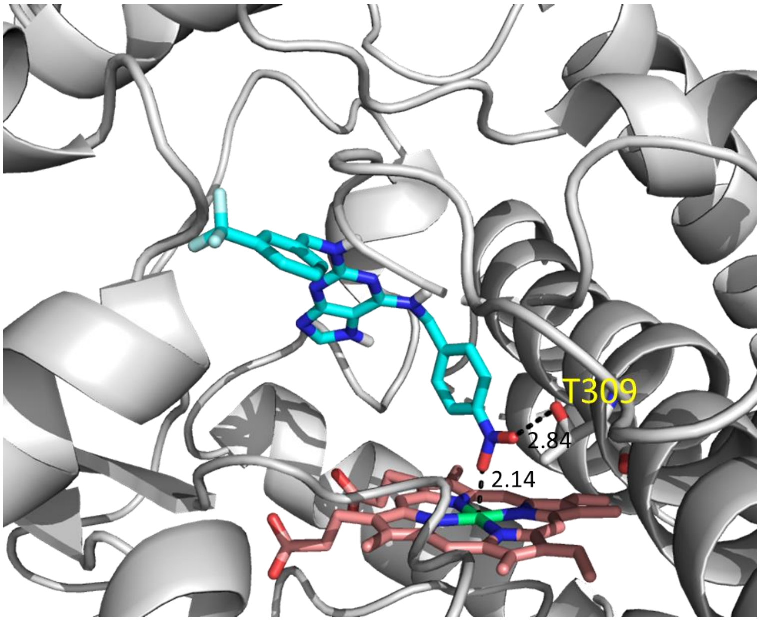

Supplementary Figure 7. The best-docked pose of TNP with CYP3A4 crystal structure (PDB ID: 3NXU)

^{1}H NMR spectra of compound **2** measured in DMSO-d_6 at 300 MHz

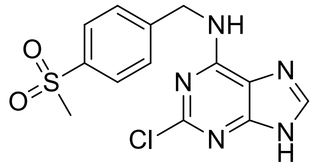

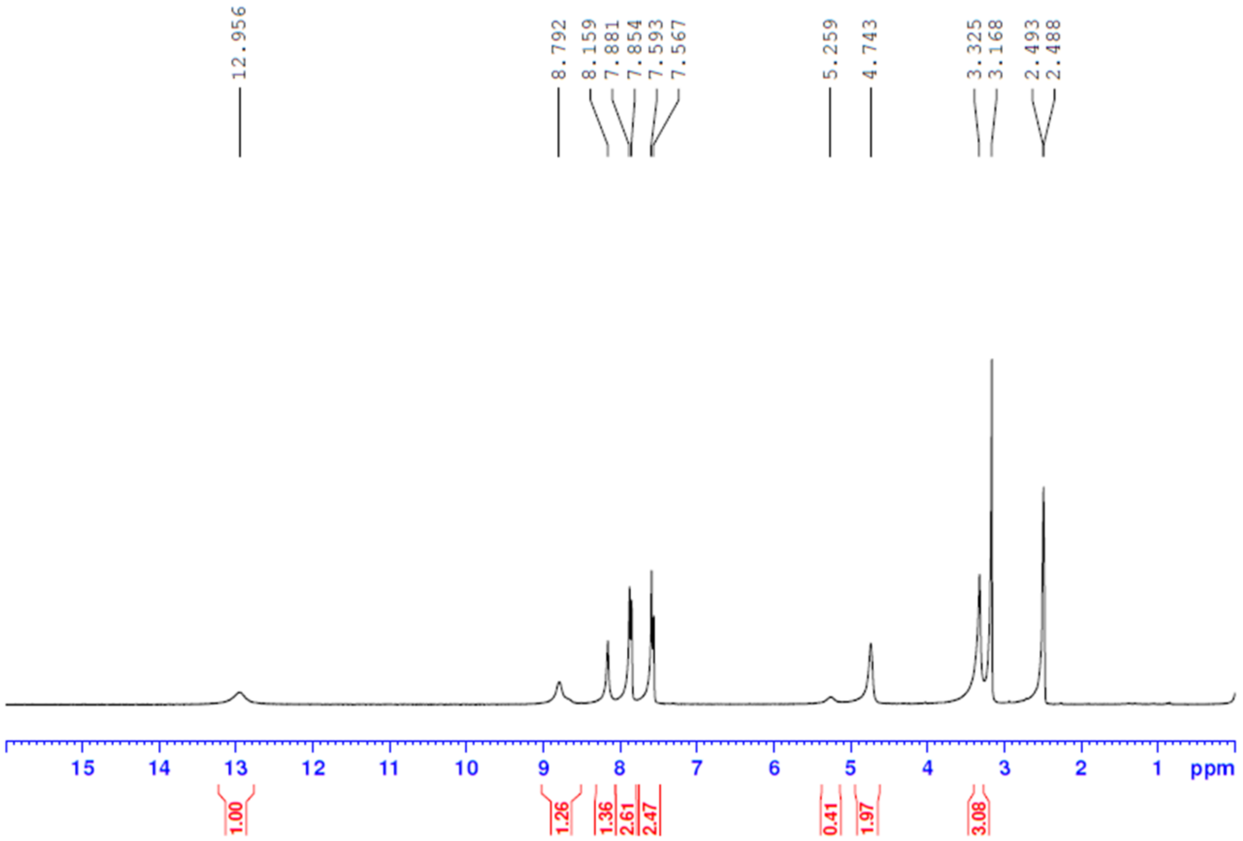

HRMS spectra of compound **2**

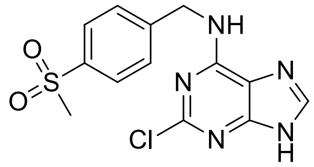

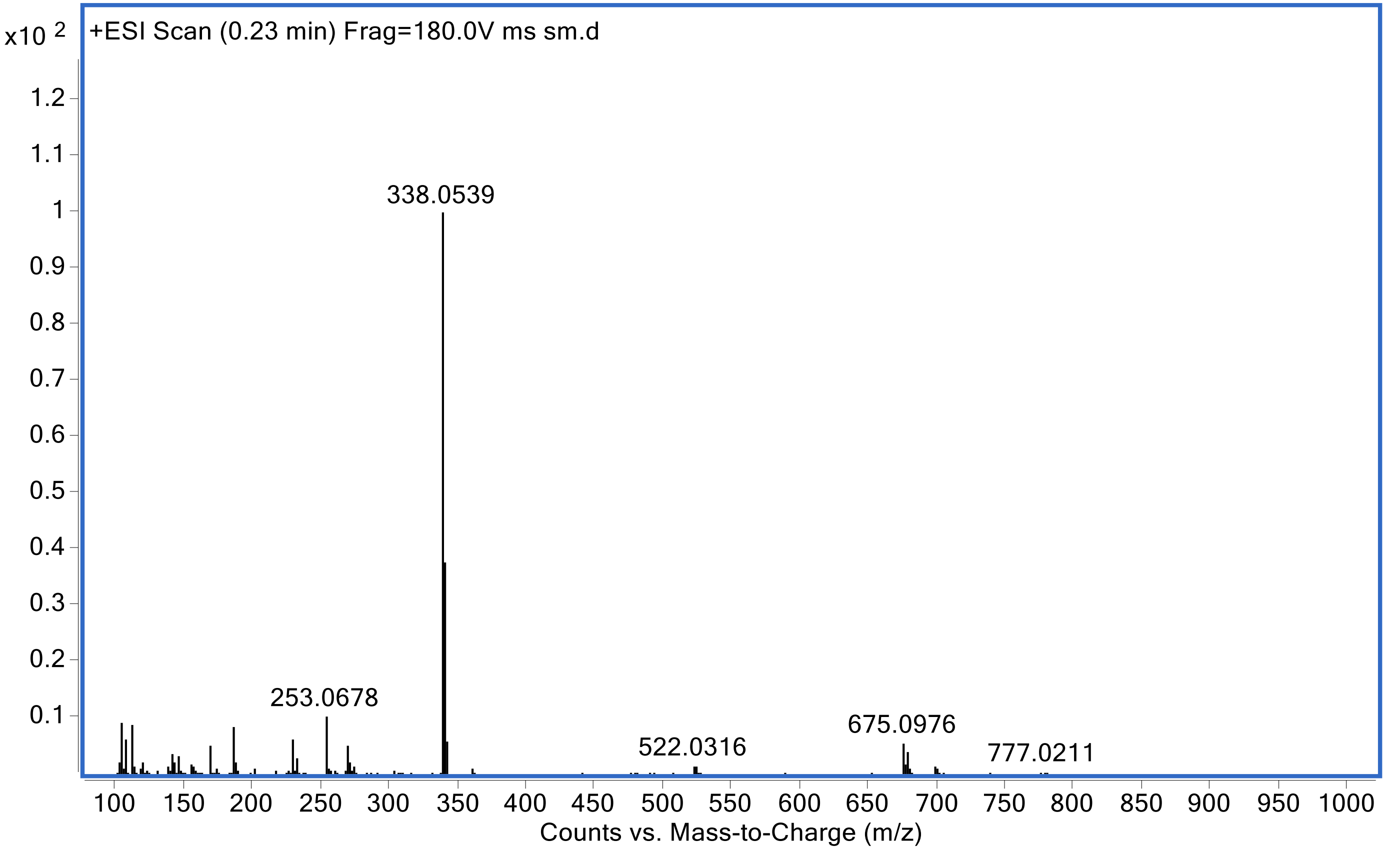

^{1}H NMR spectra of compound **3** measured in DMSO-d_6 at 300 MHz

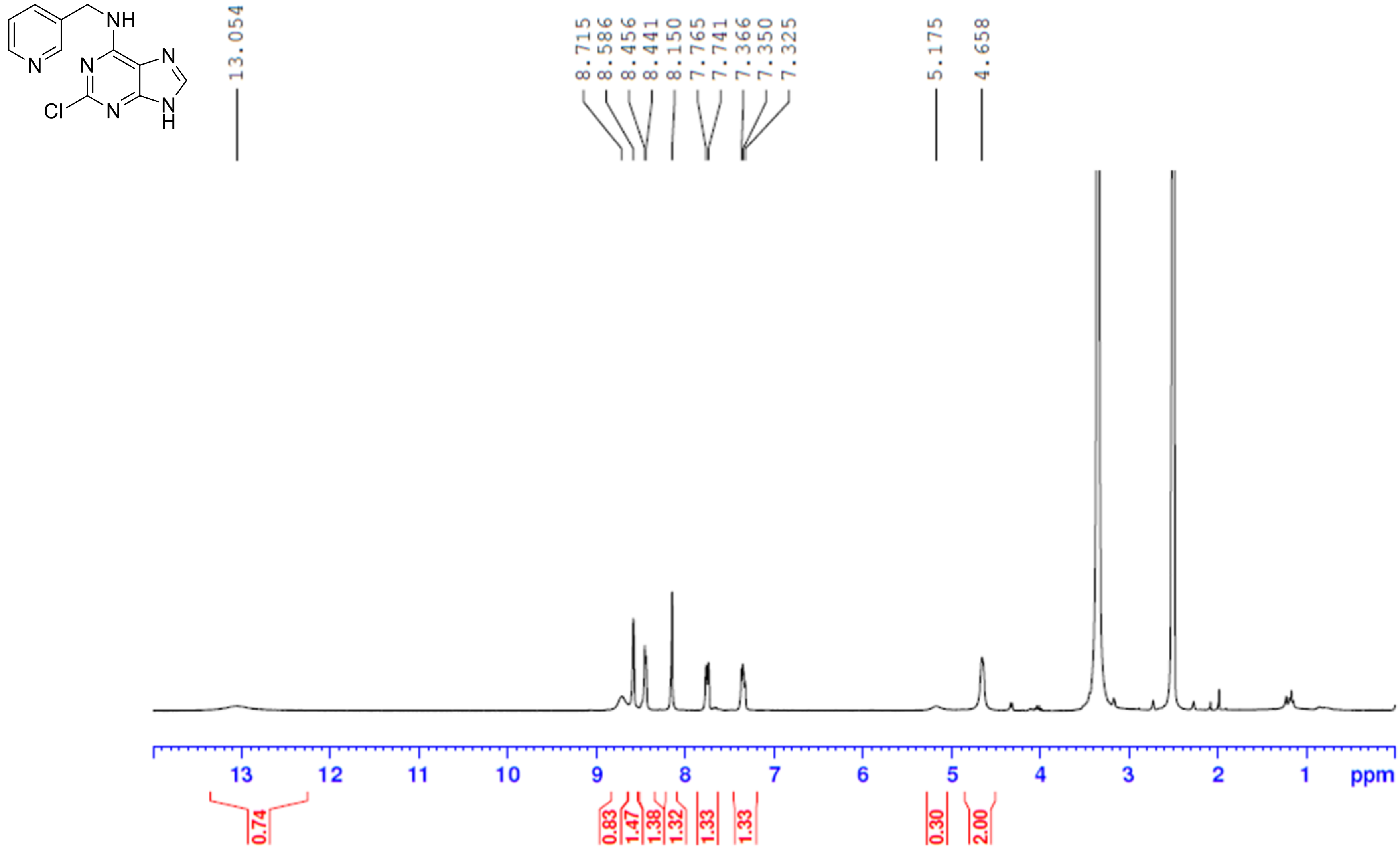

HRMS spectra of compound **3**

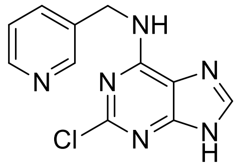

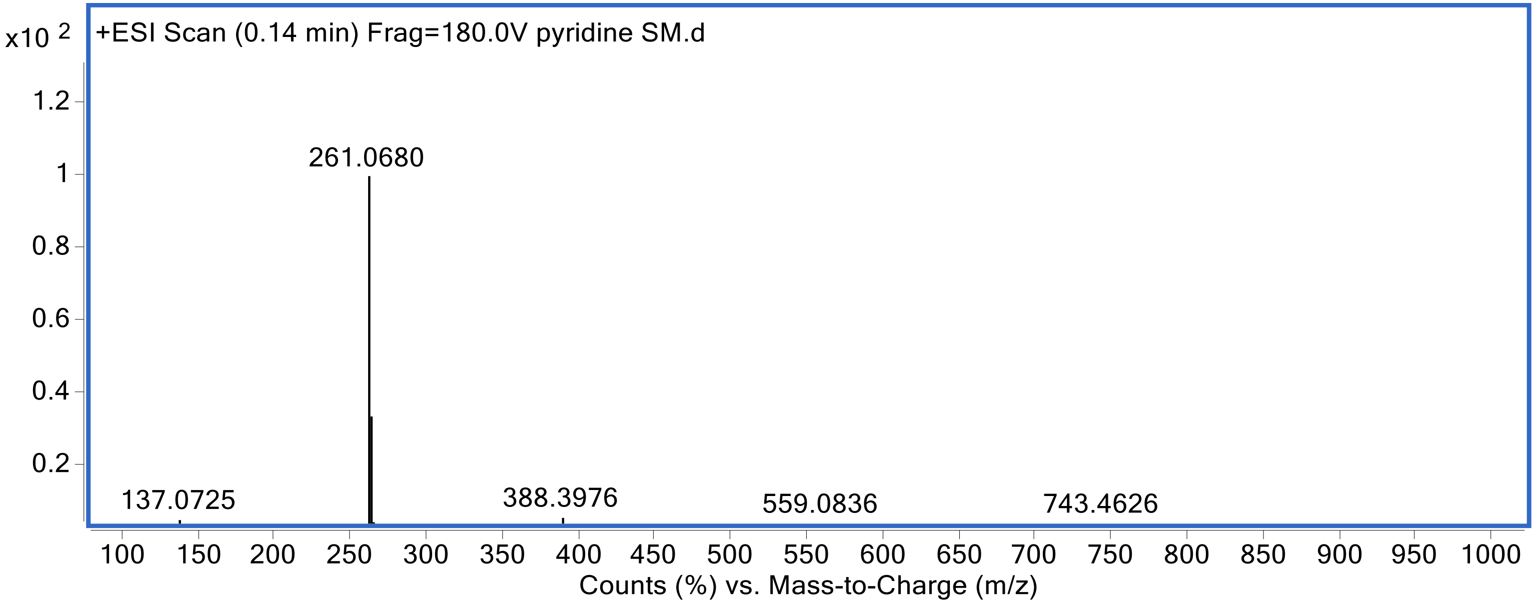

^{1}H NMR spectra of compound **4** measured in MeOH-d_4 at 300 MHz

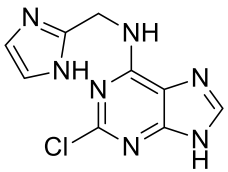

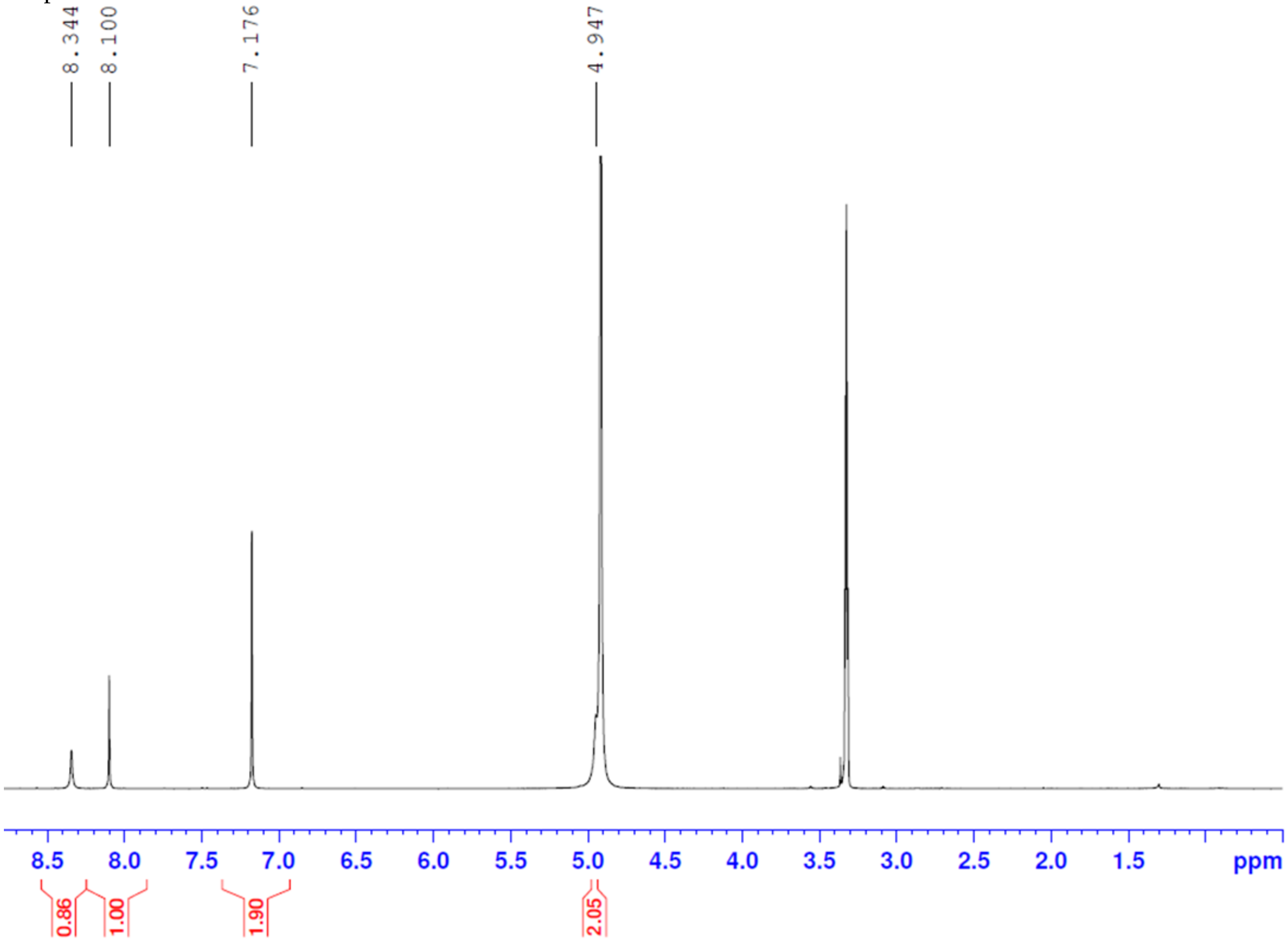

HRMS spectra of compound **4**

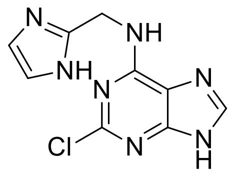

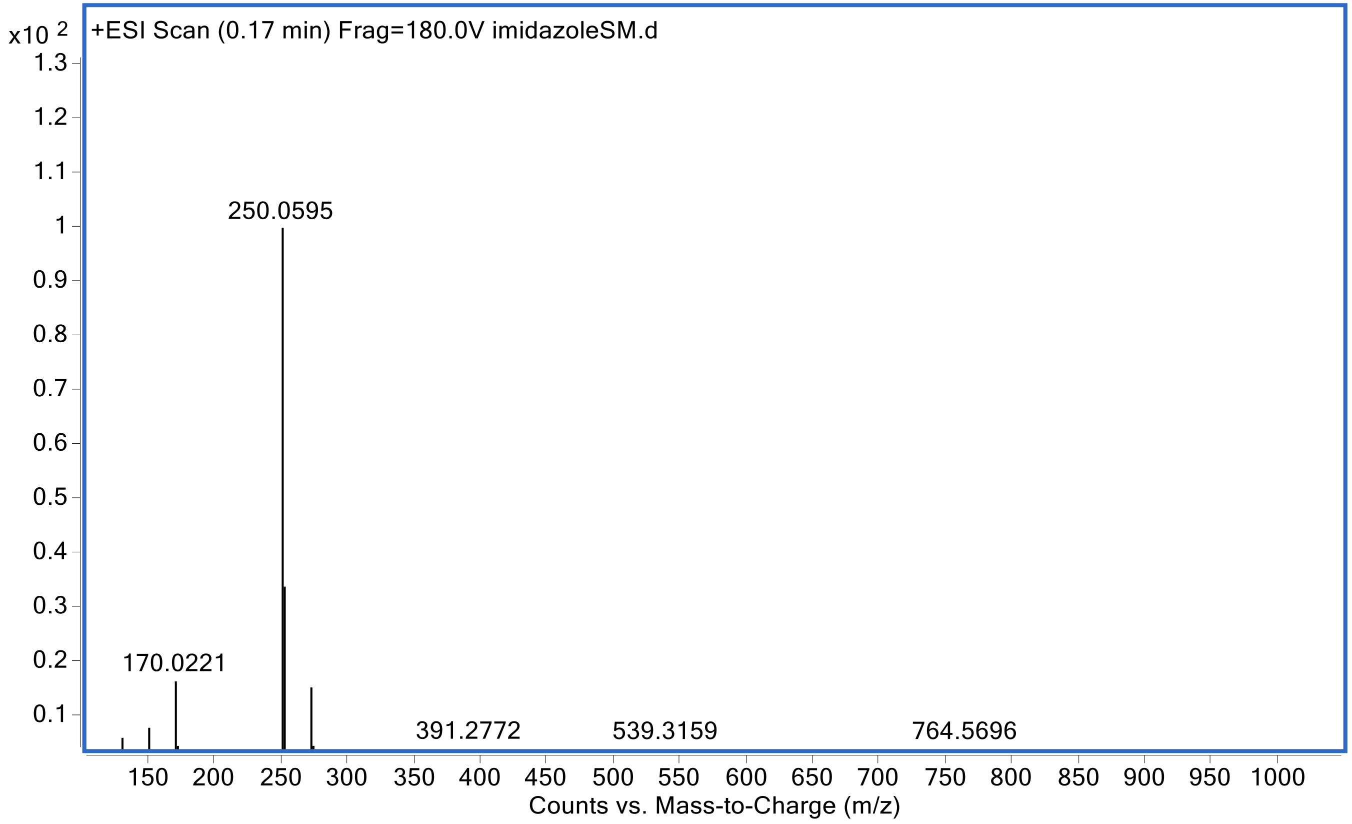

^1H NMR spectra of compound **5** measured in MeOH-d_4 at 300 MHz

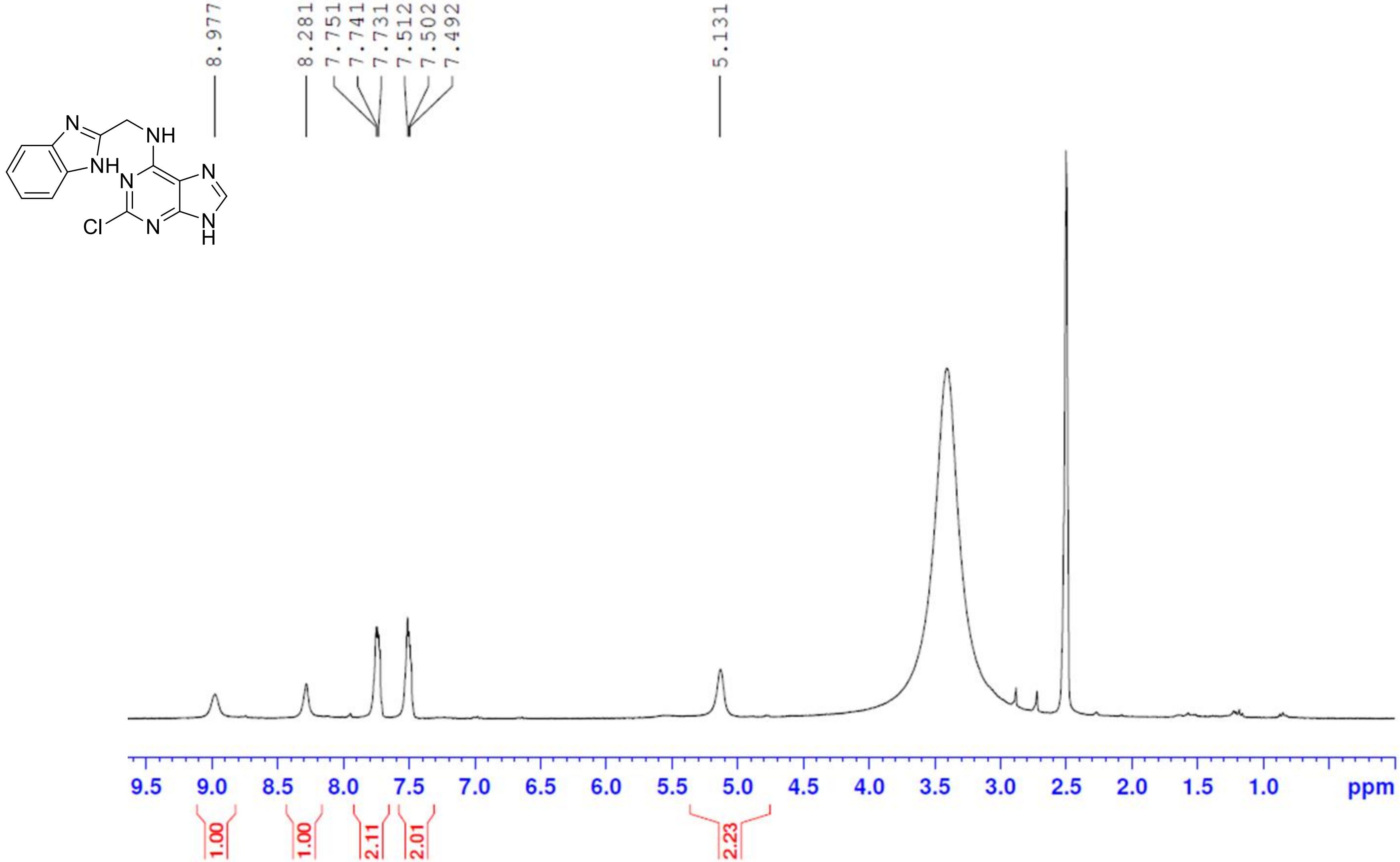

HRMS spectra of compound **5**

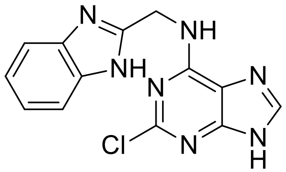

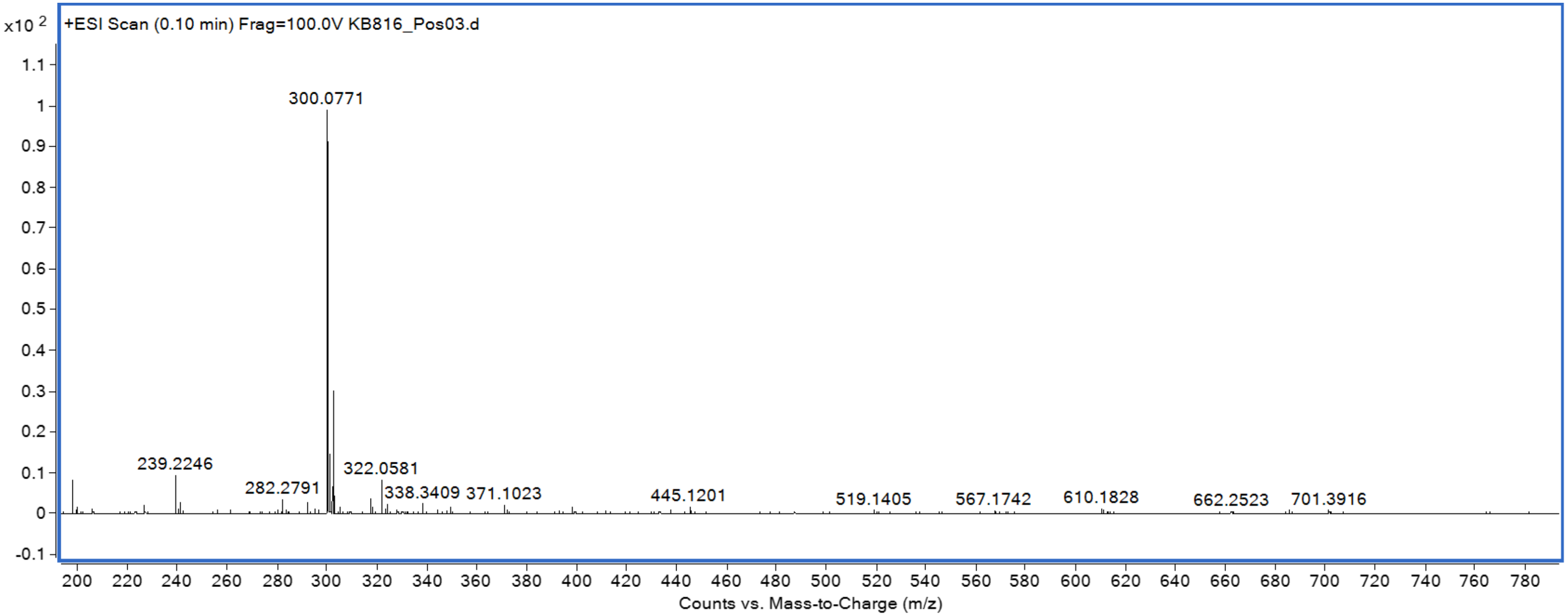

^{1}H NMR spectra of compound **6** measured in MeOH-d_4 at 300 MHz

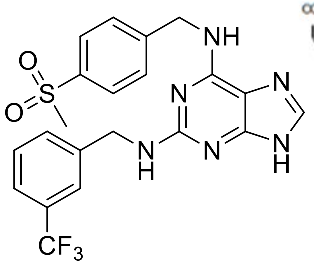

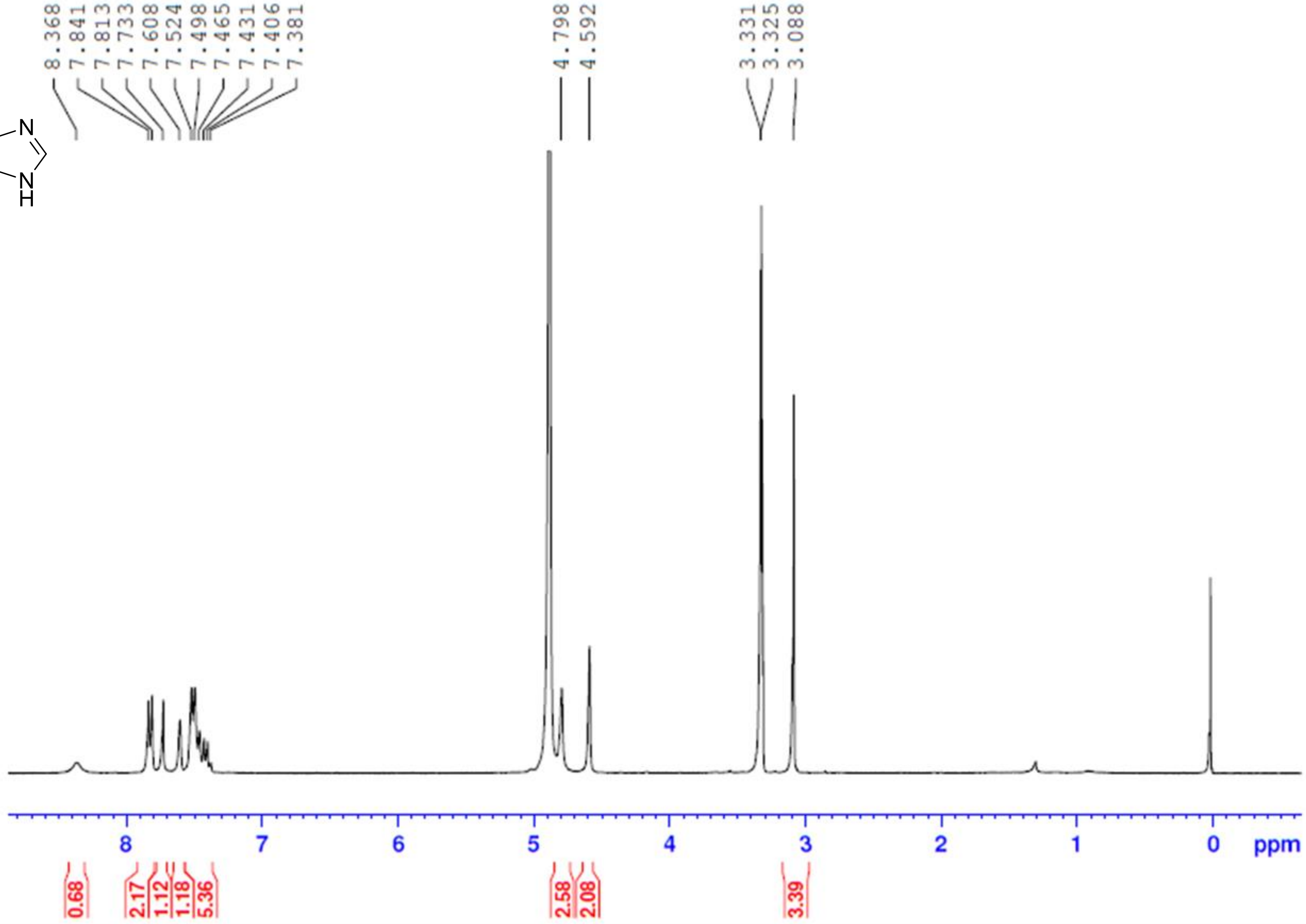

HRMS spectra of compound **6**

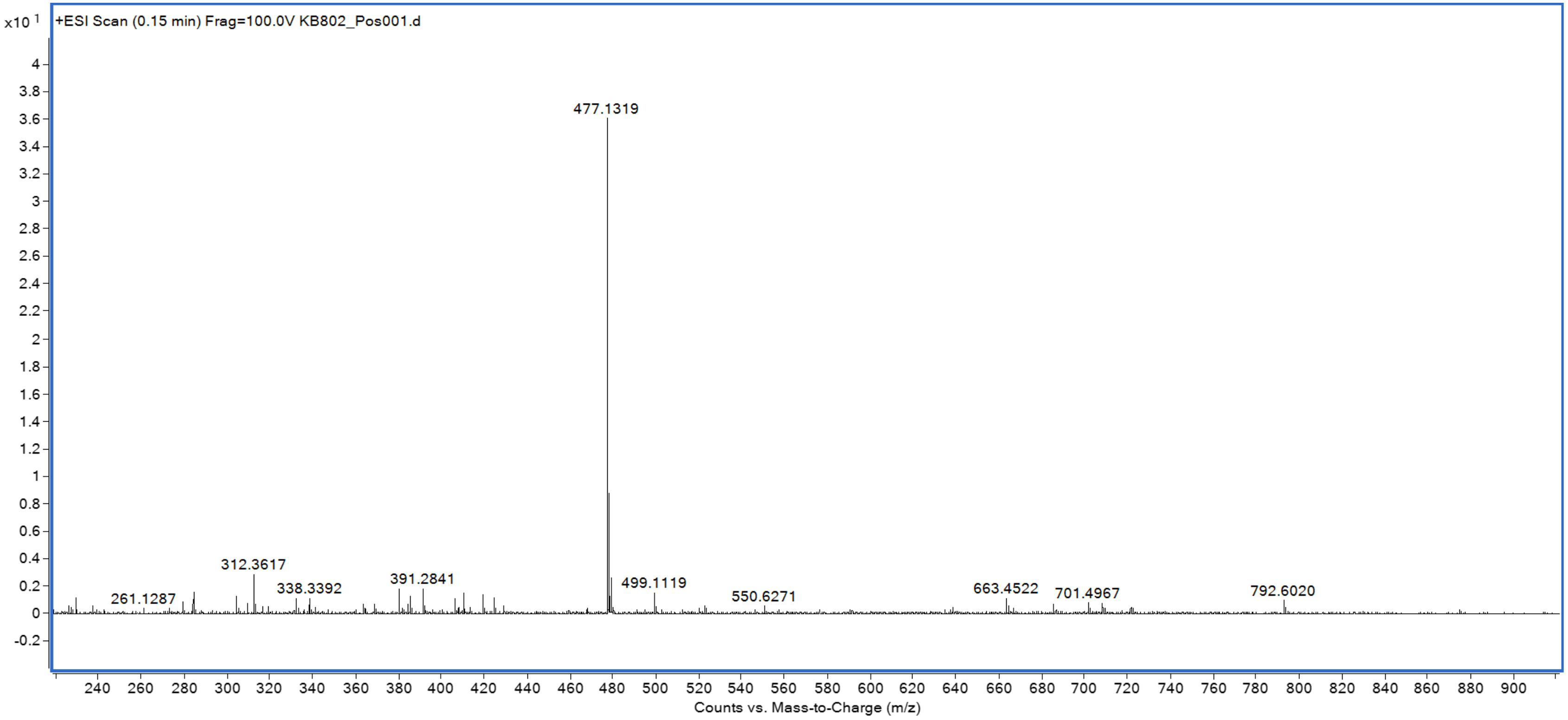

Binding spectra of compound **6** in CYP3A4

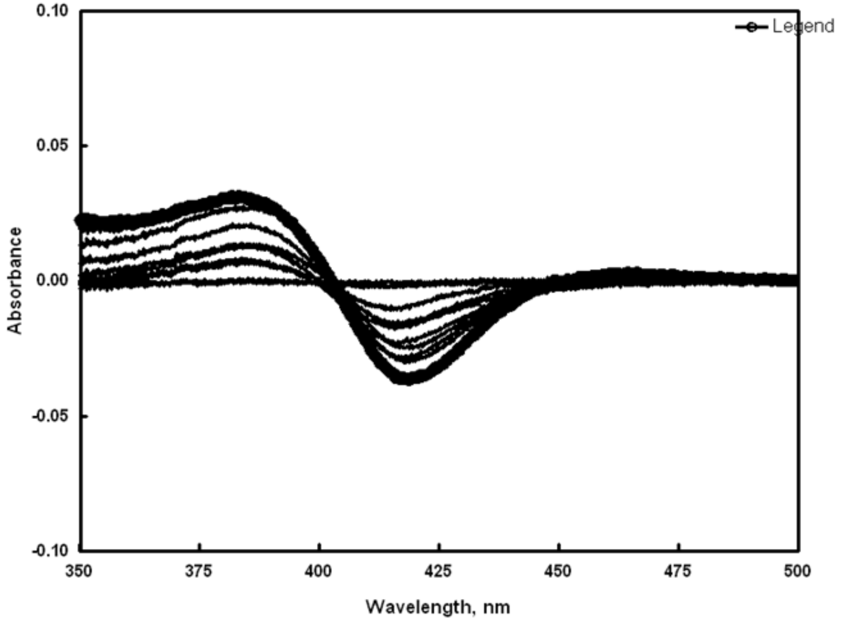

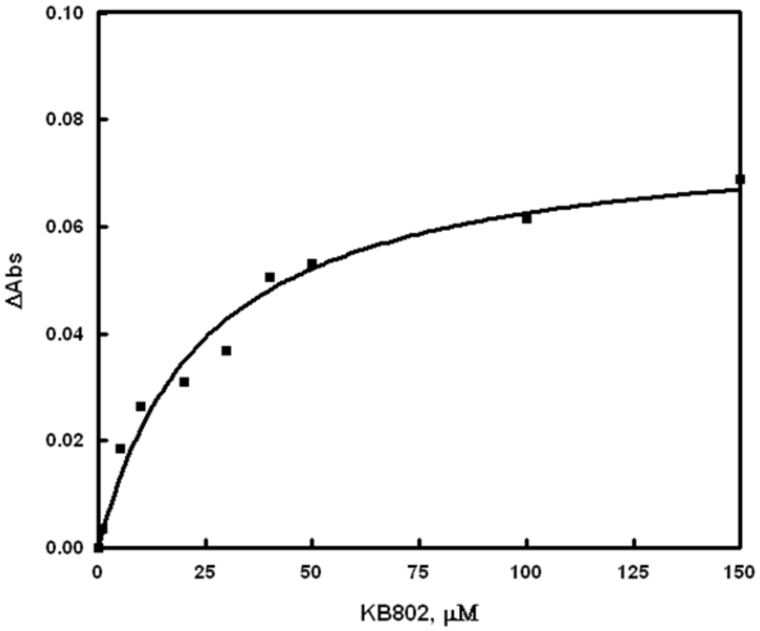

^{1}H NMR spectra of compound **7** measured in MeOH-d_4 at 300 MHz

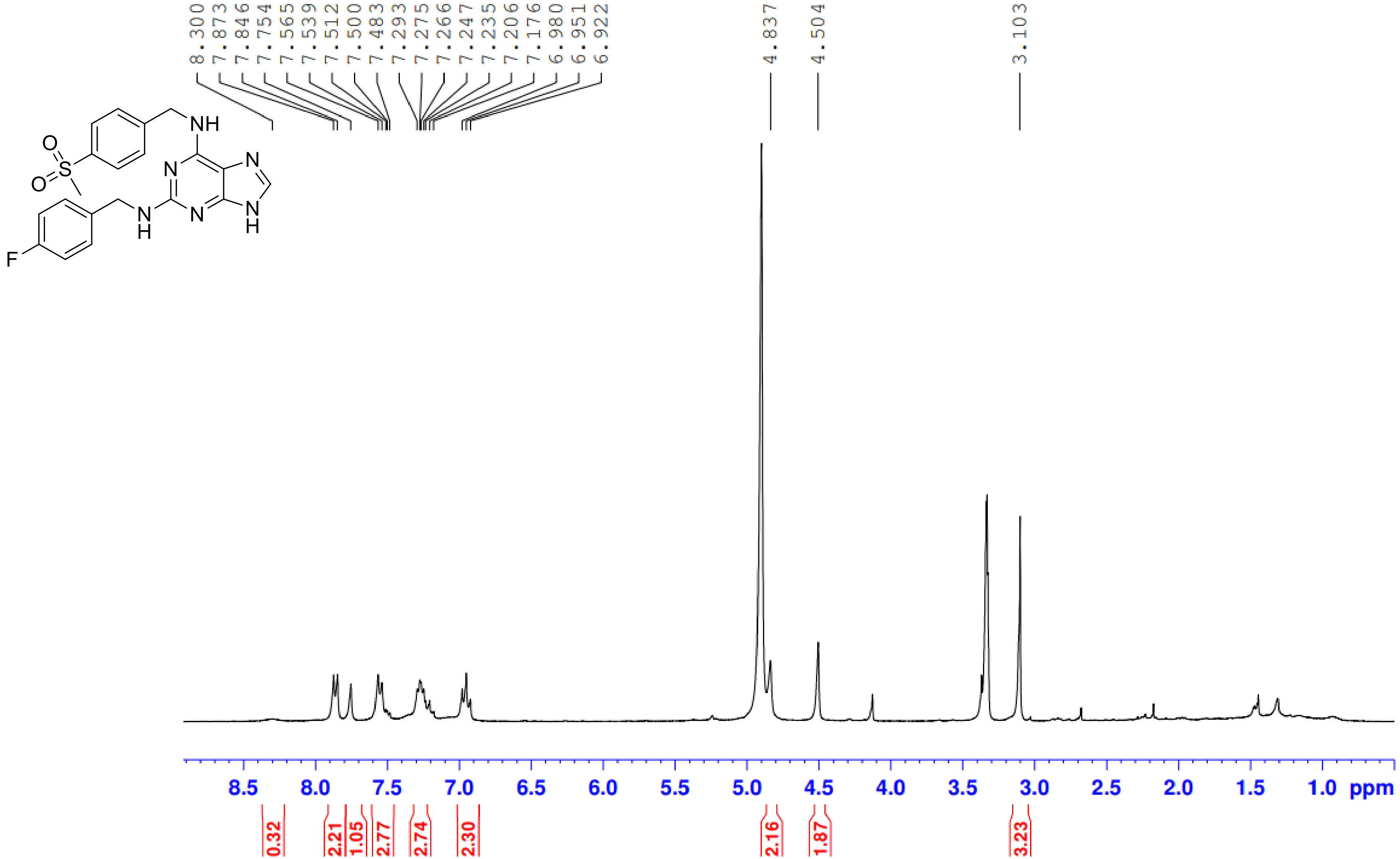

HRMS spectra of compound **7**

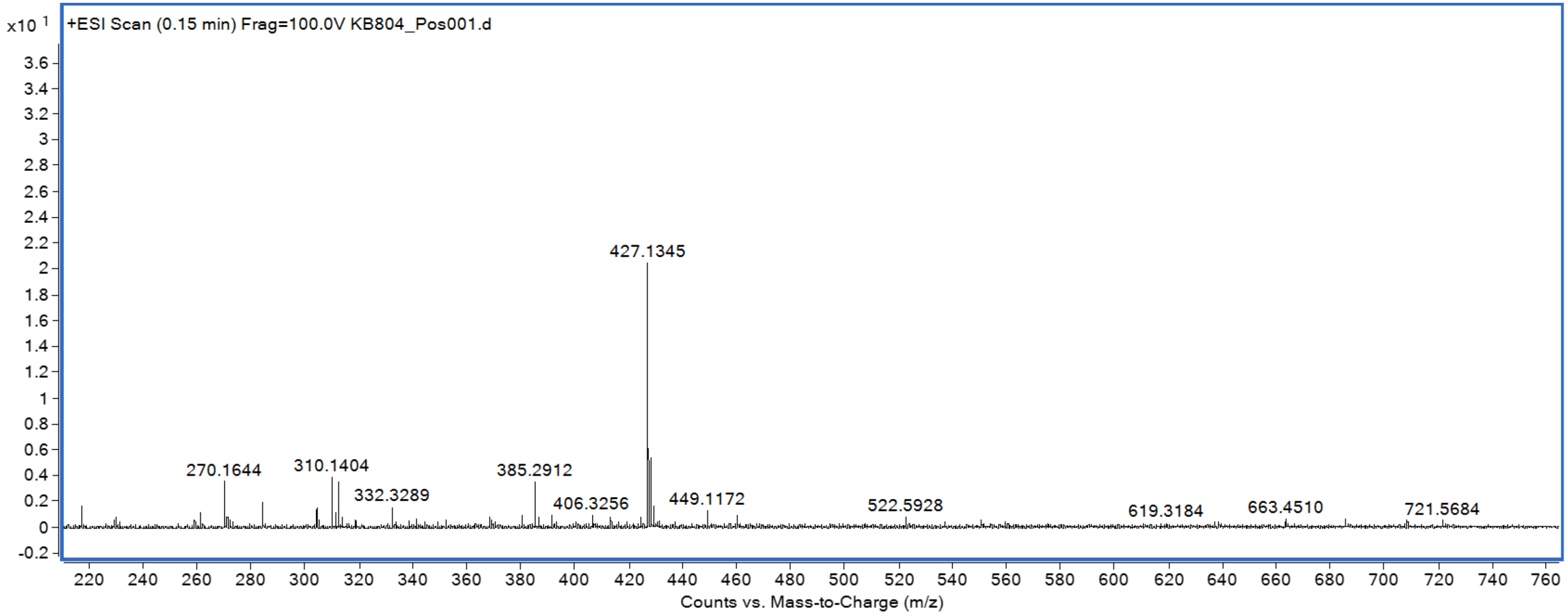

Binding spectra of compound **7** in CYP3A4

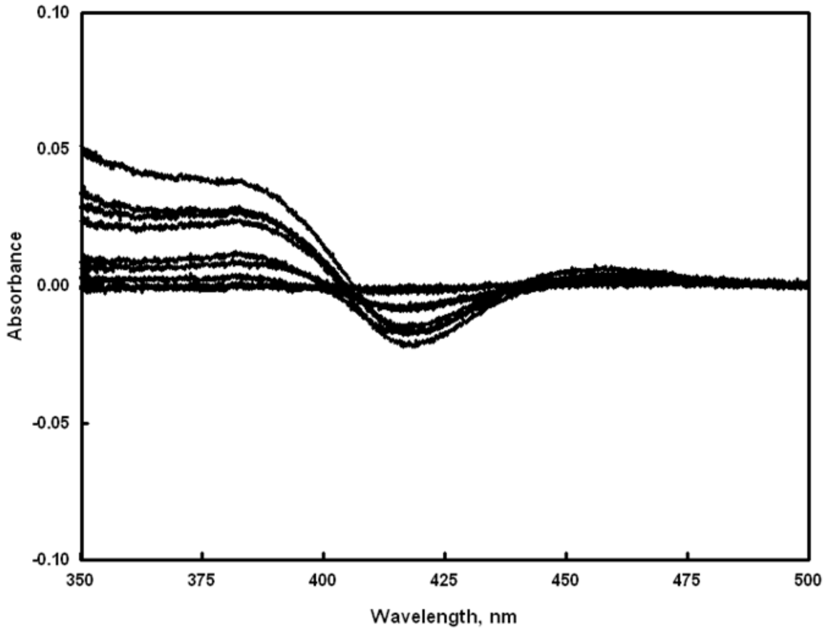

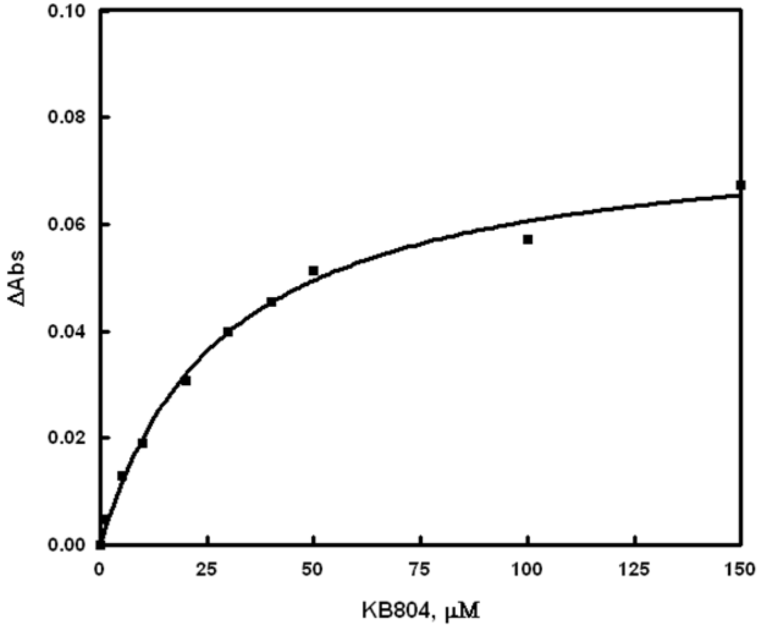

^{1}H NMR spectra of compound **8** measured in DMSO-d_6 at 300 MHz

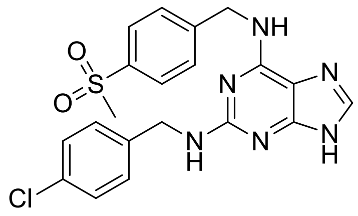

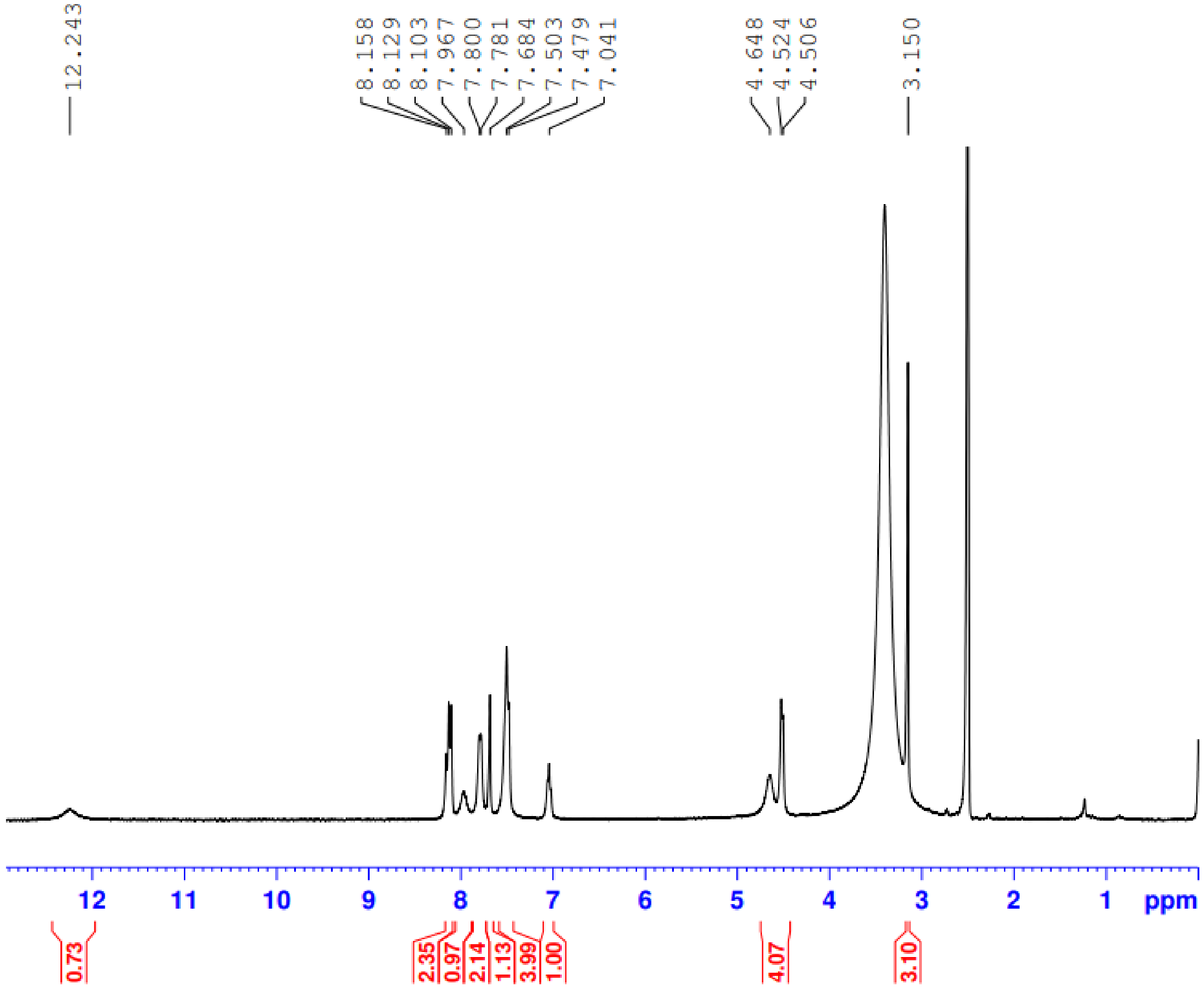

HRMS spectra of compound **8**

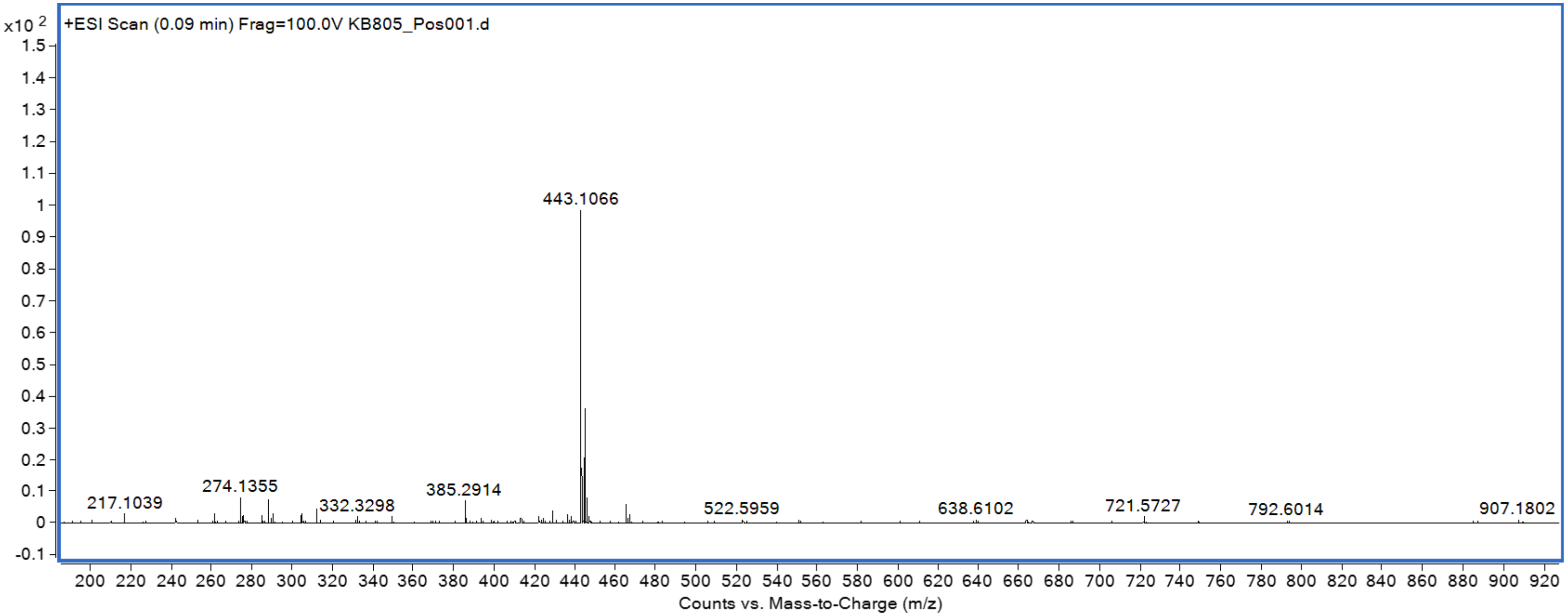

Binding spectra of compound **8** in CYP3A4

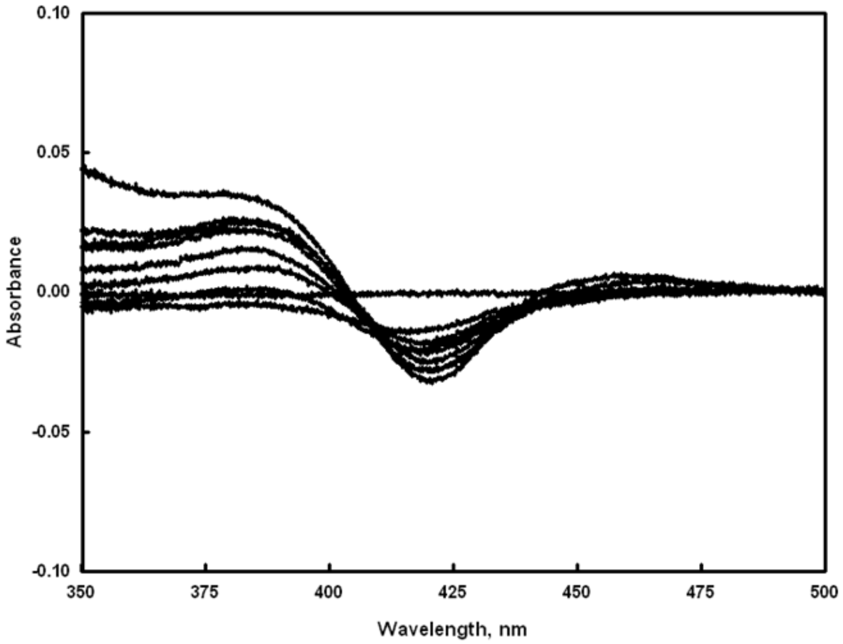

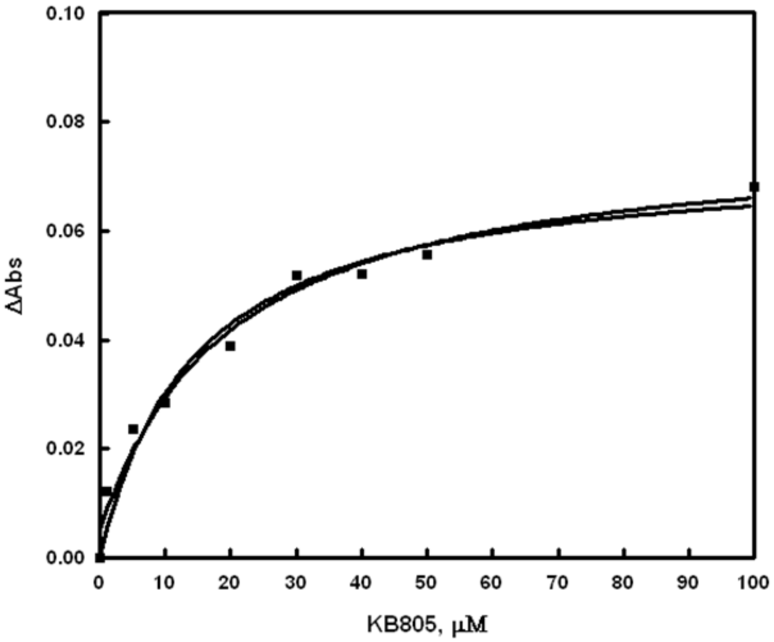

^{1}H NMR spectra of compound **9** measured in DMSO-d_6 at 300 MHz

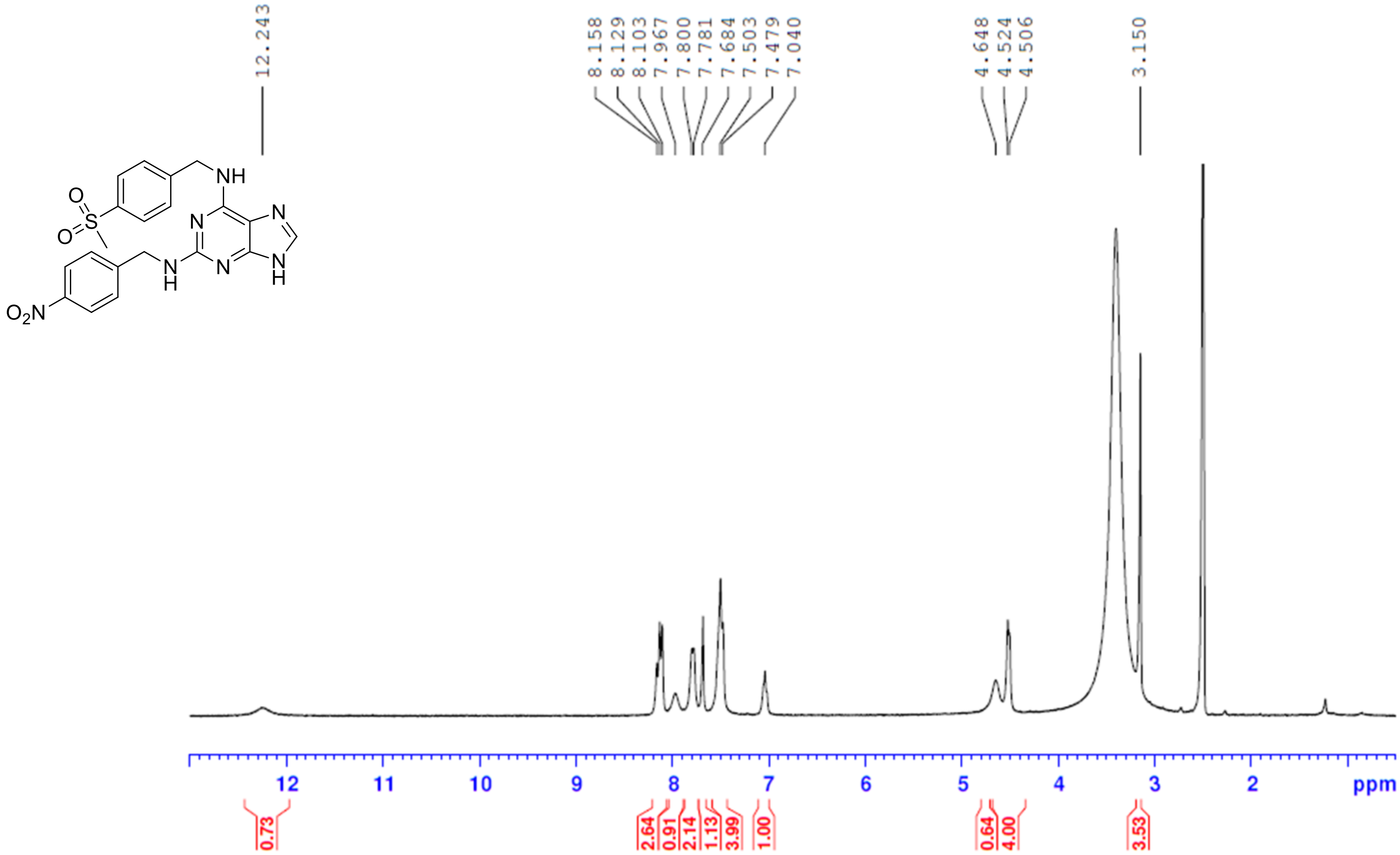

HRMS spectra of compound **9**

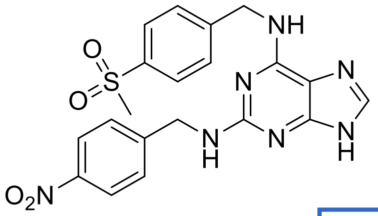

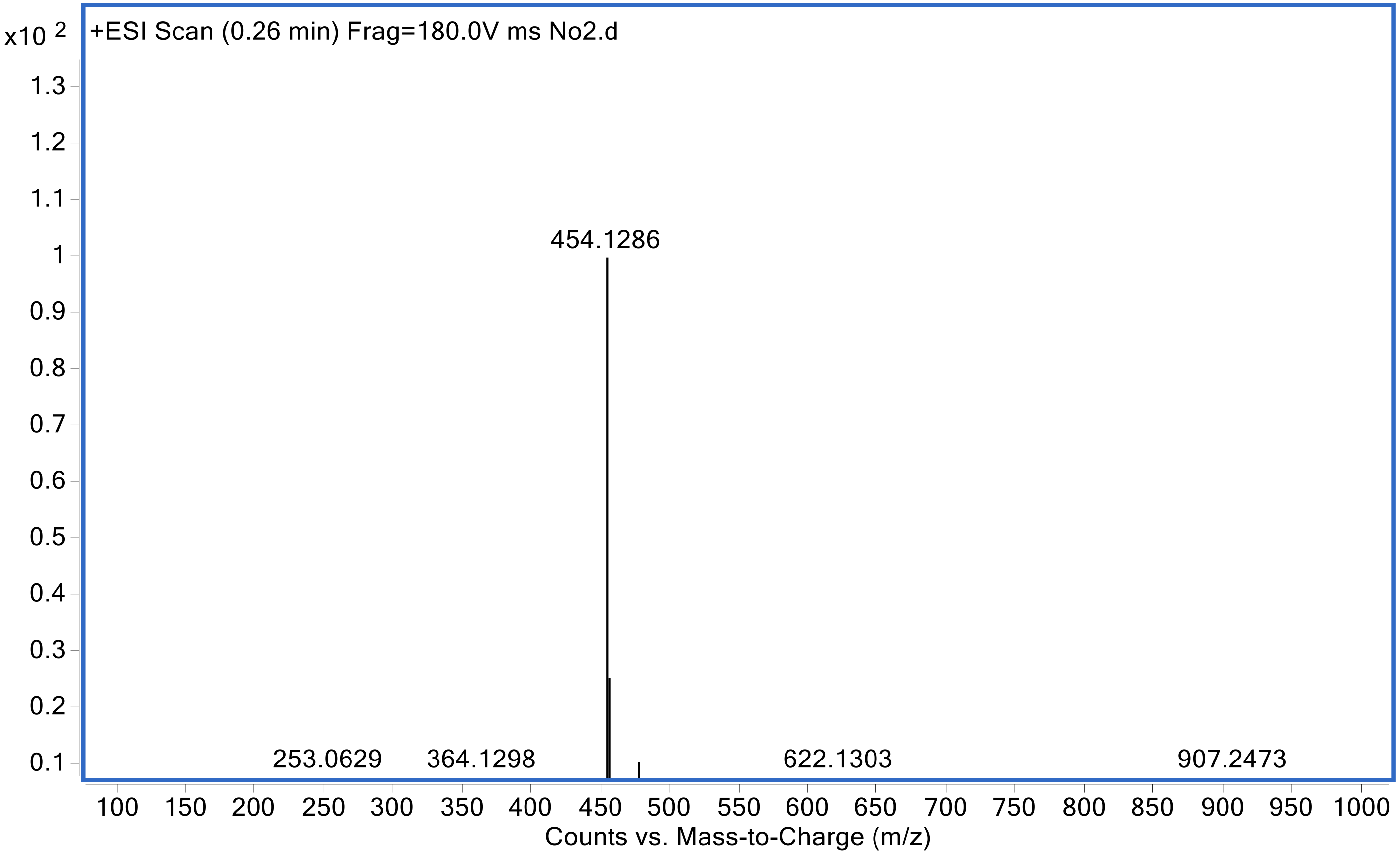

^{1}H NMR spectra of compound **10** measured in DMSO-d_6 at 300 MHz

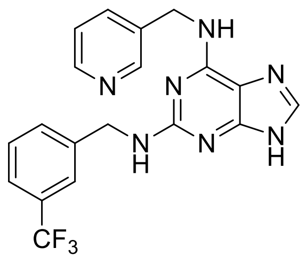

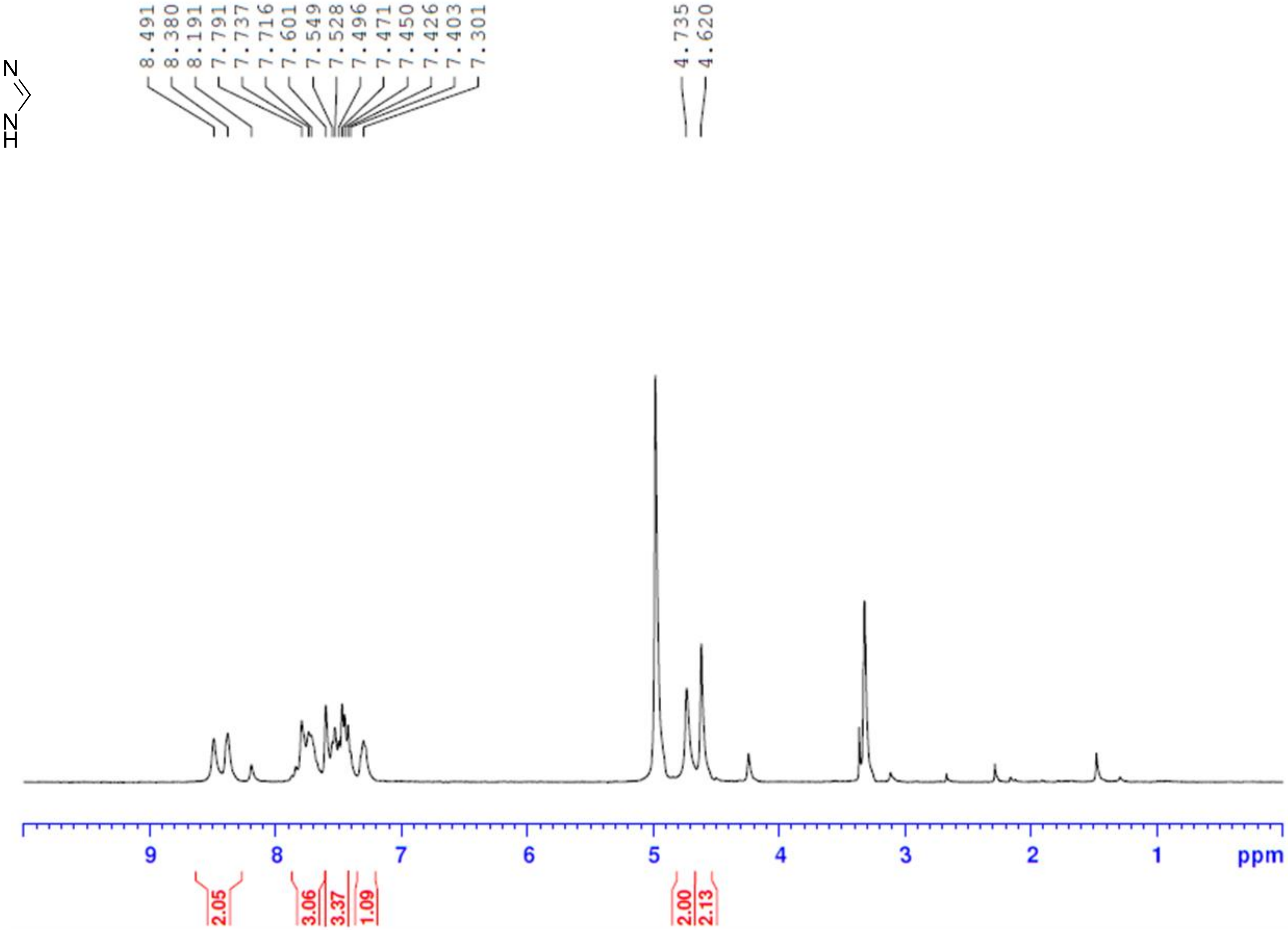

HRMS spectra of compound **10**

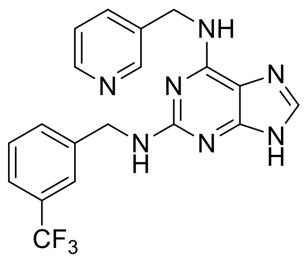

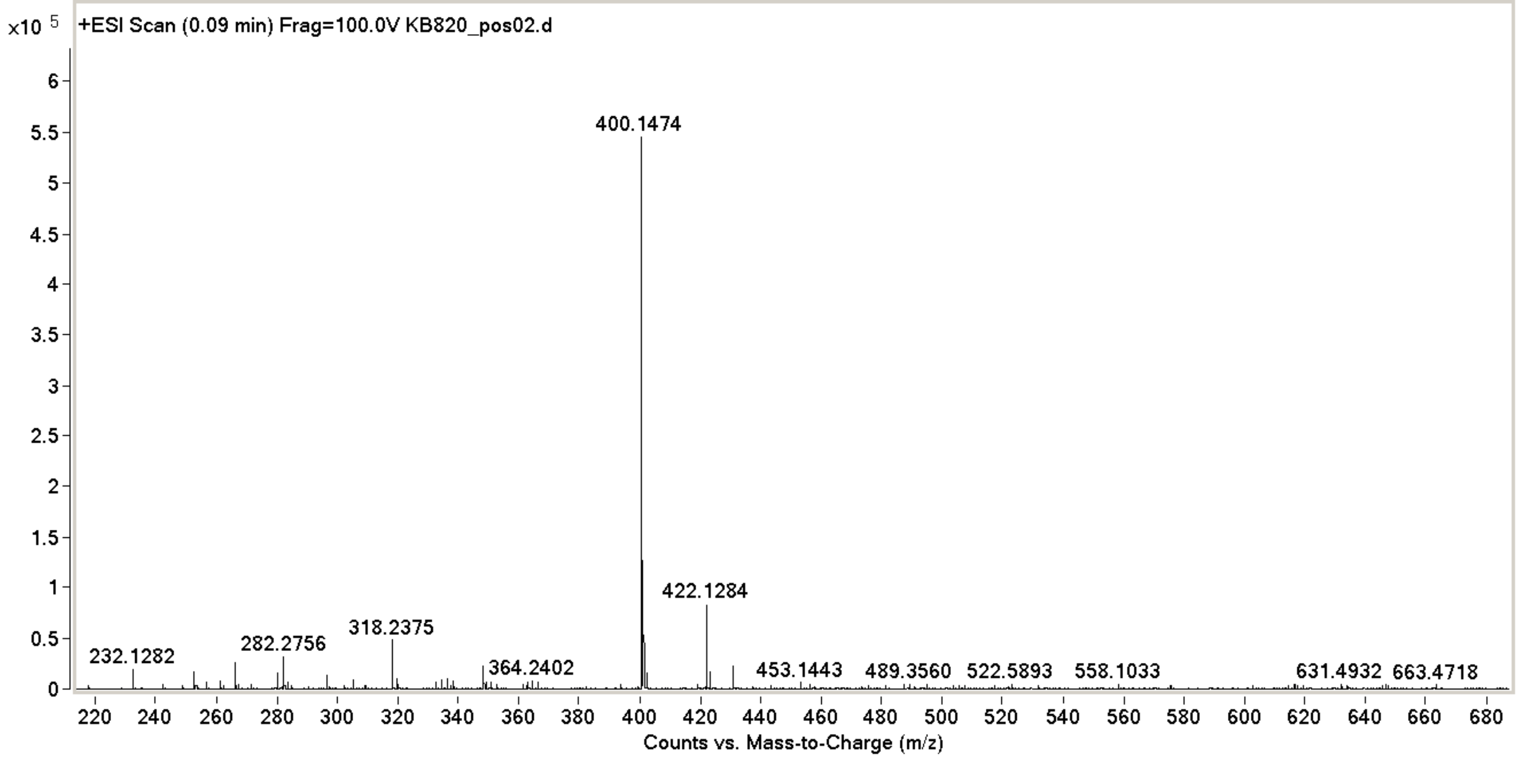

Binding spectra of compound **10** in CYP3A4

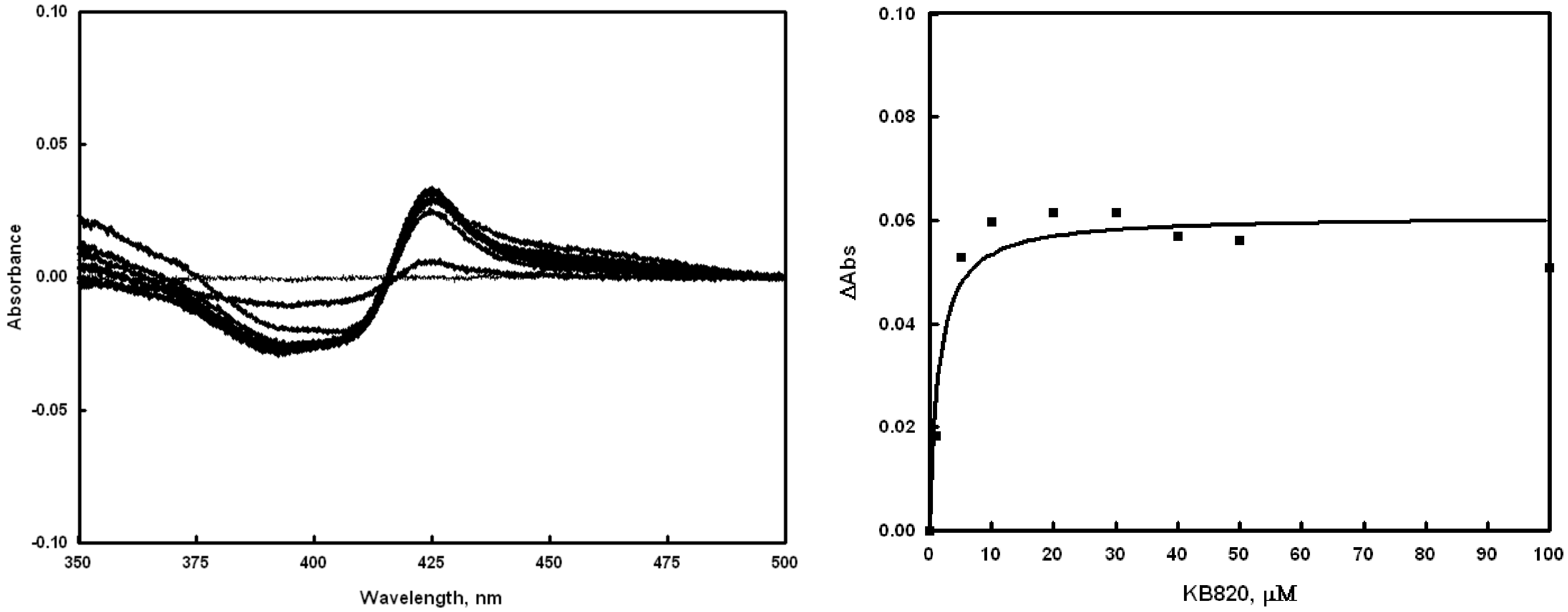

^{1}H NMR spectra of compound **11** measured in MeOH-d_4 at 300 MHz

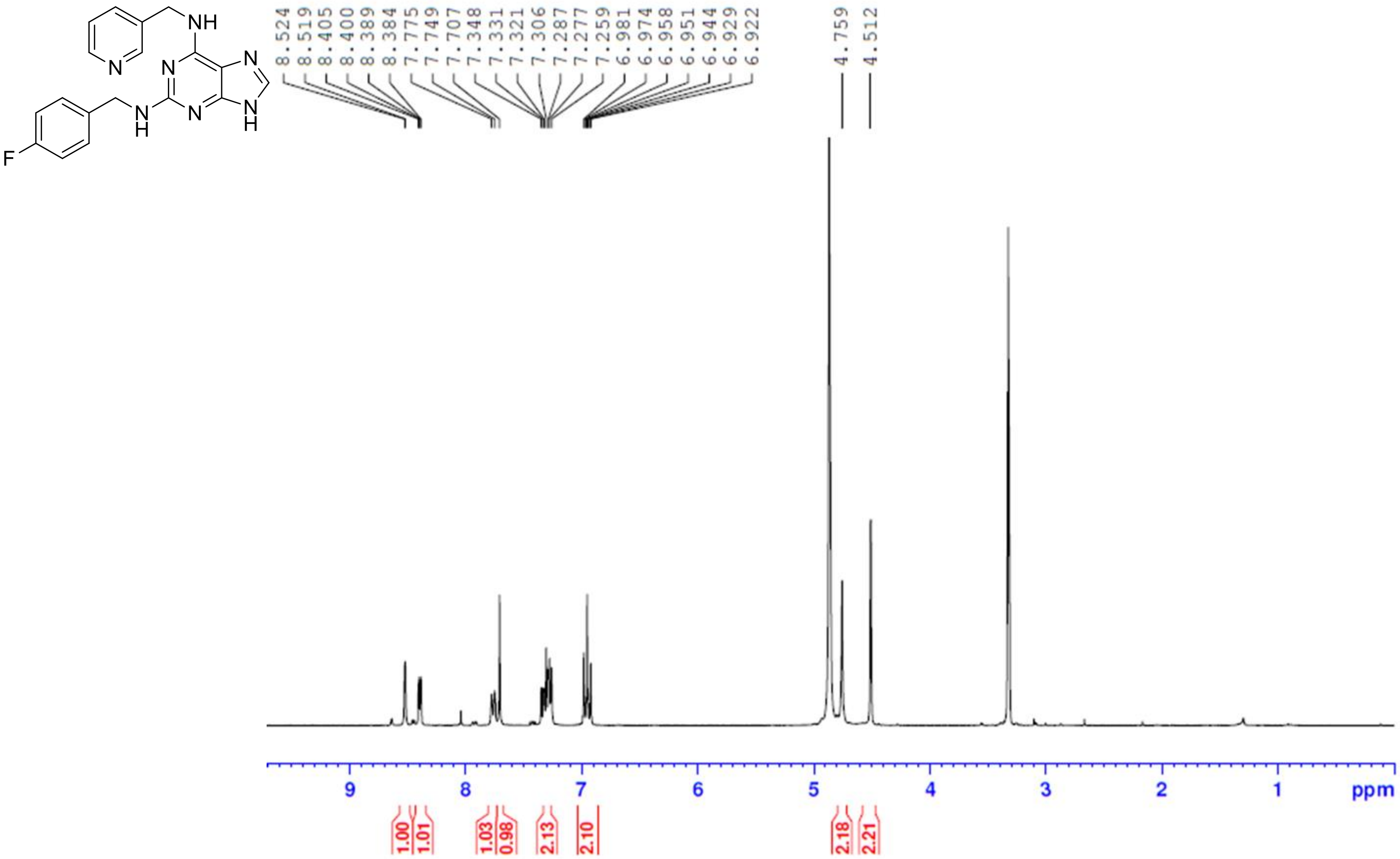

HRMS spectra of compound **11**

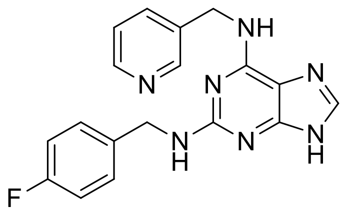

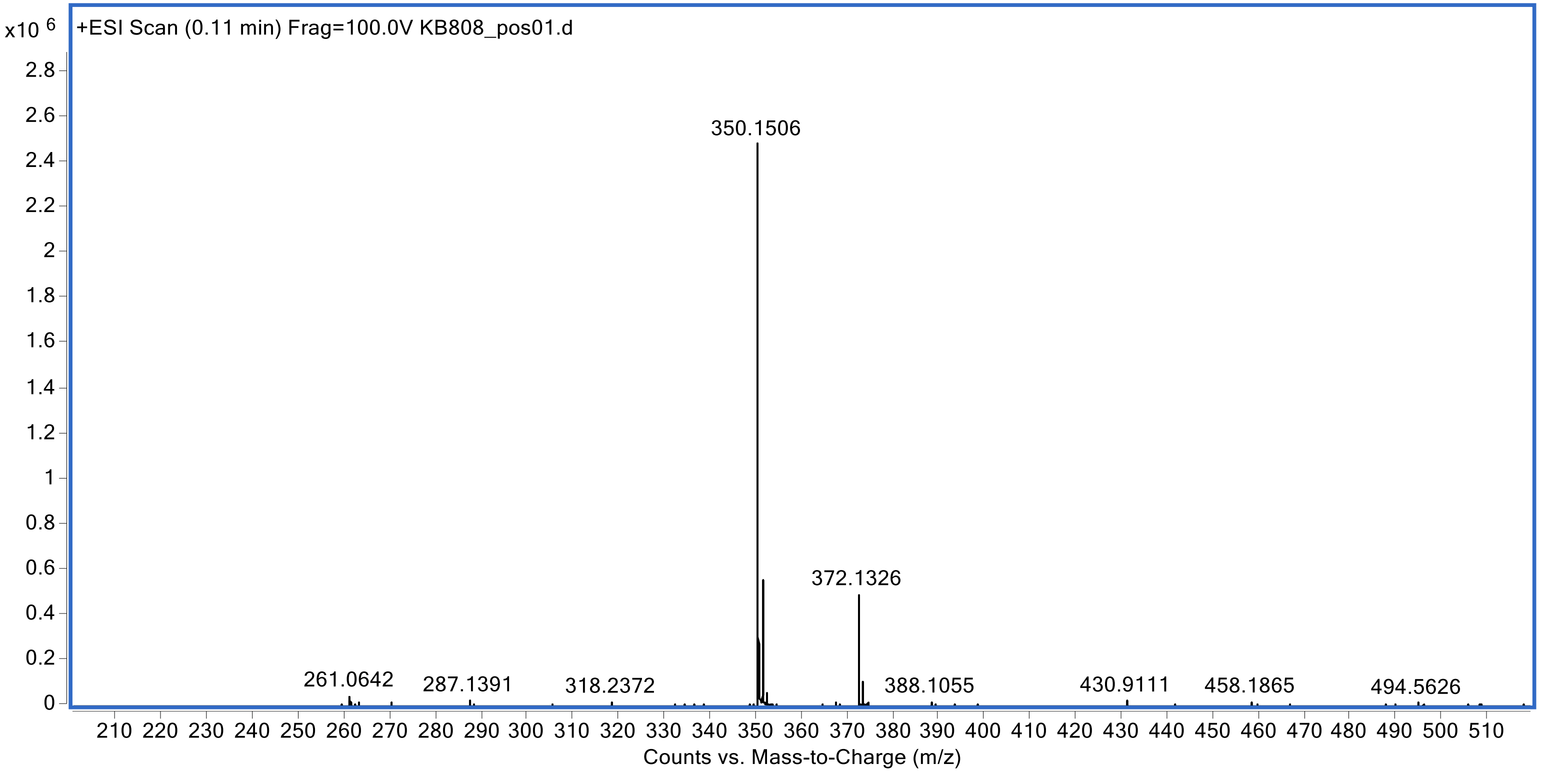

Binding spectra of compound **11** in CYP3A4

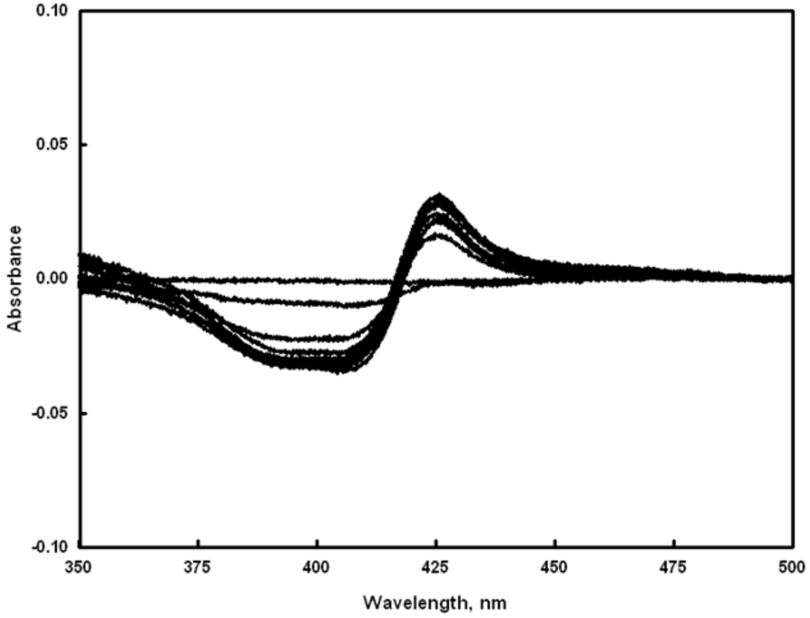

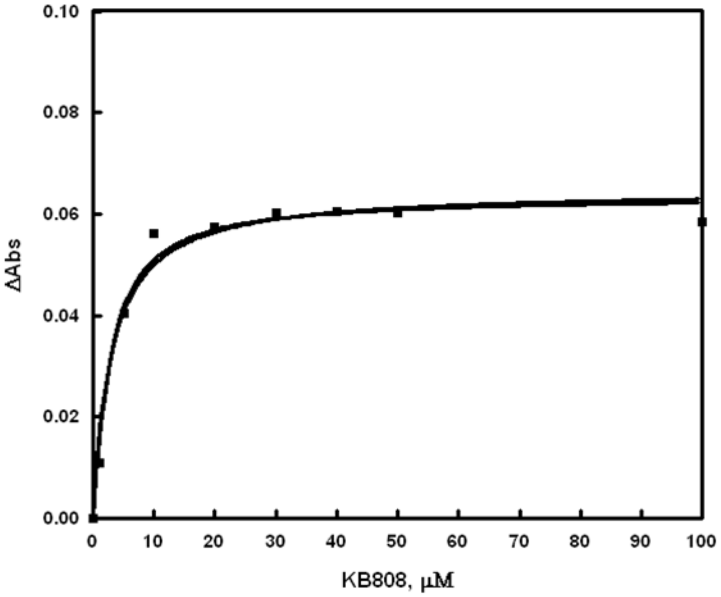

^{1}H NMR spectra of compound **12** measured in MeOH-d_4 at 300 MHz

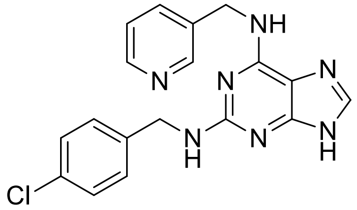

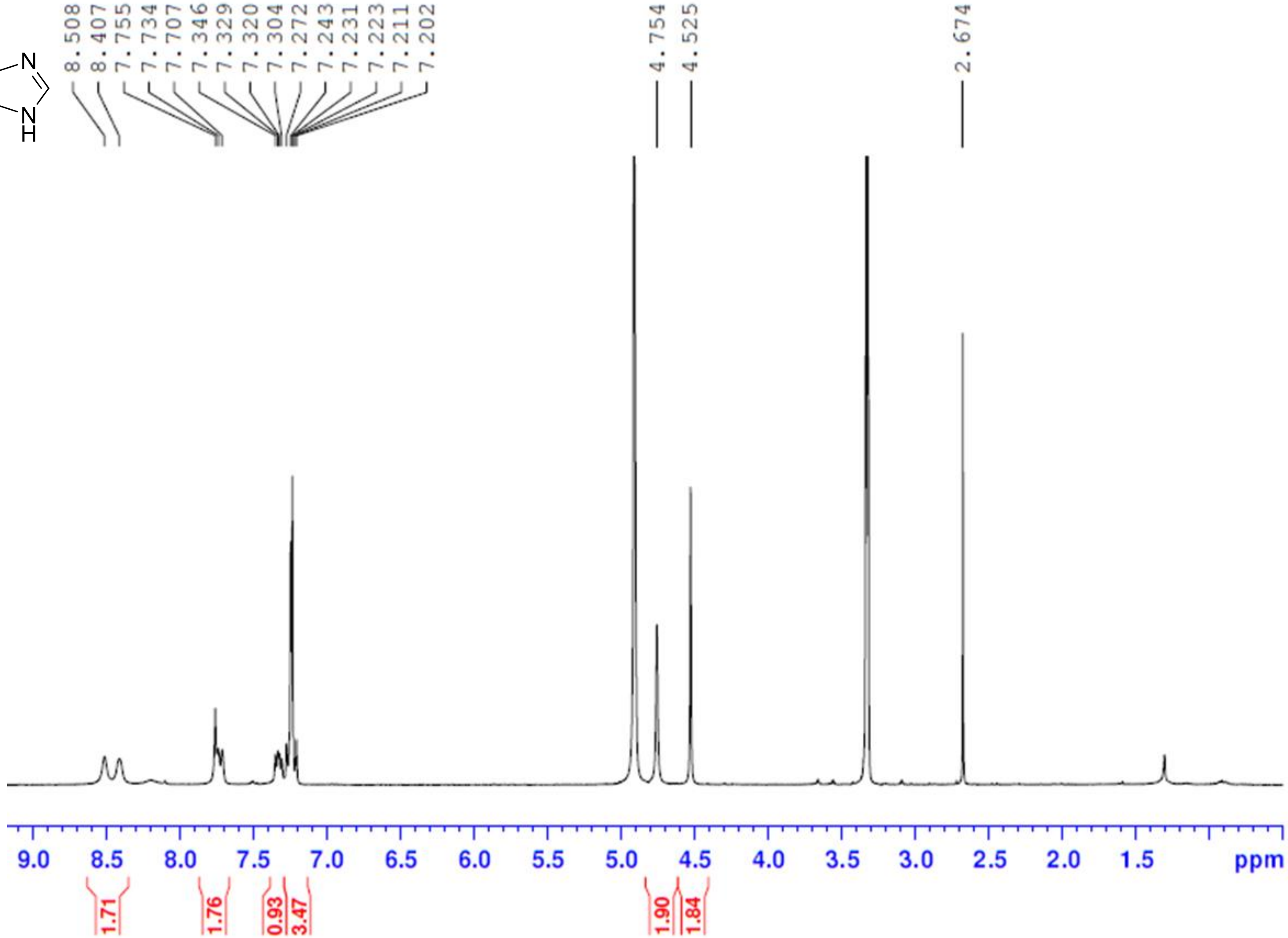

HRMS spectra of compound **12**

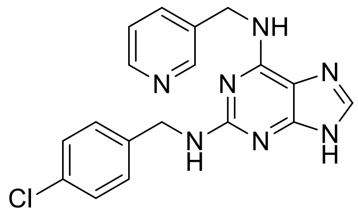

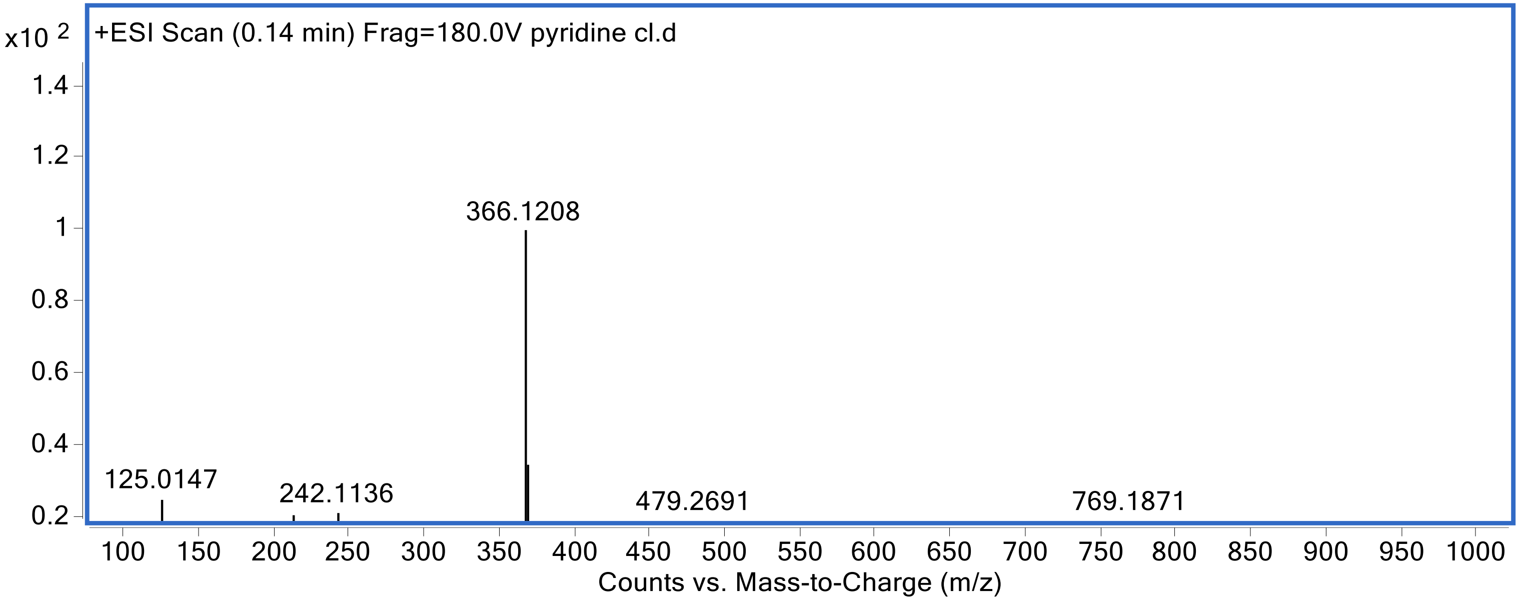

Binding spectra of compound **12** in CYP3A4

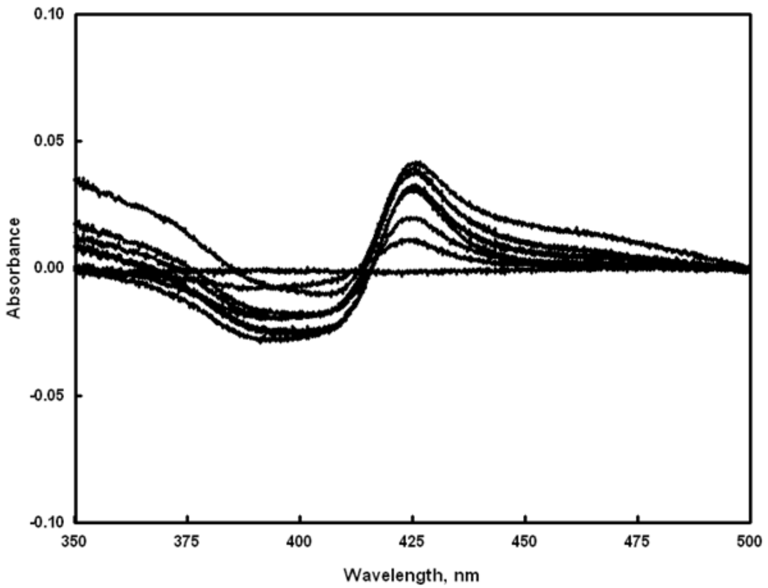

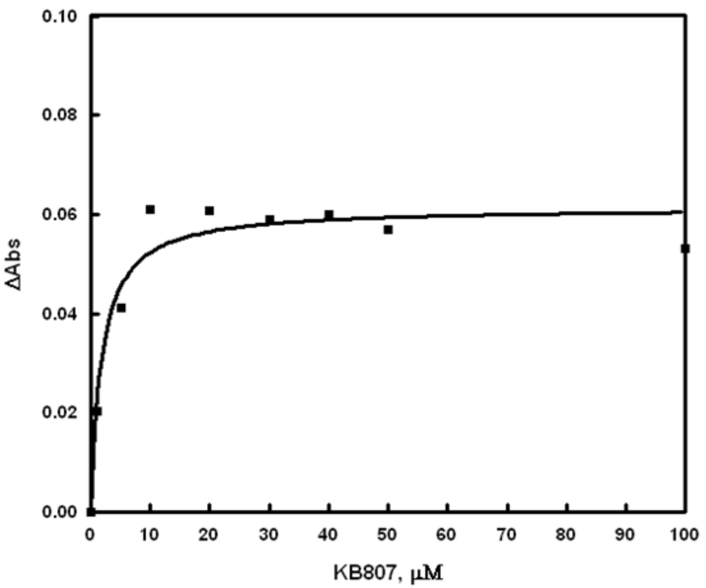

^{1}H NMR spectra of compound **13 (KB 806)** measured in DMSO-d_6 at 300 MHz

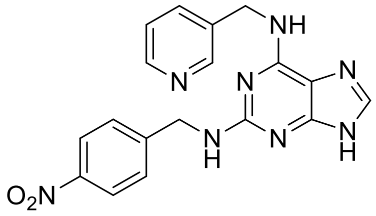

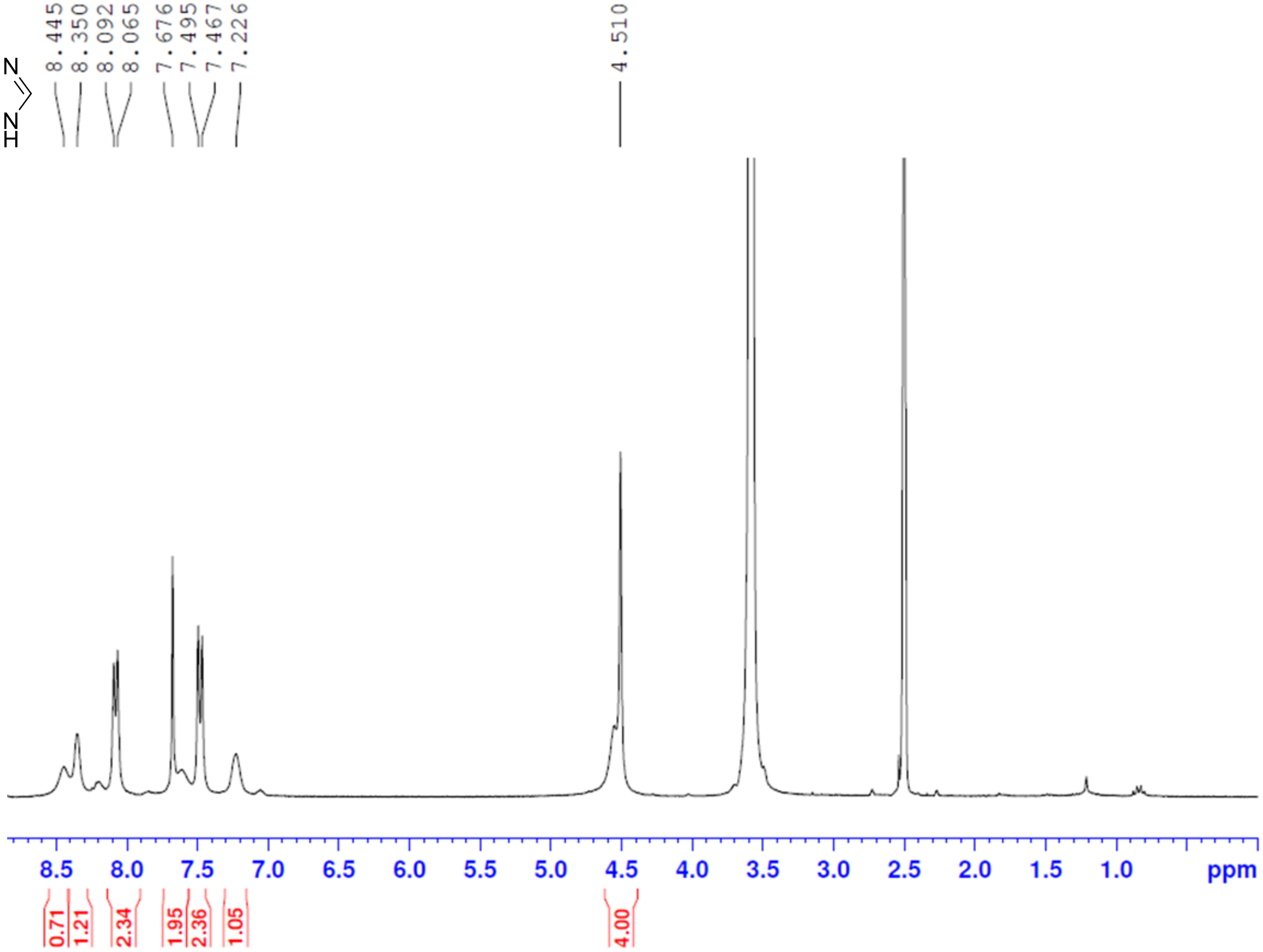

HRMS spectra of compound **13(KB 806)**

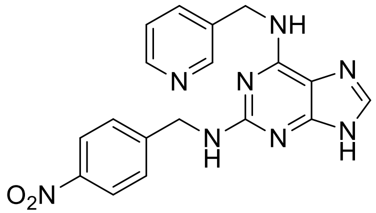

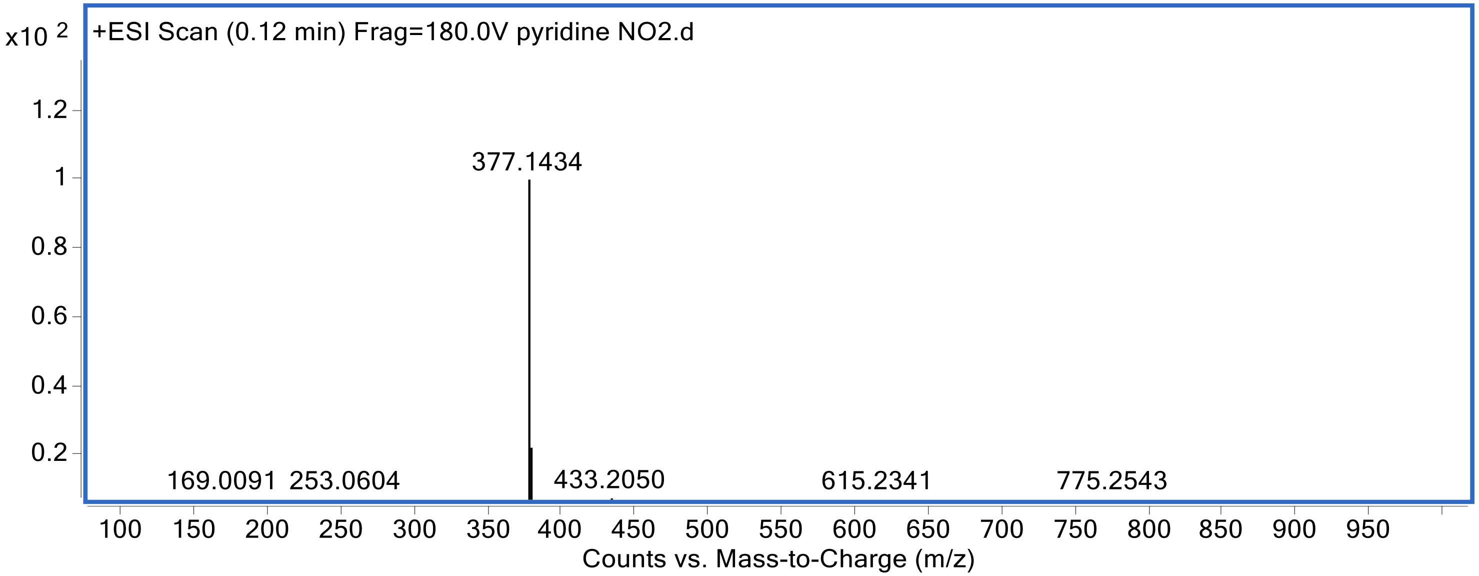

Binding spectra of compound **13** in CYP3A4

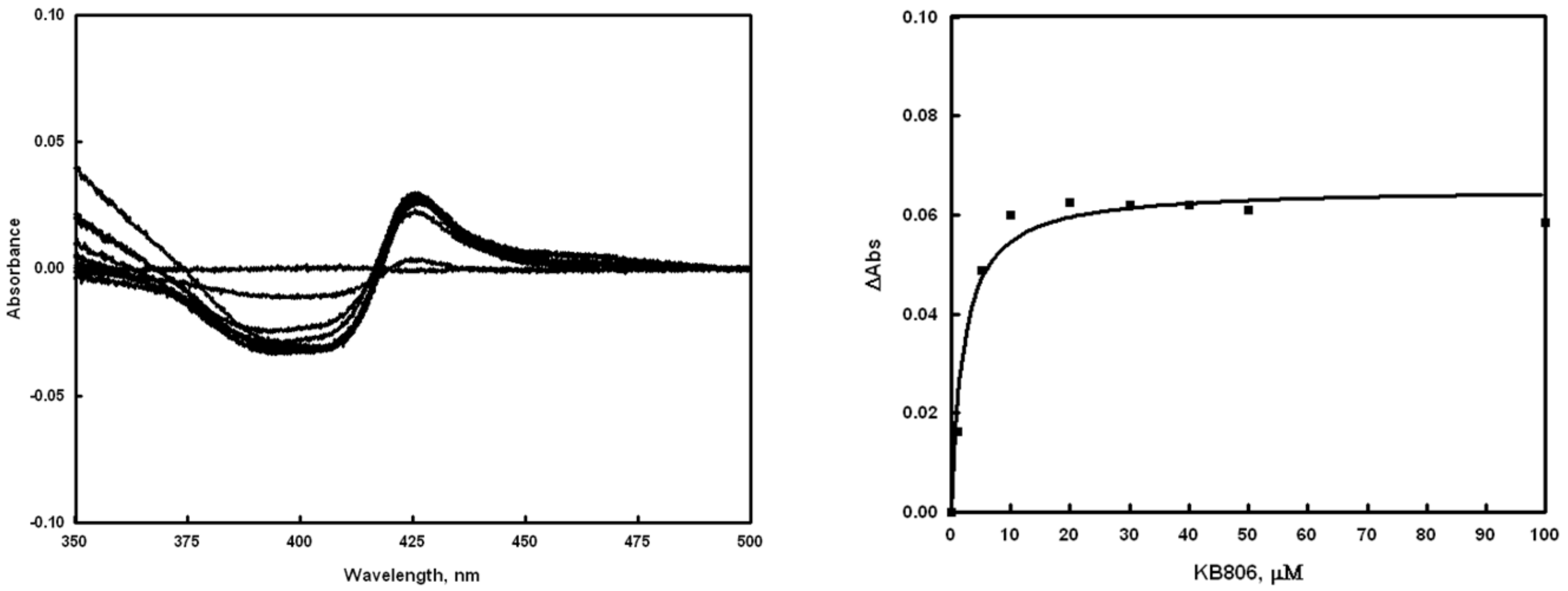

^{1}H NMR spectra of compound **14** measured in MeOH-d_4 at 300 MHz

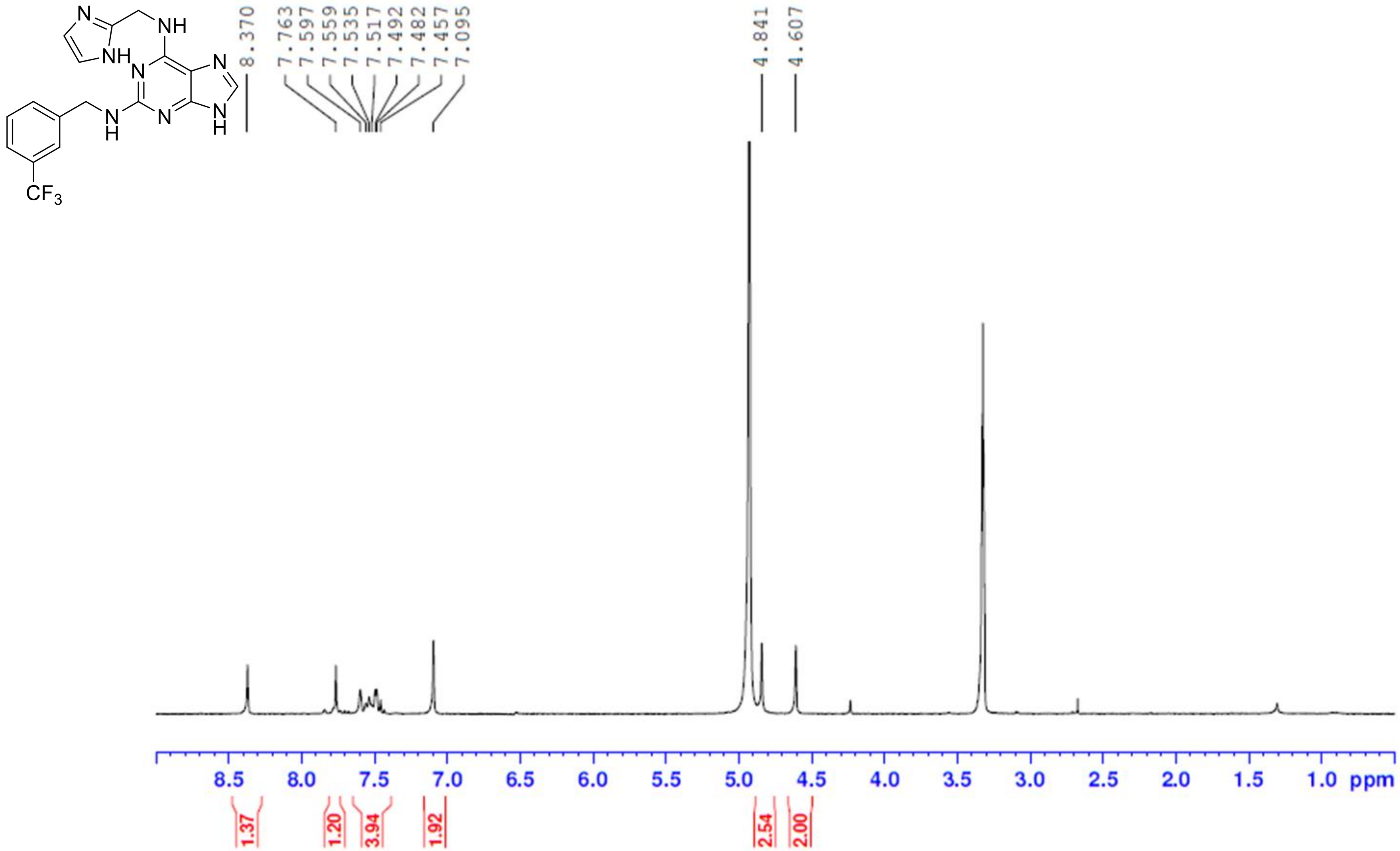

HRMS spectra of compound **14**

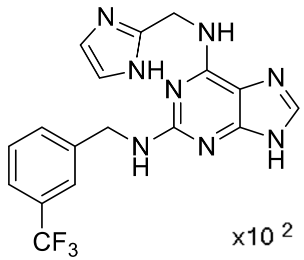

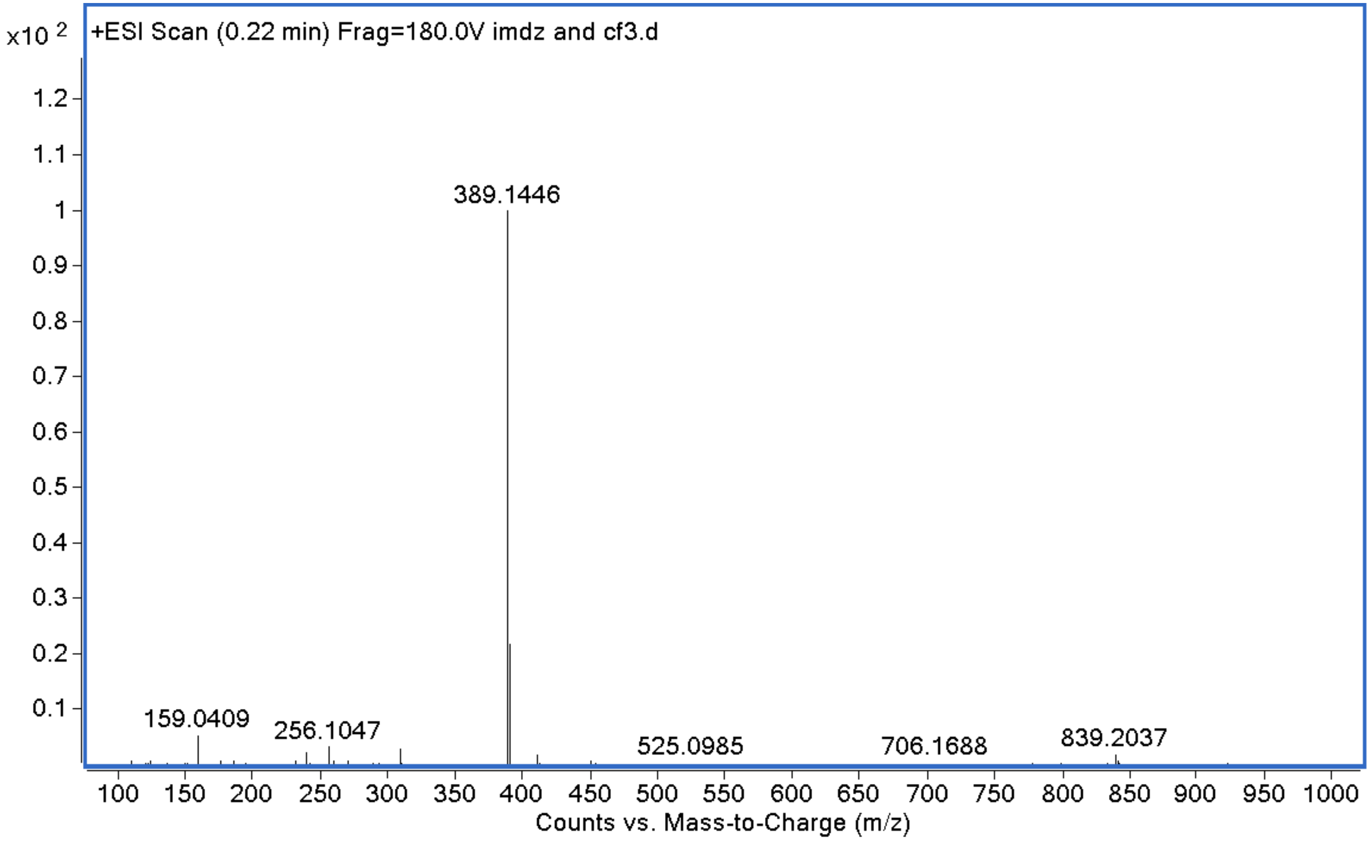

Binding spectra of compound **14** in CYP3A4

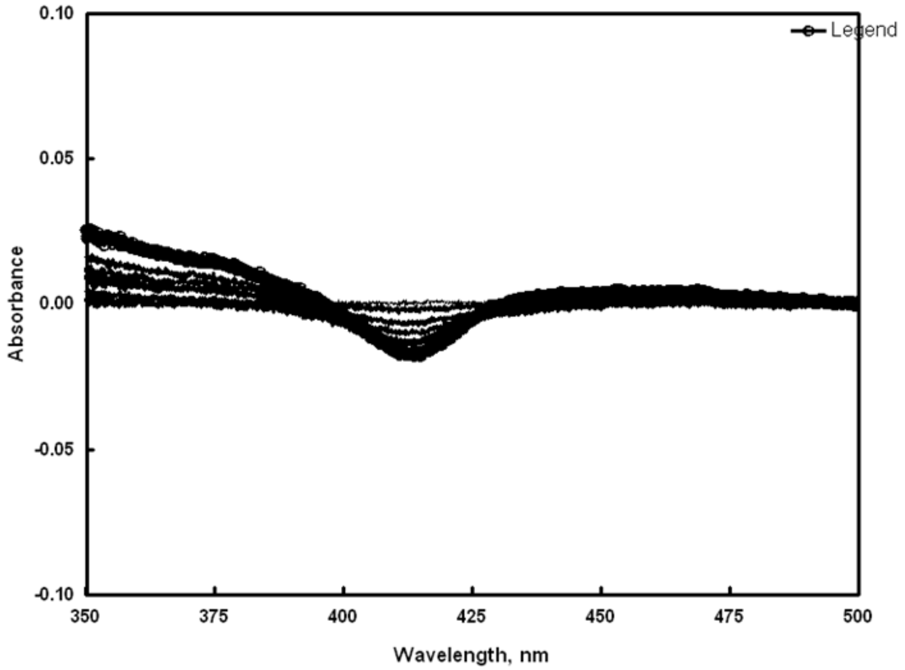

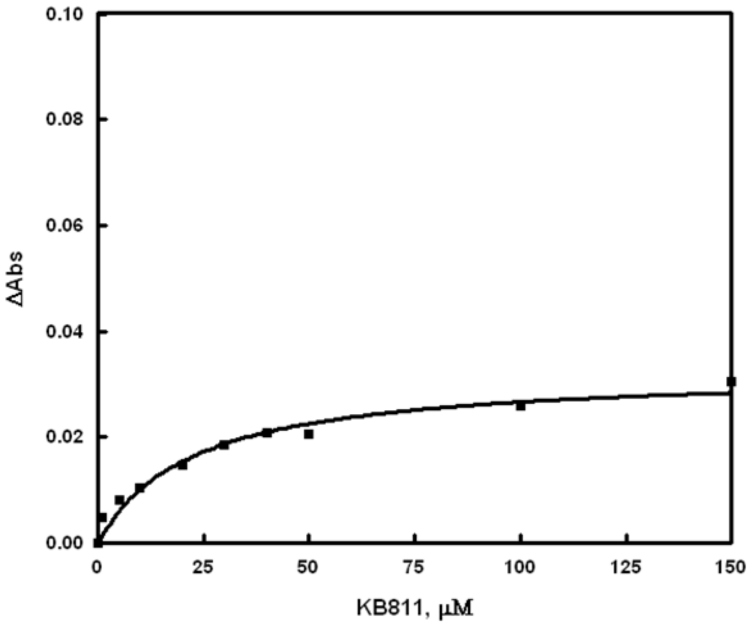

^{1}H NMR spectra of compound **15** measured in MeOH-d_4 at 300 MHz

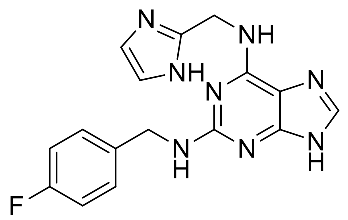

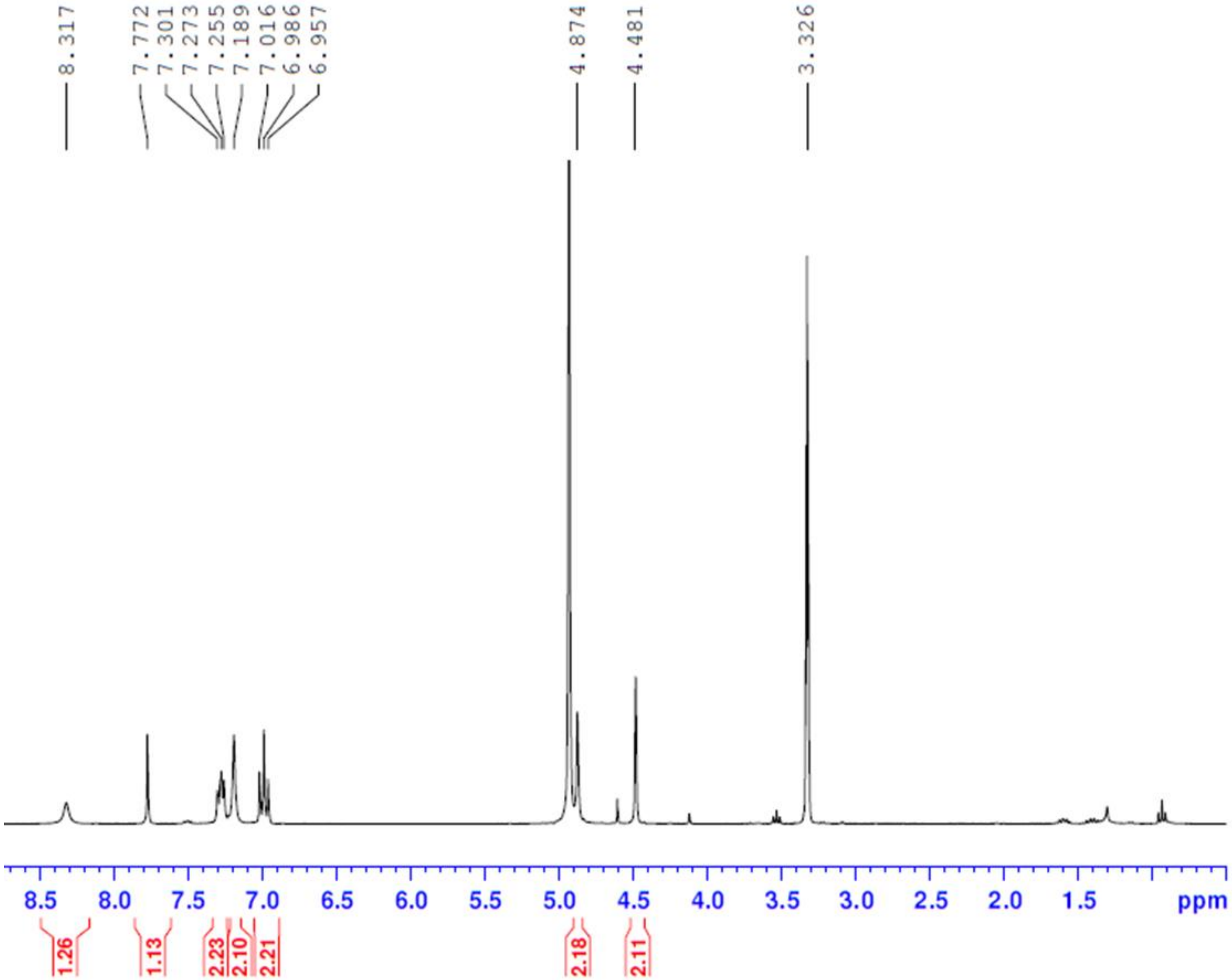

HRMS spectra of compound **15**

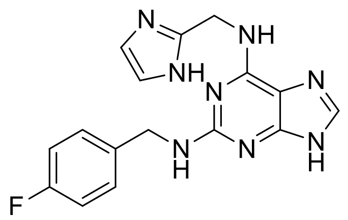

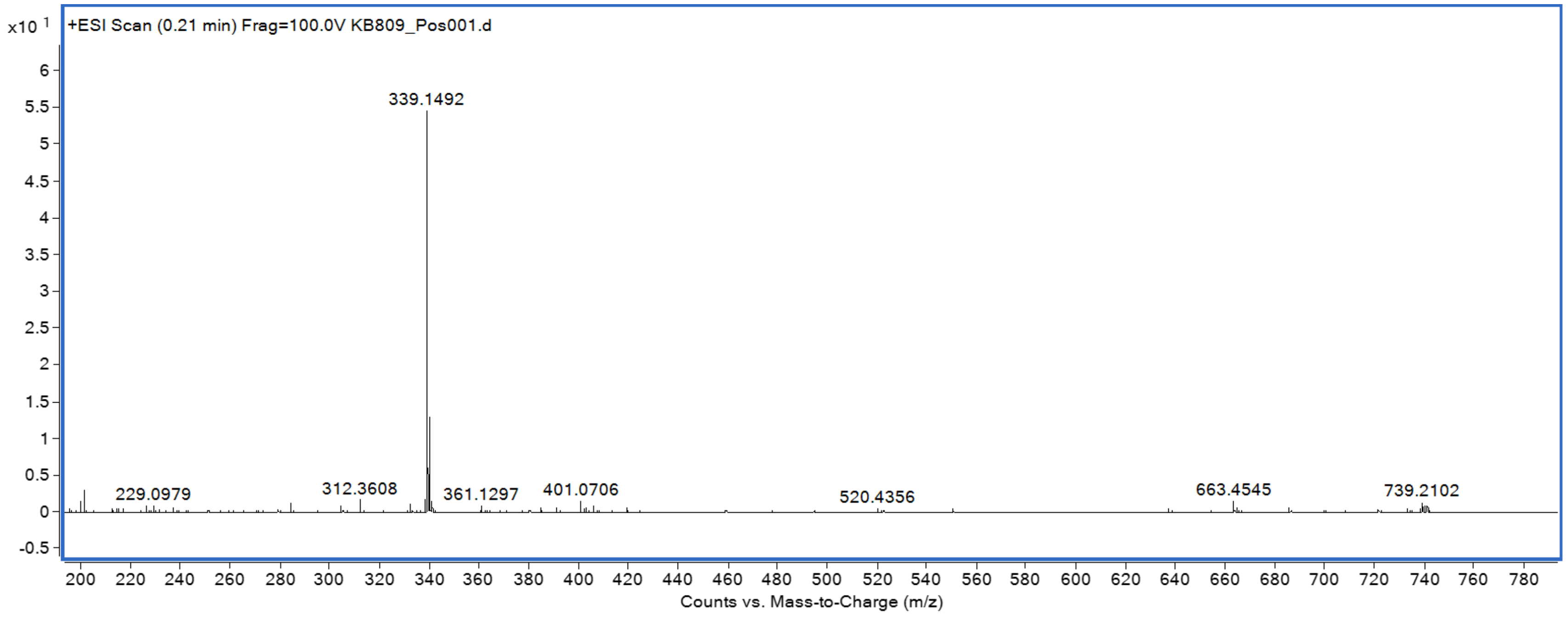

Binding spectra of compound **15** in CYP3A4

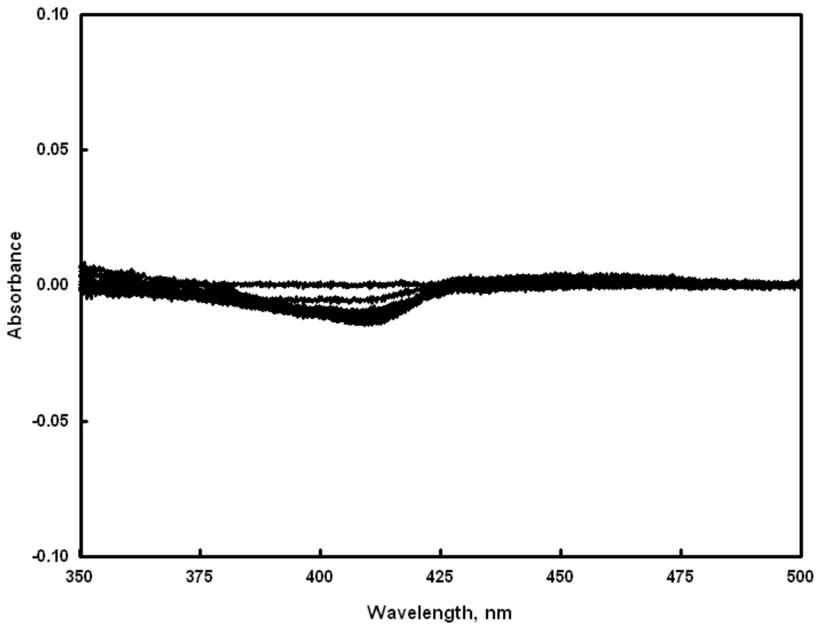

^{1}H NMR spectra of compound **16** measured in MeOH-d_4 at 300 MHz

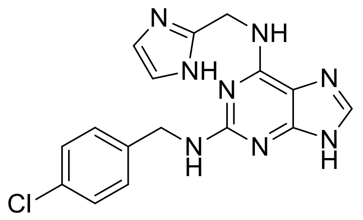

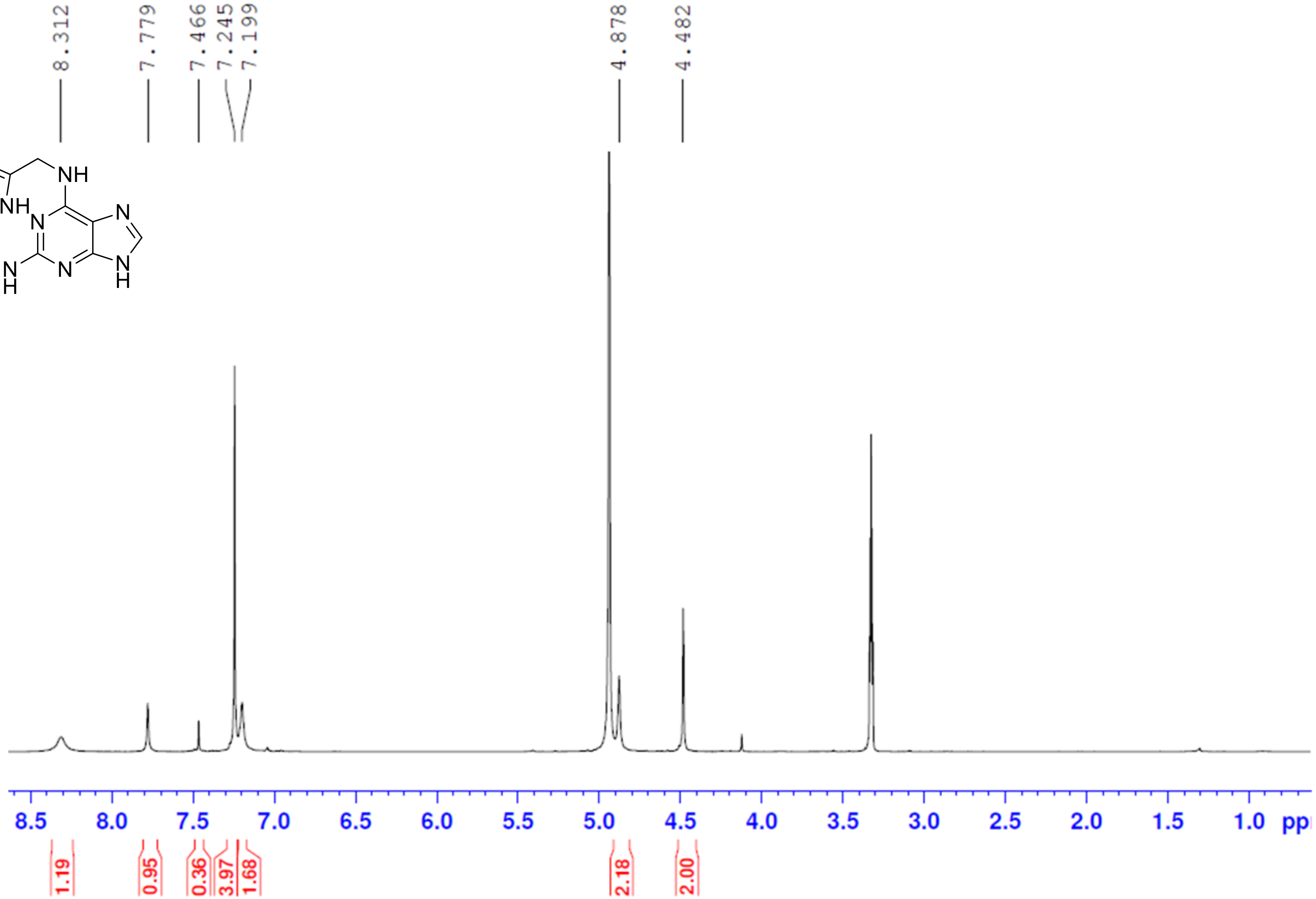

HRMS spectra of compound **16**

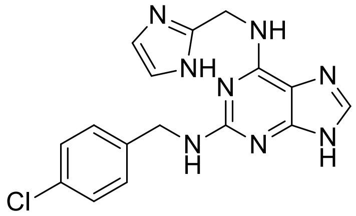

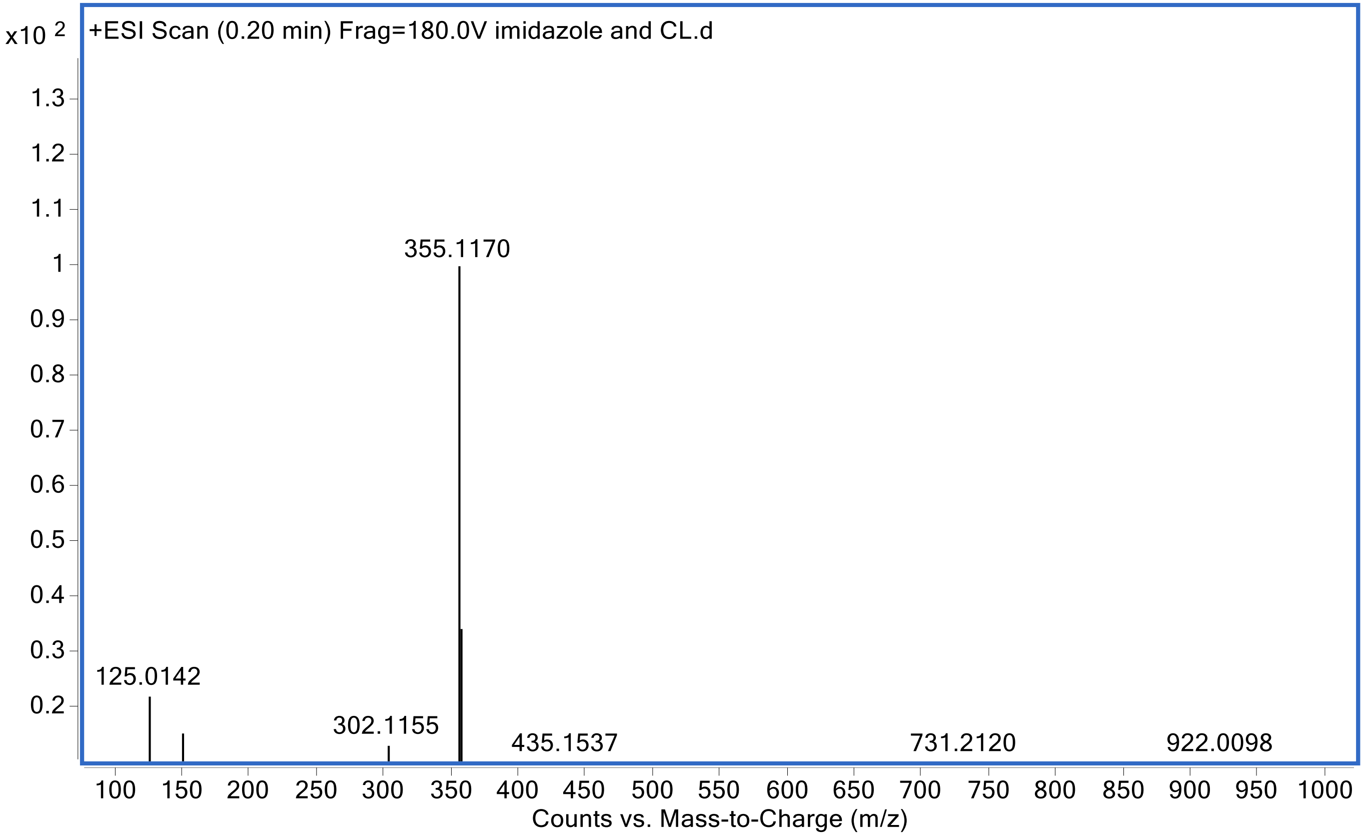

Binding spectra of compound **16** in CYP3A4

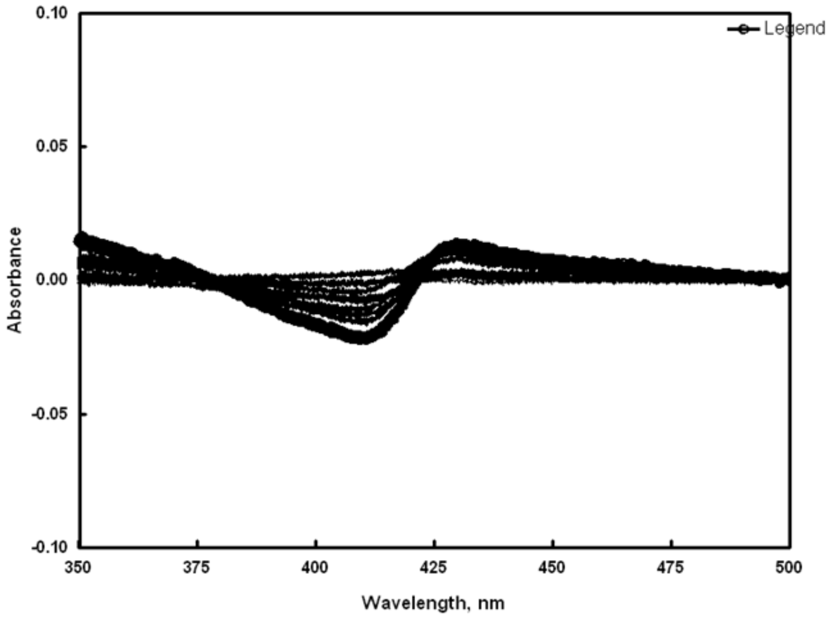

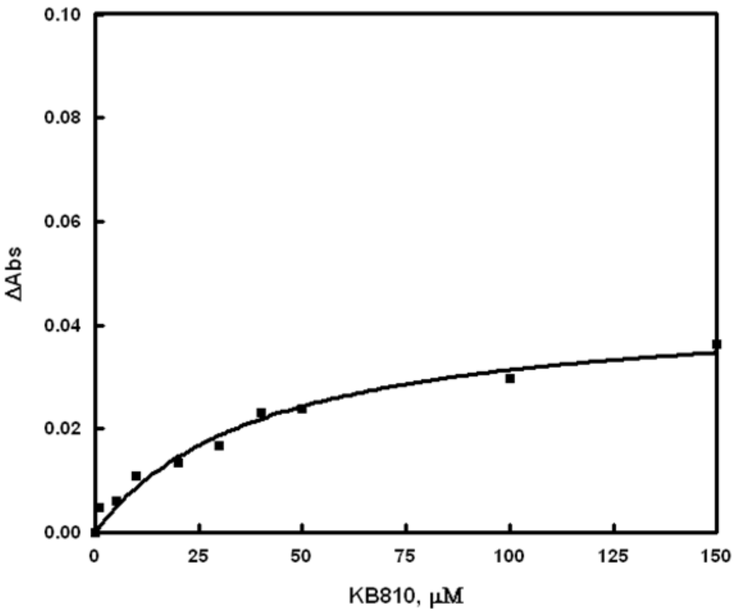

^{1}H NMR spectra of compound **17** measured in MeOH-d_4 at 300 MHz

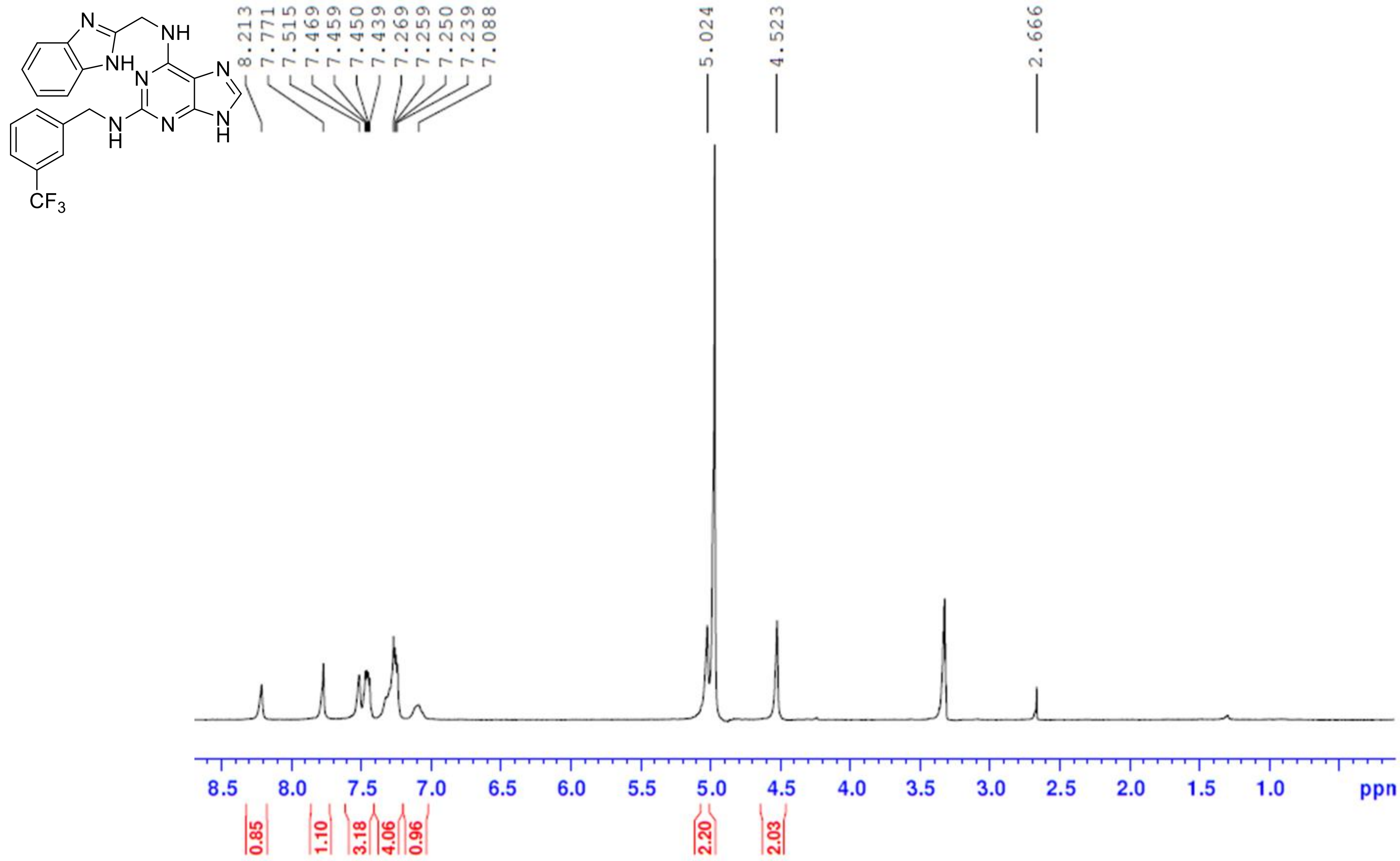

HRMS spectra of compound **17**

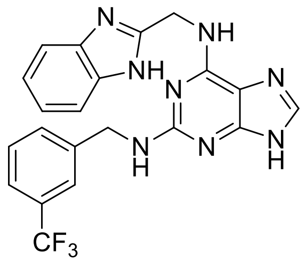

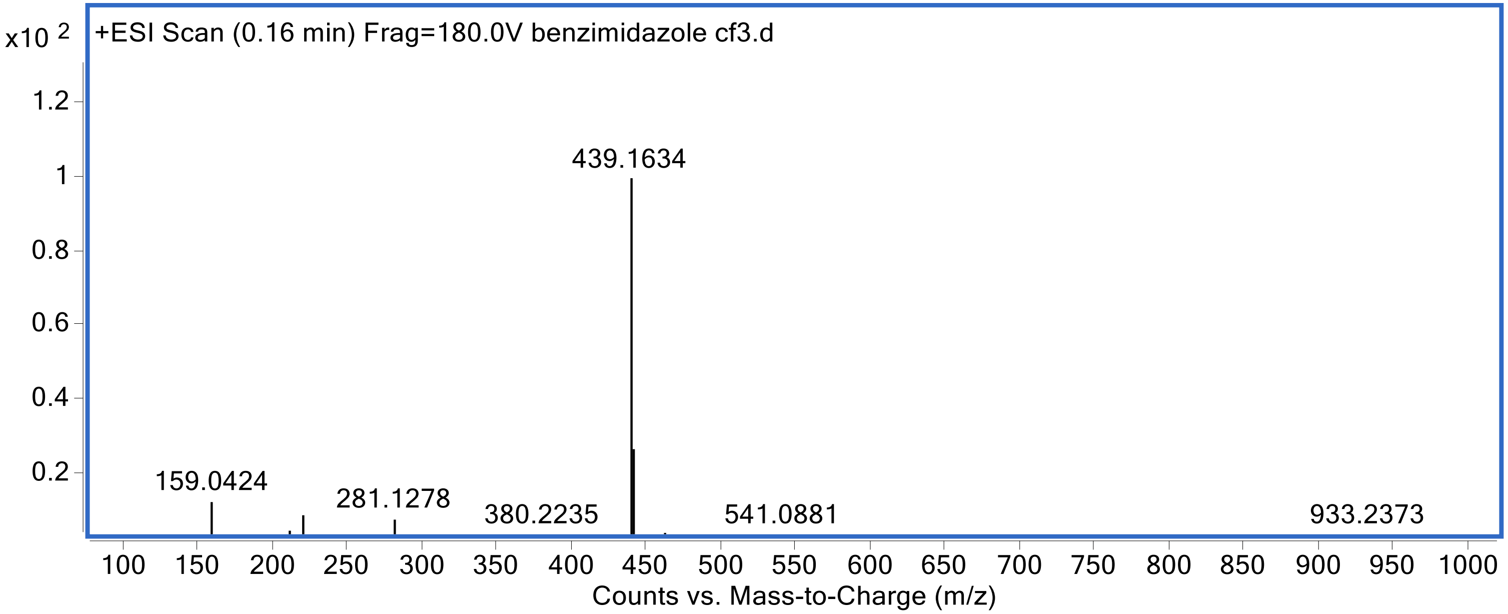

Binding spectra of compound **17** in CYP3A4

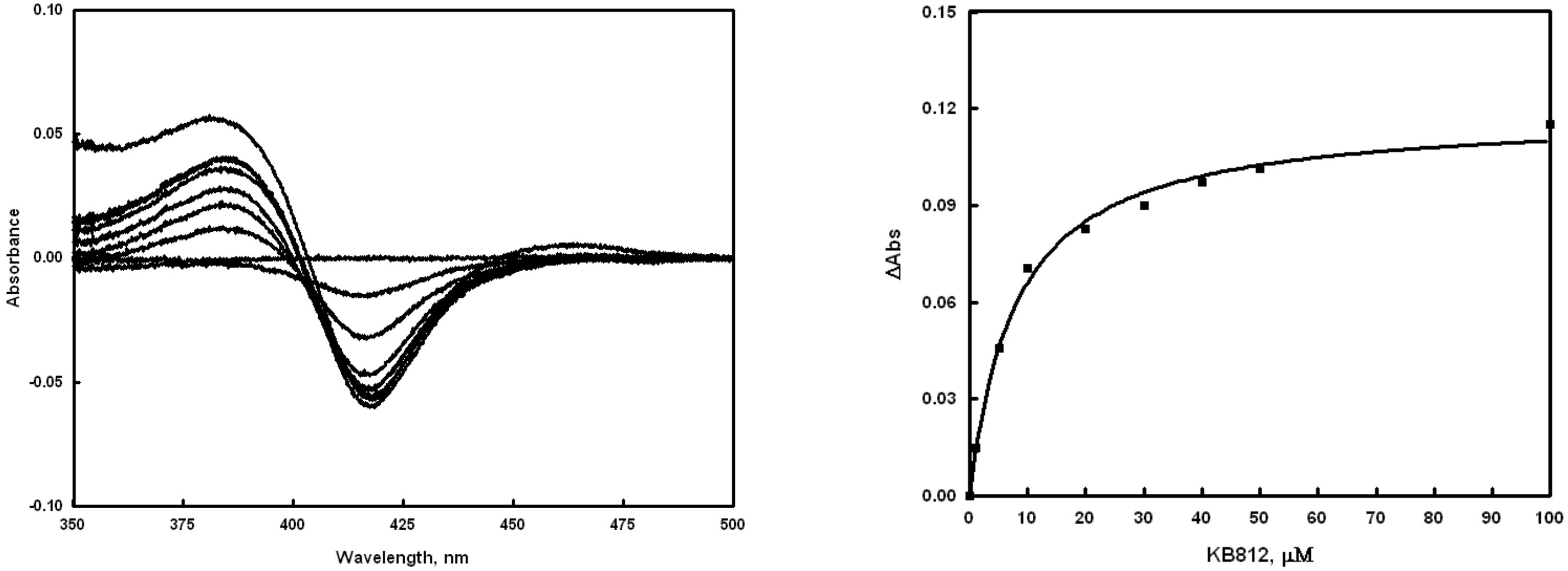

^{1}H NMR spectra of compound **18** measured in MeOH-d_4 at 300 MHz

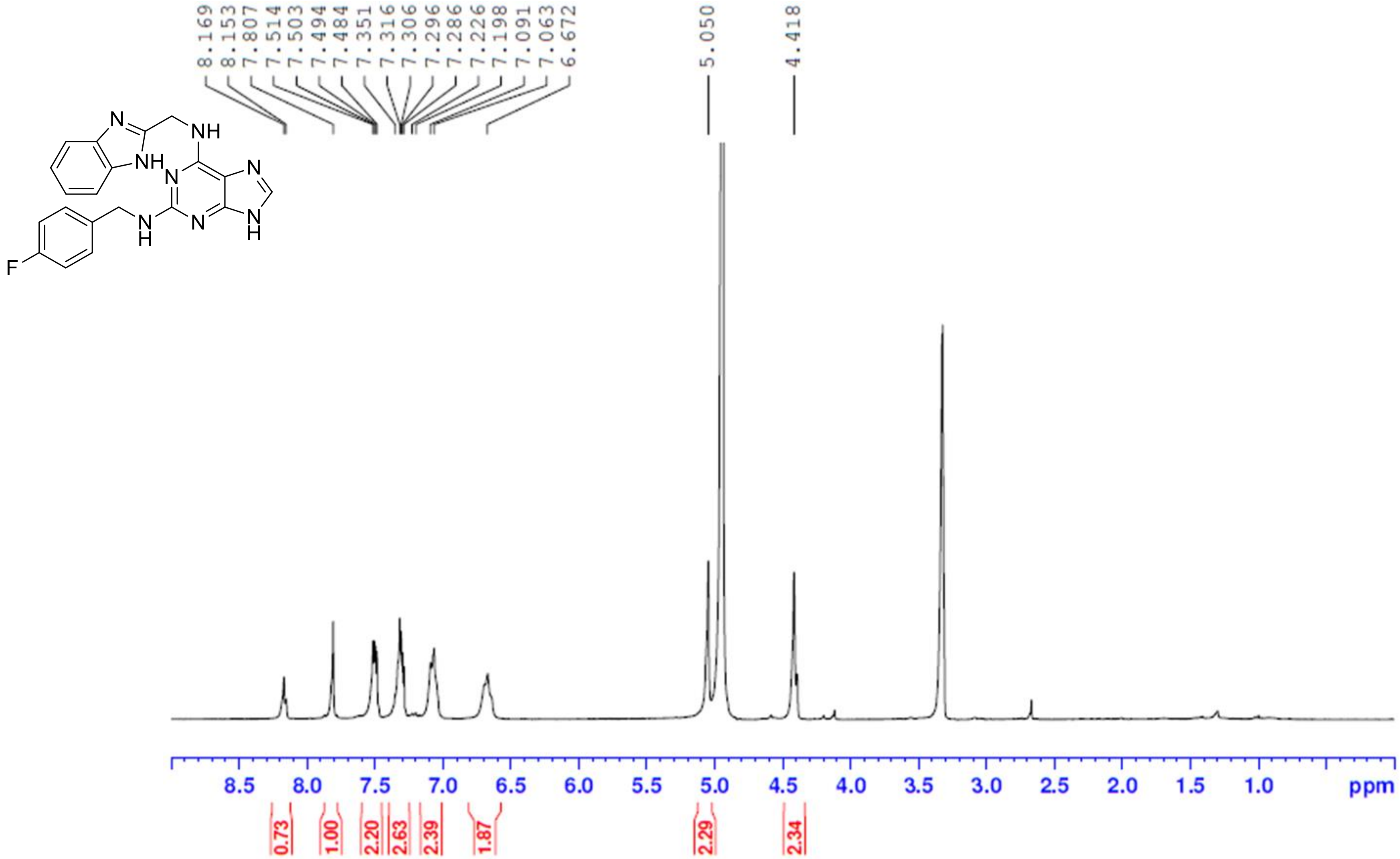

HRMS spectra of compound **18**

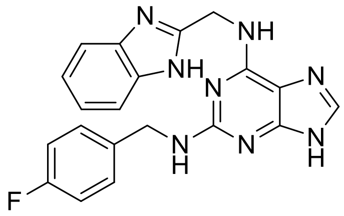

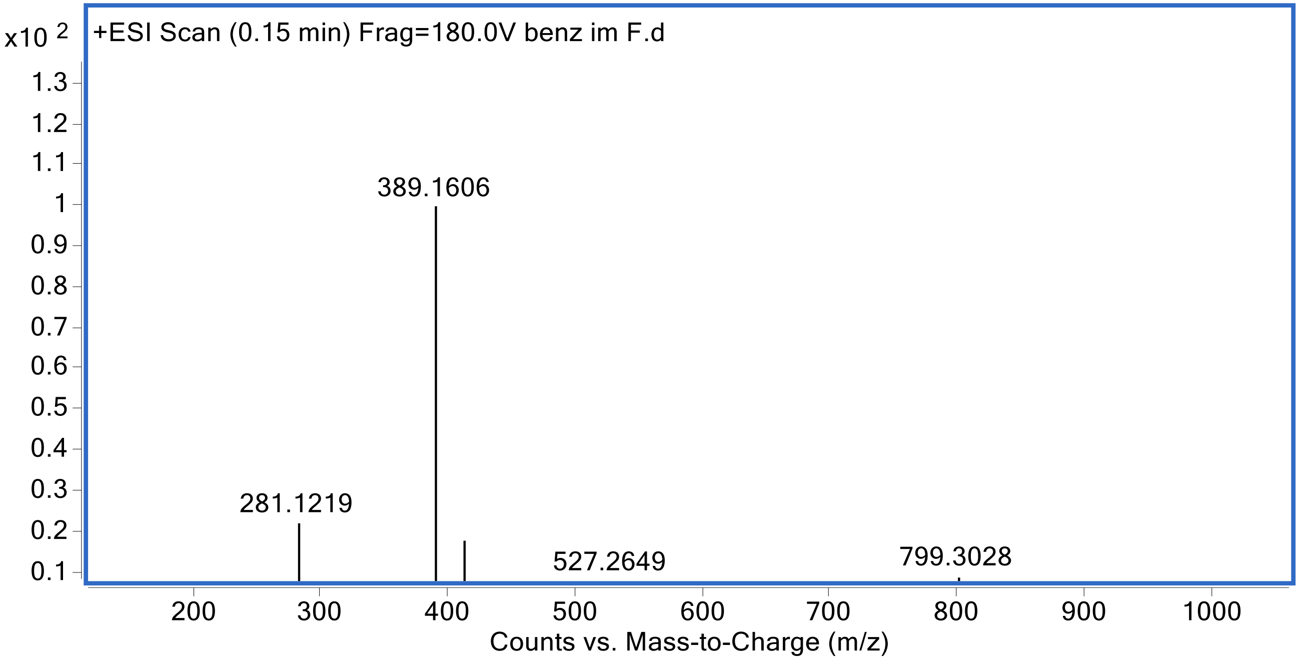

Binding spectra of compound **18** in CYP3A4

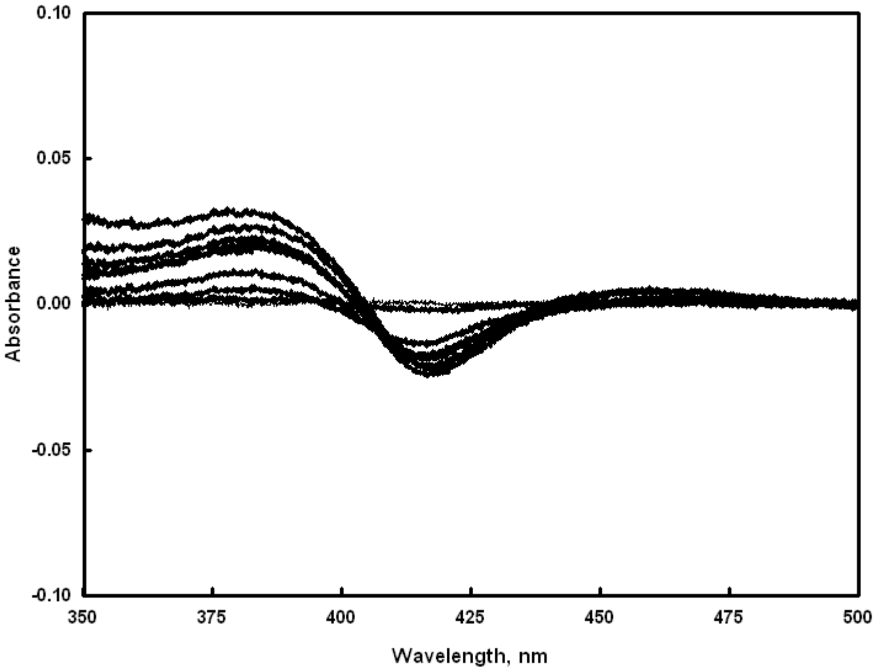

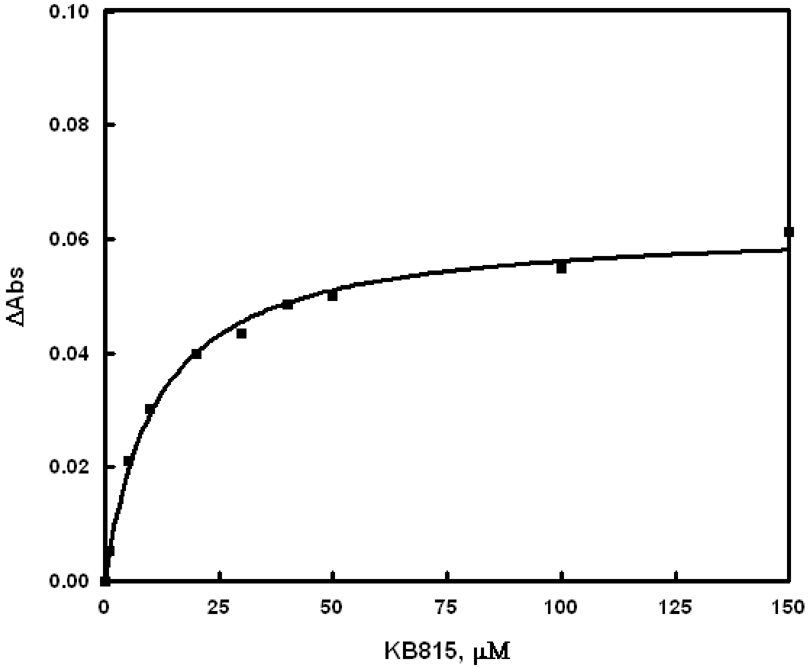

^{1}H NMR spectra of compound **19** measured in MeOH-d_4 at 300 MHz

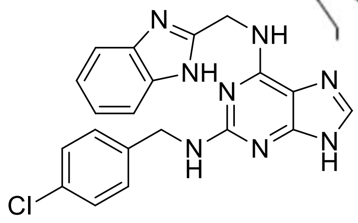

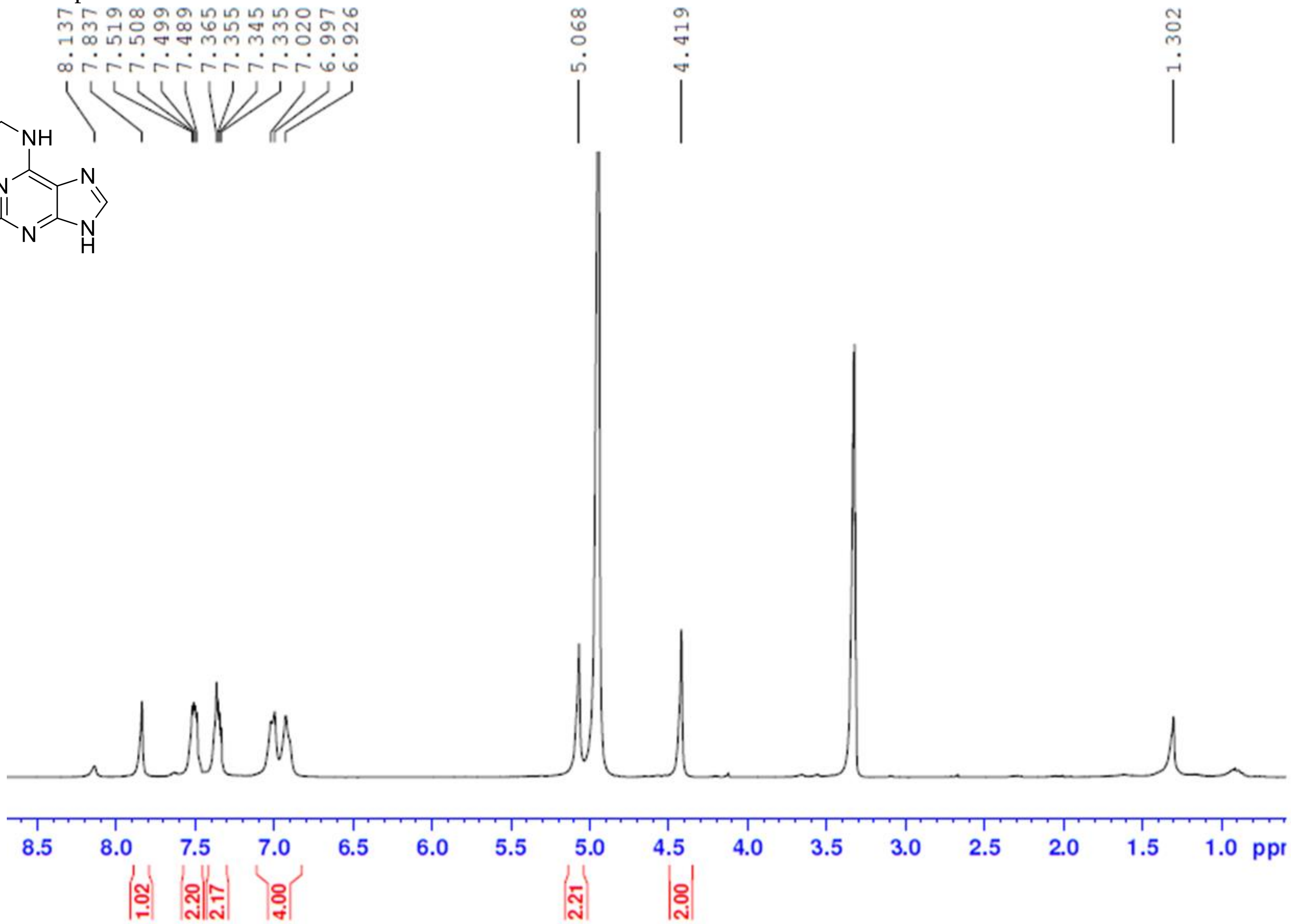

HRMS spectra of compound **19**

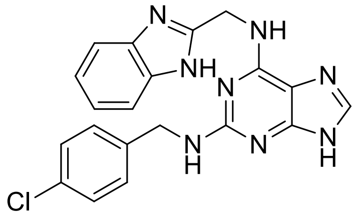

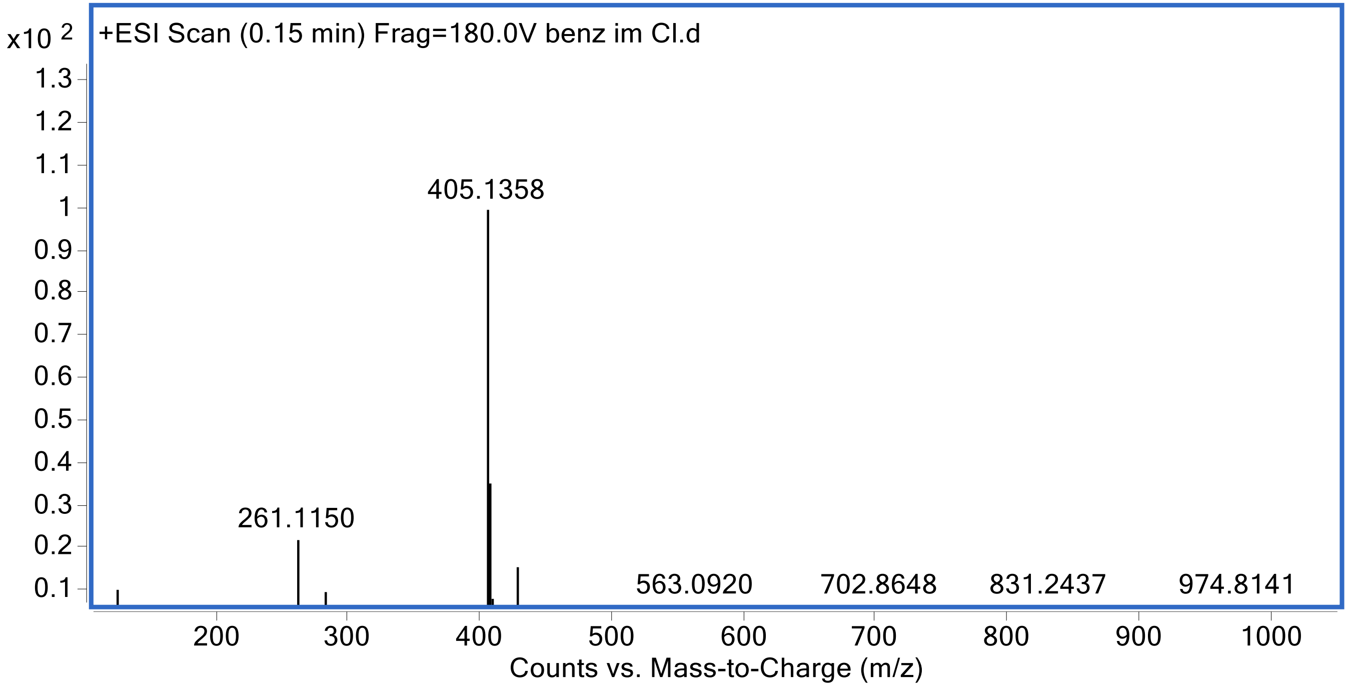

Binding spectra of compound **19** in CYP3A4

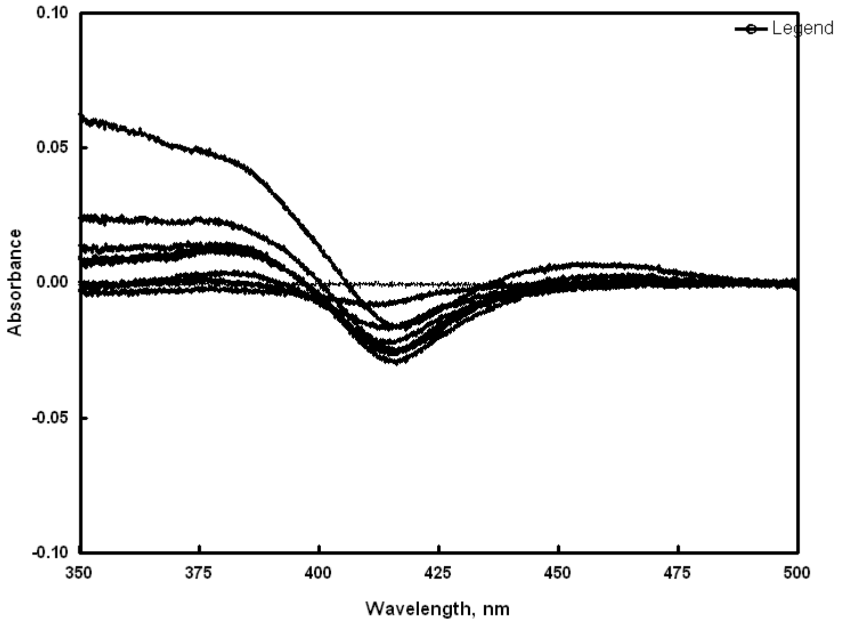

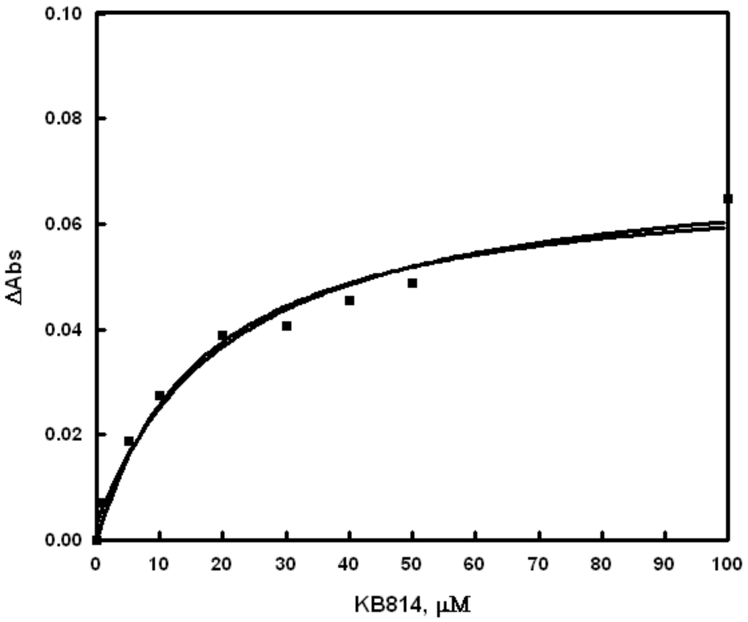

^{1}H NMR spectra of compound **20** measured in MeOH-d_4 at 300 MHz

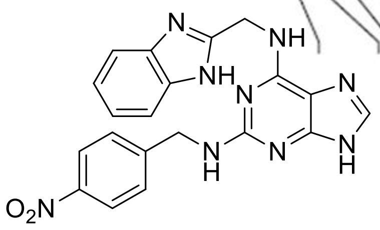

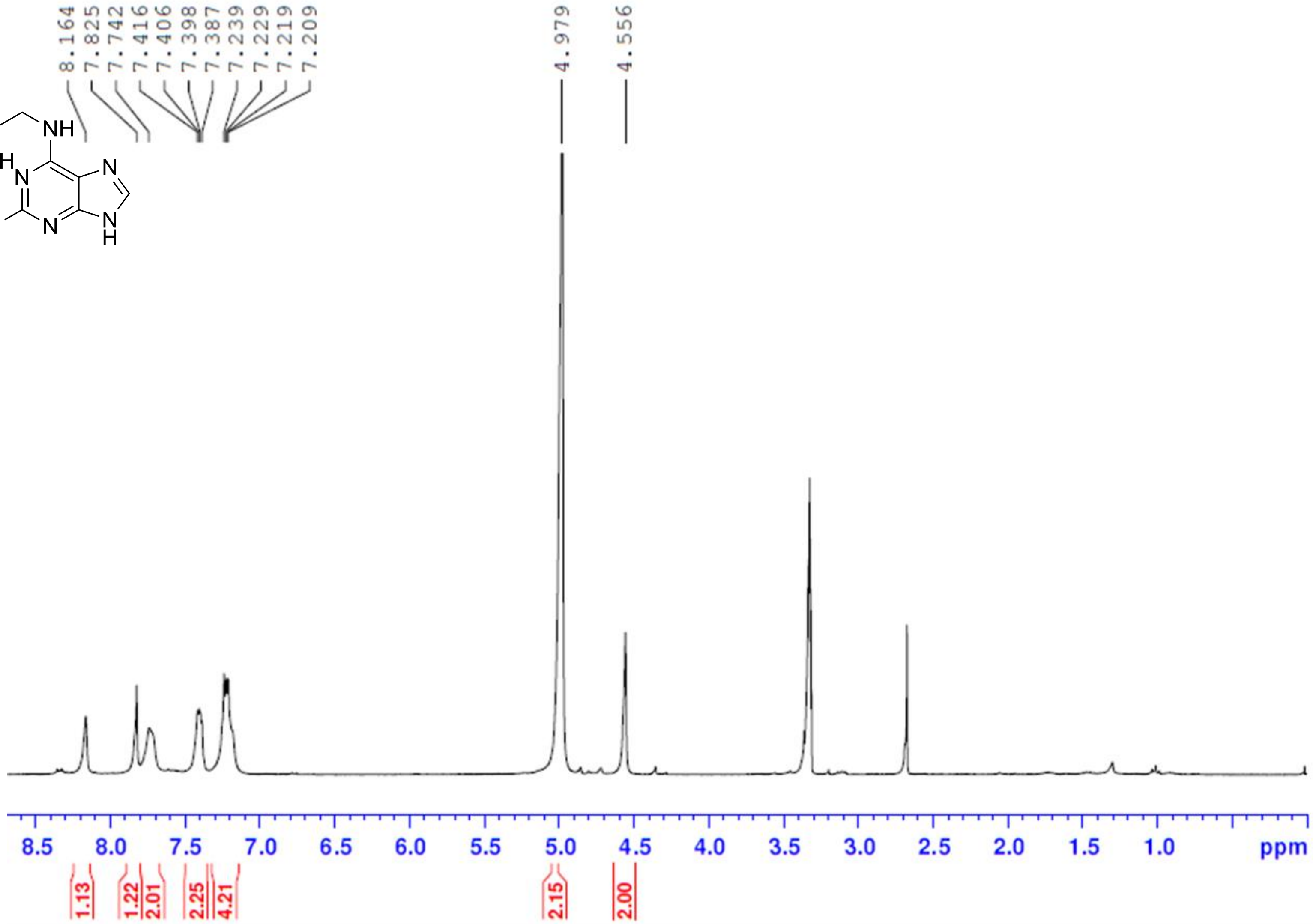

HRMS spectra of compound **20**

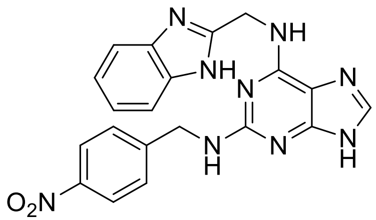

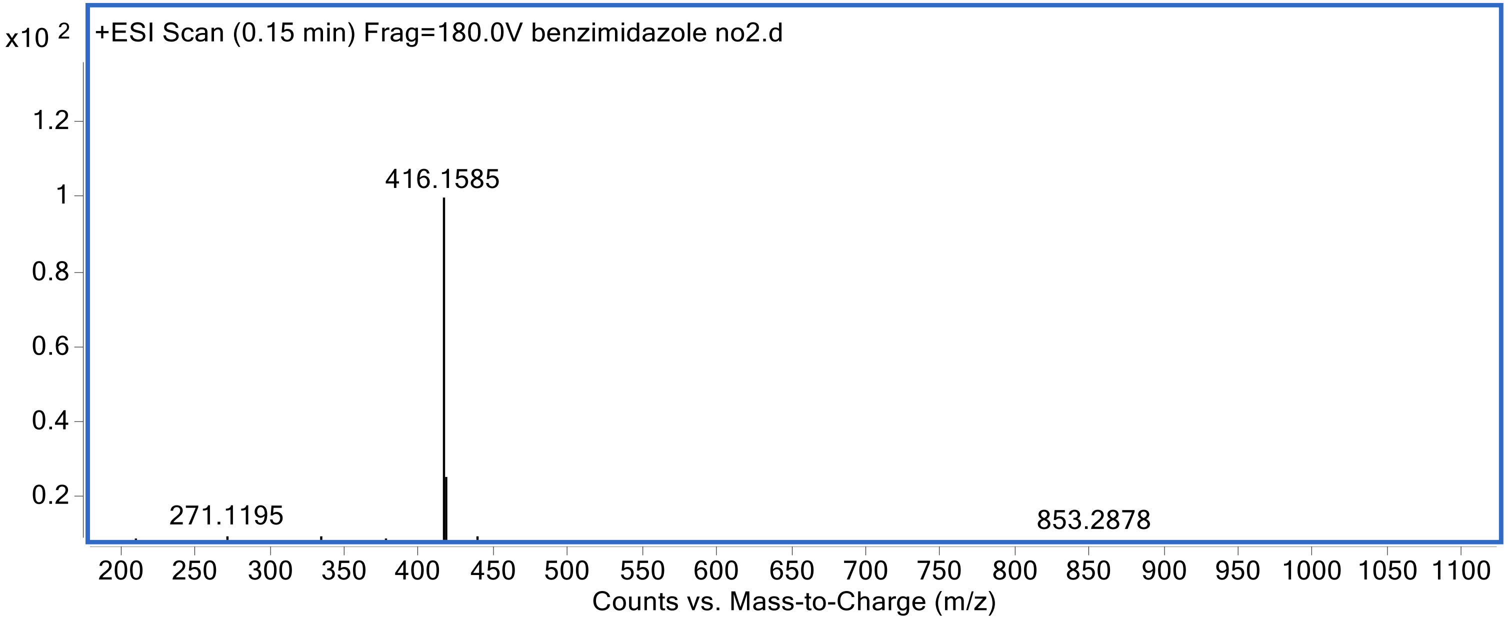

Supplement: Supplemental Material [file IENZ_A_2000404_SM9760.pdf]
